# What’s Going On With Me and How Can I Better Manage My Health? The Potential of GPT-4 to Transform Discharge Letters Into Patient-Centered Letters to Enhance Patient Safety: Prospective, Exploratory Study

**DOI:** 10.2196/67143

**Published:** 2025-01-21

**Authors:** Felix Eisinger, Friederike Holderried, Moritz Mahling, Christian Stegemann–Philipps, Anne Herrmann–Werner, Eric Nazarenus, Alessandra Sonanini, Martina Guthoff, Carsten Eickhoff, Martin Holderried

**Affiliations:** 1 Department of Diabetology, Endocrinology, Nephrology University of Tübingen Tübingen Germany; 2 Tübingen Institute for Medical Education University of Tübingen Tübingen Germany; 3 Department of Medical Strategy, Process and Quality Management University Hospital Tübingen Tübingen Germany; 4 Institute for Bioinformatics and Medical Informatics University of Tübingen Tübingen Germany

**Keywords:** GPT-4, patient letters, health care communication, artificial intelligence, patient safety, patient education

## Abstract

**Background:**

For hospitalized patients, the discharge letter serves as a crucial source of medical information, outlining important discharge instructions and health management tasks. However, these letters are often written in professional jargon, making them difficult for patients with limited medical knowledge to understand. Large language models, such as GPT, have the potential to transform these discharge summaries into patient-friendly letters, improving accessibility and understanding.

**Objective:**

This study aims to use GPT-4 to convert discharge letters into more readable patient-centered letters. We evaluated how effectively and comprehensively GPT-4 identified and transferred patient safety–relevant information from the discharge letters to the transformed patient letters.

**Methods:**

Three discharge letters were created based on common medical conditions, containing 72 patient safety–relevant pieces of information, referred to as “learning objectives.” GPT-4 was prompted to transform these discharge letters into patient-centered letters. The resulting patient letters were analyzed for medical accuracy, patient centricity, and the ability to identify and translate the learning objectives. Bloom’s taxonomy was applied to analyze and categorize the learning objectives.

**Results:**

GPT-4 addressed the majority (56/72, 78%) of the learning objectives from the discharge letters. However, 11 of the 72 (15%) learning objectives were not included in the majority of the patient-centered letters. A qualitative analysis based on Bloom’s taxonomy revealed that learning objectives in the “Understand” category (9/11) were more frequently omitted than those in the “Remember” category (2/11). Most of the missing learning objectives were related to the content field of “prevention of complications.” By contrast, learning objectives regarding “lifestyle” and “organizational” aspects were addressed more frequently. Medical errors were found in a small proportion of sentences (31/787, 3.9%). In terms of patient centricity, the patient-centered letters demonstrated better readability than the discharge letters. Compared with discharge letters, they included fewer medical terms (132/860, 15.3%, vs 165/273, 60/4%), fewer abbreviations (43/860, 5%, vs 49/273, 17.9%), and more explanations of medical terms (121/131, 92.4%, vs 0/165, 0%).

**Conclusions:**

Our study demonstrates that GPT-4 has the potential to transform discharge letters into more patient-centered communication. While the readability and patient centricity of the transformed letters are well-established, they do not fully address all patient safety–relevant information, resulting in the omission of key aspects. Further optimization of prompt engineering may help address this issue and improve the completeness of the transformation.

## Introduction

Ensuring patient safety is fundamental in health care. A key aspect of patient safety is adherence to treatments and interventions prescribed by medical providers, as it is essential for preventing long-term disease progression, reducing complications, and improving quality of life [1]. However, in clinical practice, nonadherence remains a widespread problem. According to the World Health Organization (WHO), only 50% of patients with chronic diseases in developed countries adhere to their prescribed therapy regimens [2]. A common factor driving nonadherence is patients’ lack of understanding of their disease and the underlying principles of therapy [3]. Historically, the patient-health care worker relationship followed a “paternalistic” model, where the patient was a “passive spectator in their own healing process” [4]. Fortunately, this dynamic has changed in recent years. Nonetheless, empowering patients to comprehensively understand their individual health issues remains a promising approach to improving adherence and, consequently, promoting patient safety [3].

For hospitalized patients, the discharge letter is an important source of medical information that complements the conversation with the physician. It also plays a vital role in facilitating communication between hospital doctors and other health care providers, such as primary care physicians [5]. Effective communication during the transition from inpatient to outpatient care is essential for ensuring patient safety [6]. Forster et al [7] found that 59% of preventable adverse events after hospital discharge were attributed to poor communication between hospital caregivers and either the patient or the primary care physician. After discharge, patients with chronic conditions, in particular, face numerous self-management challenges, such as adhering to prescribed medication regimens, maintaining a specific diet, and engaging in physical activity. Failure to adhere to these aspects can result in serious health consequences.

However, in clinical practice, discharge letters are typically addressed to general practitioners or other medical professionals and are often laden with professional jargon, making them inaccessible to patients with limited medical knowledge [8]. Developing patient-centered discharge letters with improved readability has been shown to enhance patient understanding [9,10]. However, in the hospital setting, time for preparing additional, individualized patient letters is often limited, as a significant portion of working hours is devoted to nonpatient-related tasks and documentation [11].

In this context, advances in artificial intelligence (AI) offer a promising approach to providing personalized and scalable support for helping patients understand medical information. Various studies have demonstrated the substantial medical knowledge of large language models (LLMs), such as GPTs [12-15]. Zaretsky et al [16] utilized LLMs to translate discharge summaries into patient-friendly language, addressing common readability metrics and the Patient Education Materials Assessment Tool (PEMAT) scoring. However, they encountered significant limitations in both accuracy and completeness [16]. By contrast, the use of readability scores has been a topic of controversy in the literature [17]. In our own work, we demonstrated GPT-4’s ability to answer psychosomatic medicine examination questions [18]. A qualitative analysis of incorrect answers, based on Bloom’s revised taxonomy [19,20], revealed that errors varied depending on the cognitive level. It remains unclear whether this effect is also observed in patient letters generated by GPT-4. Additionally, the extent to which GPT-4 addresses comprehensive patient safety–relevant information—a key aspect of discharge letters—has yet to be clarified.

In this study, we further investigated a related topic. We used GPT-4 to transform discharge letters into accessible, patient-centered letters and evaluated its ability to identify and incorporate patient safety–relevant information. To pinpoint potential errors or gaps in the AI-driven transformation process, patient safety–relevant information was categorized and analyzed based on Bloom’s revised taxonomy (Remember, Understand) [19,20].

In summary, our study addresses the following questions:

Major objective:

- How comprehensively does GPT-4 identify and transform patient safety–relevant information, as measured by the learning objectives, from discharge letters into patient-centered letters?

Minor objectives:

- How do GPT-4–generated patient-centered letters perform in terms of medical correctness, measured by medical accuracy, case-specific relevance, and the sources of information used in the constituent sentences?
- How well do GPT-4–generated patient-centered letters perform in terms of patient centricity compared with discharge letters, with patient-centered language measured by standard readability scores, word and sentence count, and the use of medical jargon, explanations, abbreviations, repetitions, and direct addressing?

## Methods

### Study Outline

**Figure 1:**
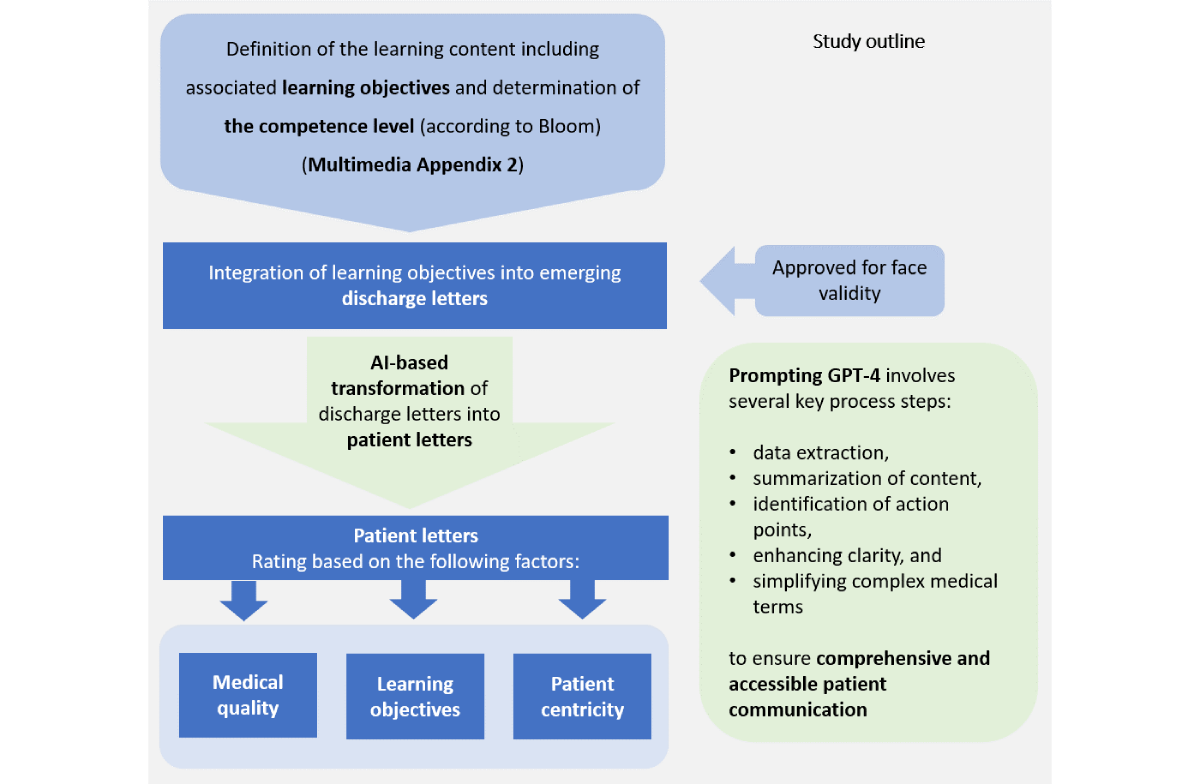
Comprehensive overview of the study’s structure and progression. Prompting steps are illustrated in the green box. AI: artificial intelligence.

As the primary goal of patient-centered letters is to convey important information to patients in alignment with specific didactic “learning objectives,” we defined learning objectives for 3 common medical conditions, including the corresponding competence levels based on Bloom’s taxonomy. Using these learning objectives and associated medical conditions, we created 3 discharge letters (Figure 1 and Multimedia Appendix 1). We then prompted GPT-4 to generate a patient-centered letter for each discharge letter (Multimedia Appendix 1). To account for variability in GPT-4’s output, we repeated the generation of each patient-centered letter 5 times using the same prompt.

The resulting patient-centered letters were analyzed by a team of 2 experienced clinicians (FE and FH) in terms of medical quality, patient centricity, and their potential to convey safety-relevant medical information. The discharge letters, the GPT-4 prompt, and the processing of the prompts by GPT-4 to create the patient-centered letters were all conducted in German. DeepL Translate (DeepL SE) was used to translate the discharge letters, patient letters, and the prompts (Multimedia Appendix 1).

### Development of Discharge Letters

For the development of the discharge letters, we meticulously structured a multistep process (Multimedia Appendix 2) to cluster critical patient information, which GPT-4 was prompted to extract from the discharge letters at various structural levels. These levels enabled us to assess GPT-4’s effectiveness in retrieving critical patient information during the evaluation process based on multiple criteria. This approach provided a clearer depiction of GPT-4’s competence in this specific task area. To demonstrate the transferability of our findings, it was essential to establish the extent to which GPT-4’s ability to identify patient-important information was context-independent. Accordingly, we developed 3 distinct scenarios, each with different disease profiles and care settings.

Stepwise approach:

- Step 1: We selected 3 common diseases based on their high prevalence—arterial hypertension (AHT), type 2 diabetes mellitus (DM), and diabetic kidney disease (DKD).
- Step 2: The setting for each scenario was chosen, encompassing either outpatient consultations with diagnostics or inpatient treatment for initial diagnosis or follow-up care.
- Step 3: To construct relevant educational content for each scenario, we aligned all learning content with the cognitive process dimension of factual knowledge in Bloom’s revised taxonomy [20].
- Step 4: The educational content was then categorized into 4 distinct fields: (1) organizational aspects, (2) medication protocols, (3) prevention of complications, and (4) disease management and lifestyle changes.
- Step 5: For each piece of learning content, we developed 2 corresponding learning objectives (Multimedia Appendix 1): one at the Remember level (eg, the patient knows they need to conduct a 24-hour urine collection test), and another at the Understand level (eg, the patient understands that the 24-hour urine collection is necessary to rule out hormonal causes of hypertension). Each discharge letter thus comprised 12 pieces of learning content, distributed across the 4 content fields, resulting in 24 learning objectives per letter.
- Step 6: These learning objectives were then systematically integrated into the format of a typical discharge letter. The discharge letters were developed by 2 experienced physicians (FE and FH) and validated for face validity by 2 additional board-certified internal medicine and nephrology specialists (MM and MG).

### Prompt Development and Creation of Patient-Centered Letters

To the best of our knowledge, there is currently no ideal model for the structure of patient-centered letters. Our group focused first on expert-formulated learning objectives based on content deemed important from a medical perspective, and second on patient-centered language. This approach provided us with a basic letter structure to aim for.

We utilized GPT-4 (gpt-4-0613, accessed December 2023) to generate the patient-centered letters, maintaining the model’s default parameters and setting the temperature to the default value of 1.0. The original prompt can be found in Multimedia Appendix 1. The AI-generated patient-centered letter development process was divided into multiple stages (Textbox 1; Multimedia Appendix 3), leveraging methodologies involving LLMs as agents.

Further refinement involved adversarial prompting to identify and simplify complex medical terms and jargon, tailoring the content to a comprehension level equivalent to an eighth-grade reading ability [21]. The result of this process was a restructured and more comprehensible summary of actionable points, designed to effectively communicate essential medical information to patients.

**Stages in the artificial intelligence–generated patient-centered letter development process.**

**1. Stage 1**

- Essential descriptions from the original discharge letters were transformed into concise, comprehensible summaries, referred to as “structured info,” using a specialized system prompt for data extraction.

**2. Stage 2**

- A general summary of the original letter was created to provide an overview of the information, using a straightforward system prompt without specific constraints to minimize redundancy.

**3. Stage 3**

- This final stage focused on extracting detailed “action points” critical for patient understanding and compliance.
- This multistep process, known as “prompt chaining,” began with segmenting the original letter to isolate key instructions and reasons for necessary behavioral changes.
- Each segment was processed to reduce redundancy and enhance clarity, using structured prompts to request information in a predefined JSON format.

### Ethical Considerations

This study was approved by the local ethics committee (approval number 778/2023BO2) of the University Hospital of Tübingen. As the discharge letters were prepared by a team of medical experts based on general clinical cases and without direct reference to specific patients, informed consent was not required. As the discharge letters do not contain any individual patient data that could be traced back to specific persons, anonymization was also unnecessary. Furthermore, no compensation payments are expected due to the chosen methodological approach.

### Analysis of Patient-Centered and Discharge Letters

#### Rating Process

Two experienced clinicians (FE and FH) independently rated the discharge letters and the patient-centered letters. For the rating process, each letter was divided into sentences. A sentence was defined as a unit of 1 or more words ending with a period, colon, or a new paragraph (typically, but not exclusively, representing complete sentences). Specific titles in the patient-centered letters, such as “Main Diagnosis,” were predefined using established terms and excluded from the rating.

Each sentence was assessed individually by both raters. To ensure a standardized rating process, a general rating structure was defined in advance, using an ordinal scale from 0 (not fulfilled) to 2 (fully fulfilled; Tables 1 and 2). In cases of uncertainty, the raters discussed the issues until a consensus was reached. The results are presented as overall comparisons (patient-centered letters vs discharge letters) and as a subgroup analysis (subgroups: letters on AHT, DM, and DKD).

**Table 1:** Schematic rating scale (learning objectives).^a^

Content field	Common disease, Bloom’s category, learning objective, and rating examples			
Medication	**Type 2 diabetes mellitus**			
		Remember	Take new medication atorvastatin 20 mg in the morning	2: “One tablet (20mg) atorvastatin in the morning (...)” 1: “Atorvastatin: Take one tablet every morning.”
		Understand	Atorvastatin helps to control lipid levels.	2: “Atorvastatin: (...) can help keep cholesterol low and protect your heart.” 1: “You have also been given medication to lower high cholesterol, which is important in reducing the risk of heart disease.”
Prevention of complications	**Diabetic nephropathy**			
		Remember	Avoid NSAIDs^b^ such as ibuprofen	2: “Ibuprofen: You should avoid this painkiller (…).” 1: “It is important for you to avoid anti-inflammatory pain medications.”
		Understand	Avoiding NSAIDs is important to prevent kidney damage	2: “Ibuprofen: (…) as it can impair your kidneys.” 1: “You had been taking painkillers regularly, which may have also strained your kidneys.”

^a^This is an illustration of the rating scale used to analyze the learning objectives. It includes rating examples of learning objectives that were either fully (2) or partially (1) rendered in the patient-centered letters.

^b^NSAID: nonsteroidal anti-inflammatory drug.

**Table 2:** Rating scale (medical quality and patient centricity). The table describes the rating scale used to evaluate the patient-centered letters for medical quality and patient centricity. Examples from the patient-centered letters or discharge letters are presented within quotation marks.

Rating scales			Definition		Example from patient letters or discharge letters
**Medical correctness**					
	Medically correct	The sentence is medically correct.		“It is important that you reduce your alcohol consumption, as alcohol can affect blood sugar levels and increase the risk of other health problems.”	
	Medically incorrect	The sentence is medically not correct (normal HbA_1c_^a^ is below 5.7%).		“The HbA_1c_ value, which tells me how my blood sugar has been over the last few weeks, is 14.1%, which is also very high (normal below 6%).”	
**Case-specific relevance**					
	Very relevant	Information is related to the primary diagnosis.		“During your stay in hospital, you were diagnosed with type 2 diabetes for the first time, which means that your blood glucose levels are too high.”	
	Rather relevant	Information is related to the secondary diagnosis.		“You had an appendectomy in 1972.”	
	Neither/nor relevant	Not attributable to a primary or secondary diagnosis.		“In plain language, your hospital stay can be summarized as follows:”	
**Source of information**					
	Discharge letter	Information is derived from the discharge letter.		“You have chronic kidney disease caused by diabetes and high blood pressure.”	
	Not from discharge letter	Information was added by GPT-4.		“They performed a kidney biopsy in which they obtained a small sample of tissue from your kidney to examine it under a microscope.”	
**Medical terms**					
	Use of the medical term	The medical term is used in the sentence.		“We ask for the completion of a 24h urine collection for metanephrine to investigate a pheochromocytoma.”	
	No special term	No special term is used in the sentence.		“You should also try to reduce your weight, eat more healthily (lots of fruit and vegetables, less fatty dairy products) and use less salt.”	
**Explanations**					
	Medical term explained	The medical term is explained in everyday language.		“Sleep apnea syndrome is a condition in which breathing stops during sleep, which can also affect blood pressure.”	
	Medical terms not explained	Medical term is not explained.		“In addition, sleep apnea syndrome should be ruled out in the outpatient setting.”	
**Abbreviations**					
	Use of abbreviations	An abbreviation is used in the sentence.		“CVRF^b^:”	
	No use of abbreviations	An abbreviation is not used in the sentence.		“This is called hypertension.”	
**Repetitions**					
	Use of repetitions	Repetition of a fact in the sentence.		“It is also recommended that you regularly measure your blood pressure at home (…) Measure your blood pressure regularly at home (…)”	
	No use of repetitions	No repetition of a fact in the sentence.		“Drink about 2 liters of water a day unless your doctor says otherwise.”	
**Addressing**					
	Directly addressed	The patient is directly addressed.		“You should take one tablet every morning.”	
	Not directly addressed	The patient is not directly addressed.		“We therefore recommend initiation of atorvastatin as indicated above.”	
	Not applicable	Not applicable because there is no person to be addressed in the sentence.		“An ultrasound of the pancreas showed no evidence of a malignancy.”	

^a^HbA_1c_: glycated hemoglobin.

^b^CVRF: Cardiovascular Risk Factors.

#### Learning Objectives

The GPT-4–generated patient-centered letters were rated based on how many of the learning objectives were identified and addressed. To better understand GPT-4’s ability to highlight patient safety–relevant information, a descriptive analysis of the learning objectives was performed. This analysis determined whether the learning objectives were fully addressed, partially addressed, or missed entirely (Table 1).

#### Indicators of Patient Centricity

All letters were rated for readability using the Flesch Reading Ease test and the Swedish Läsbarhetsindex (LIX) and the Readability Index (RIX). The Flesch Reading Ease score ranges from 0 (very difficult to read) to 100 (very easy to read) [22]. The Swedish LIX typically ranges from 20 (very easy to read) to 60 (very difficult to read) [23]. The RIX is a modified version of the LIX, with a higher number indicating a more complex text [24]. Because of the fragmented nature of some discharge letter sentences (Multimedia Appendix 1), only the sections on the procedure and the medical discharge summary were used to calculate the readability score. By contrast, patient-centered letters, written in complete sentences, were evaluated as a whole for readability. To identify potential barriers for nonmedical readers, the use of medical terms, explanations of medical terms, abbreviations, and repetitions were analyzed for each sentence (Multimedia Appendix 4). Additionally, it was assessed whether the recipient of the letter (patient-centered letter: patient; discharge letter: general practitioner) was directly addressed in each sentence, where applicable.

#### Medical Correctness, Case-Specific Relevance, and Source of Information

All sentences were rated for medical correctness (Multimedia Appendix 4). To gain deeper insights into medical errors in the patient letters, a qualitative analysis of the incorrect sentences was conducted using the Braun-Clarke inductive approach [25]. Sentences were also evaluated for their case-specific relevance: sentences referring to the primary diagnosis were rated as highly relevant, those related to secondary diagnoses were considered moderately relevant, and all other sentences were classified as neither relevant nor irrelevant, as no irrelevant information was found in the letters. Additionally, it was assessed whether the medical information in the patient letters originated from the discharge letter or was generated by GPT-4.

### Statistical Analysis

Statistical analysis and figure generation were conducted using R statistical software (version 4.3.1; R Foundation for Statistical Computing). Data were presented as total counts (n) and percentages, or, when not normally distributed, as medians with interquartile ranges (25th and 75th percentiles). Decimal numbers were rounded to whole numbers for clarity.

## Results

### Quantitative Analysis of Letter Structure

For the discharge letters, the number of sentences per letter and the median word count per sentence showed minimal variation across the 3 disease entities (Table 3).

For the patient-centered letters, GPT-4 generated a total of 952 sentences, with 860 sentences remaining after excluding those predefined in the prompts. The median word count per sentence was 15 (IQR 8-20) words. The number of sentences per patient-centered letter varied slightly between the medical conditions of AHT, DM, and DKD. The highest number of sentences was recorded in the discharge and patient-centered letters for DKD, while the fewest sentences were found in the patient-centered letters on DM.

**Table 3:** Quantitative analysis of letter structure.

Analysis		Overall	Arterial hypertension	Diabetes mellitus	Diabetic kidney disease
**Number of sentences**					
	Discharge letter, n	273	83	92	98
	Patient letter, n	860	278	271	311
**Sentences per letter**					
	Discharge letter, n	92	83	92	98
	Patient letter, median (IQR)	56 (54-59)	55 (52-57)	54 (53-58)	58 (56-65)
**Words per sentence,** **median (IQR)**					
	Discharge letter	8 (3-13)	9 (4-14)	8 (3-13)	7 (3-13)
	Patient letter	15 (8-20)	15 (9-21)	14 (9-21)	14 (8-19)

### Learning Objectives in GPT-4–Generated Patient-Centered Letters

**Figure 2:**
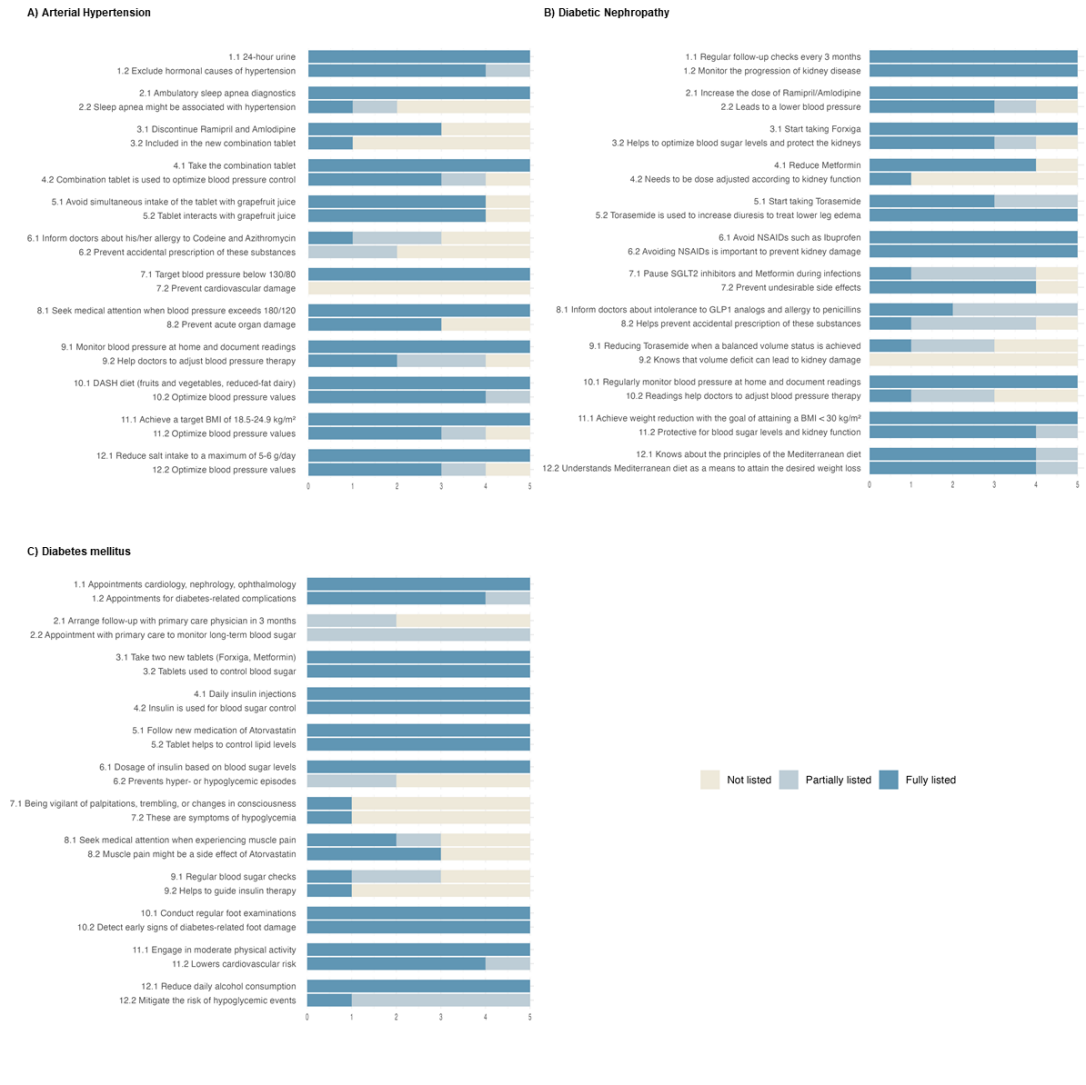
(A-C) Illustration of the learning objectives described in the discharge letters and their representation in the patient-centered letters. The colors indicate the extent to which each learning objective has been addressed in the patient-centered letters (dark blue: fully listed; light blue: partially listed; and yellow: not listed). The learning objectives are described on the y-axis and the number of patient letters per disease on the x-axis. GLP1: glucagon-like peptide-1; NSAID: nonsteroidal anti-inflammatory drug; SGLT2: sodium-glucose cotransporter 2;.

A detailed illustration of the learning objectives addressed in the patient-centered letters is provided in Figure 2. Out of the 72 learning objectives (24 per medical condition across 3 conditions), 57 were identified and addressed in the majority (≥3/5 letters per disease). However, no patient-centered letter included all the learning objectives present in the corresponding discharge letter. There was no significant difference in the coverage of learning objectives across the 3 diseases (AHT, DM, and DKD).

**Figure 3:**
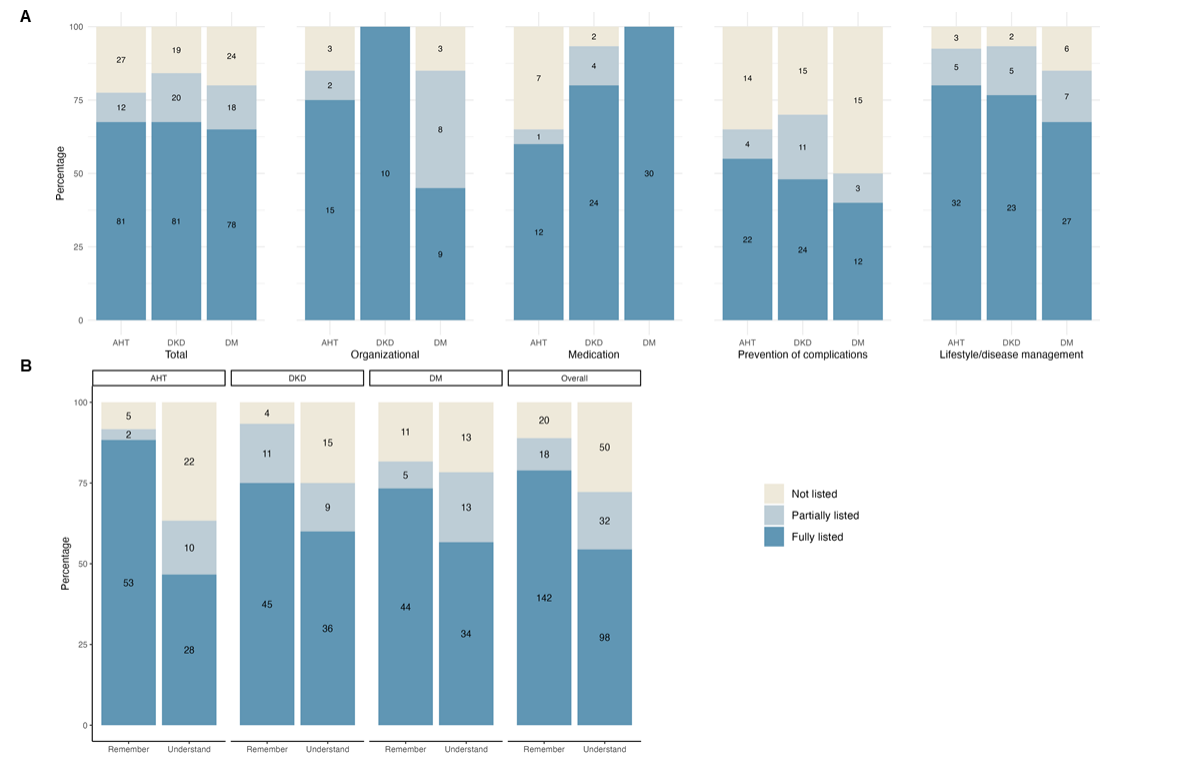
(A) Classification of learning objectives according to the relevant content field (organizational, medication, prevention of complications, and lifestyle). (B) Classification of learning objectives based on Bloom’s revised taxonomy (Remember, Understand). The colors indicate the extent to which the learning outcome is included in the patient letter (dark blue: fully included; light blue: partially included; and yellow: not included). AHT: arterial hypertension; DKD: diabetic kidney disease; DM: diabetes mellitus.

Figure 3 illustrates the learning objectives categorized according to Bloom’s taxonomy and content field. Notably, the figure reveals that learning objectives classified under Bloom’s “Remember” category were more frequently addressed in the patient-centered letters compared with those in the “Understand” category.

Some learning objectives were only partially addressed in the majority (≥3 of 5 letters) of the patient-centered letters (5/72). Examples of these partially addressed learning objectives are provided in Multimedia Appendix 5. The missing information in these cases could be categorized into different areas (eg, responsibility, frequency, dosage). However, no systematic categorical errors were identified in the analysis of the partially addressed learning objectives.

Of the 72 learning objectives, 11 were completely omitted in the majority (≥3 of 5 letters) of the patient-centered letters (Multimedia Appendix 4). Of these, only 2 belonged to the Remember category, while the majority were under the Understand category. Furthermore, most of the missing learning objectives (6/11) were related to the field of “prevention of complications.” By contrast, learning objectives related to “lifestyle” and “organizational” aspects were more frequently included in the patient-centered letters by GPT-4.

### Indicators of Patient Centricity

**Figure 4:**
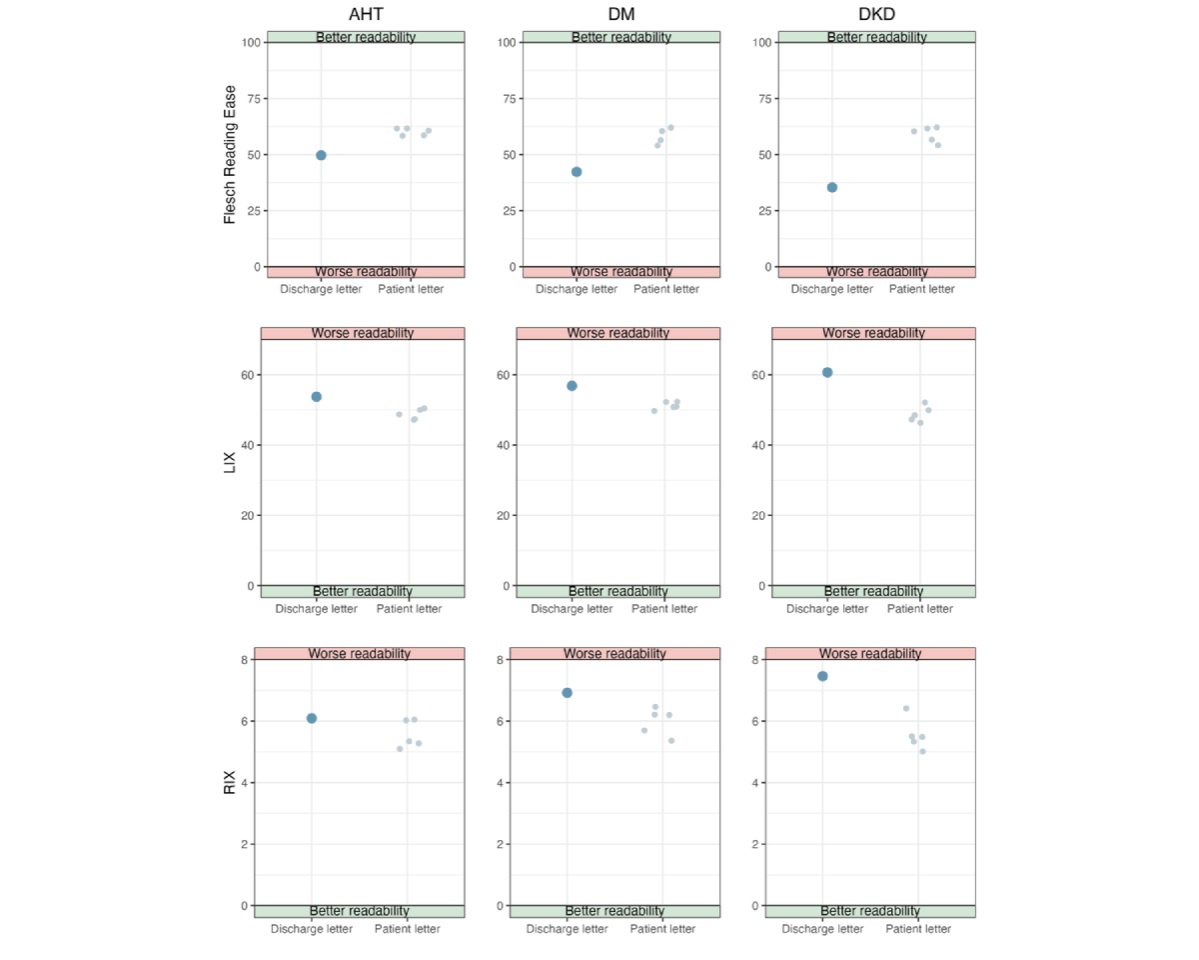
The graph shows the readability scores for the discharge letters and the patient-centered letters. In the Flesch Reading Ease test, a higher score indicates a better readability (green, good readability), whereas in the Läsbarhetsindex (LIX) and Readability Index (RIX), a higher score represents lower readability (red, worse readability). AHT: arterial hypertension; DKD: diabetic kidney disease; DM: diabetes mellitus.

The Flesch Reading Ease test scores for both the discharge letters and the patient-centered letters are shown in Figure 4. The patient-centered letters scored around 60, which corresponds to a “standard” reading level [26]. By contrast, the discharge letters scored around 40, indicating a “difficult” reading level. As illustrated in Figure 4, the patient-centered letters have an LIX score of approximately 50, while the discharge letters scored around 60, suggesting greater difficulty in reading. The patient-centered letters also scored lower than the discharge letters on the RIX, further demonstrating better readability.

Abbreviations were used in 49 of 273 (17.9%) discharge letter sentences, compared with 43 of 860 (5%) in the patient-centered letters. The abbreviation rate remained consistent across all patient letters (AHT: 14/273, 5.1%; DM: 14/273, 5.1%; and DKD: 15/273, 5.5%).

Repetitions were found in 37 of 273 (13.6%) discharge letter sentences and 207 of 860 (24.1%) patient-centered letter sentences. The number of repetitions varied slightly across the patient-centered letters (AHT: 60/278, 21.6%; DM: 75/271, 27.7%; and DKD: 72/311, 23.2%).

Medical terms appeared in 165 of 273 (60.4%) discharge letter sentences but in only 132 of 860 (15.3%) patient-centered letters. Of the medical terms used in patient-centered letters, 121 of 132 (91.7%) were explained.

Patients were directly addressed in 502 of 860 (58.4%) sentences in the patient-centered letters. This varied slightly between the different patient-centered letters (AHT: 151/278, 54.3%; DM: 149/271, 55%; DKD: 202/311, 65%).

### Medical Correctness, Case-Specific Relevance, and Source of Information

**Figure 5:**
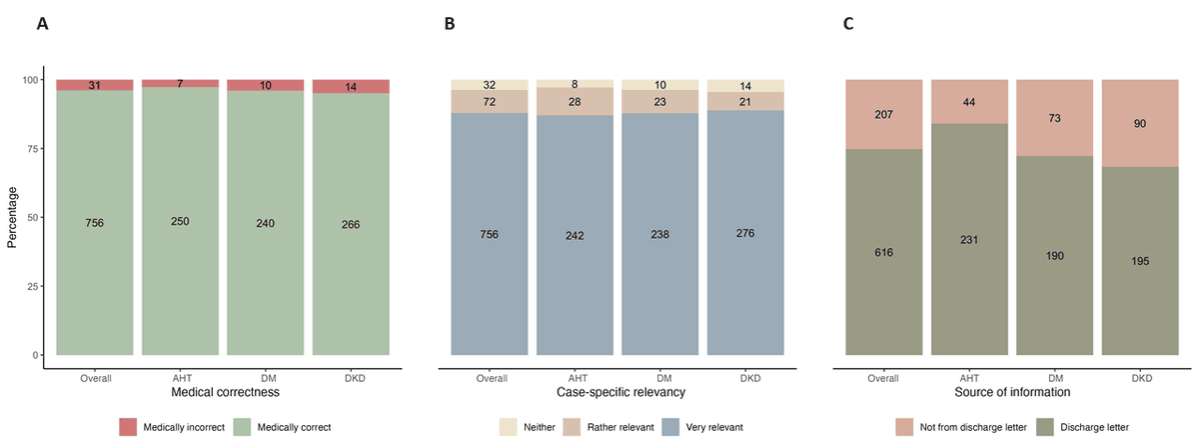
(A) Illustration of medically correct (green) and incorrect (red) sentences in the patient-centered letters with the absolute number of sentences displayed within the bars. (B) Representation of the case-specific relevance of the sentences in the patient-centered letters (blue: very relevant; brown: rather relevant; and yellow: neither/nor relevant). The absolute number of sentences is shown in the bars. (C) Proportion of sentences derived from the discharge letter (gray) and from GPT-4 (red) with the absolute number of sentences displayed within the bars. AHT: arterial hypertension; DKD: diabetic kidney disease; DM: diabetes mellitus.

Of the 860 patient letters, 756 sentences (87.9%) were medically correct, while 31 were medically incorrect. The rate of medically correct sentences was comparable among the patient letters for AHT (250/278, 89.9%), DM (240/271, 88.6%), and DKD (266/311, 85.5%). Medical errors were found in 7 of 278 (2.5%) sentences in the patient-centered letters on AHT, 10 of 271 (3.7%) letters on DM, and 14 of 311 (4.5%) letters on DKD (Figure 5).

Regarding case-specific relevance, 756 of 860 (87.9%) sentences in the patient-centered letters were very relevant, while 72 of 860 (8.4%) were rather relevant. By contrast, 32 of 860 (3.7%) sentences were not relevant. The proportion of highly relevant sentences was similar across the patient-centered letters on AHT, DM, and DKD (Figure 5).

The information in the patient-centered letters was derived from the discharge letters in 616 of 860 (71.6%) sentences. Additional medical information was provided by GPT-4 in 207 of 860 (24.1%) sentences. However, these proportions varied across the specific conditions. For instance, the patient-centered letters on AHT were based on the discharge letters in 231 of 278 (83.1%) sentences, compared with 190/271 (70.1%) for DM and 195/311 (62.7%) for DKD. Further details are shown in Figure 5.

A qualitative analysis of the medical errors, conducted using thematic analysis based on Braun and Clarke [25], can be found in Multimedia Appendix 6.

No discernible pattern was observed in the errors within the patient-centered letters. Some errors were due to imprecision or incomplete information, while others resulted from incorrect assumptions made by GPT-4. Overall, most of the sentences (182/204, 89.2%) generated by GPT-4 were medically correct. However, of the medically incorrect sentences, the majority (22/31, 71%) contained information provided solely by GPT-4.

## Discussion

### Principal Findings

This study demonstrates the potential of GPT-4 to transform discharge letters into more readable, patient-centered letters. While this aligns with previous studies that show GPT-4’s ability to generate patient-centered letters based on discharge letters or shorthand clinical instructions [16,27], our study goes beyond simply proving the concept of patient letter generation. We focused specifically on communicating relevant information from a patient-safety perspective. To our knowledge, this is the first study to analyze this aspect in AI-generated patient-centered letters.

### Automated Learning Objective Identification and Transformation of Treatment and Patient Safety–Relevant Information

Our primary focus was on identifying and transferring information relevant to patient safety, defined as “learning objectives,” in GPT-4–generated patient-centered letters. Overall, GPT-4 effectively identified and addressed 56 of the 72 learning objectives, with 5 partially addressed and 11 omitted entirely.

A key finding was that no significant differences were observed between the 3 diseases (AHT, DM, and DKD) in terms of the quality of GPT-4’s identification and transformation of learning objectives.

Another important finding is that GPT-4 fully identified and transformed 56 of the 72 (78%) learning objectives from the discharge letters. In 5 of 72 (7%) cases, GPT-4 identified relevant learning objectives related to treatment and patient safety but omitted certain aspects during the transformation into patient-centered letters. In 11 of 72 (15%) cases, GPT-4 failed to identify any learning objectives, and thus, did not transform or include them in the patient-centered letters.

Consistent with previous studies in the medical field, we observed that the omission of relevant key information in the medical context, as highlighted in AI research, remains a prevalent issue [16,27].

Given that the partial omission of relevant key information in the medical context is highly pertinent to the quality and safety of patient care, we have, to the best of our knowledge, examined this phenomenon in detail for the first time in this study.

From our perspective, it is particularly noteworthy that we observed more frequent omissions by GPT-4 in relation to more complex medical requirements. These omissions were primarily associated with learning objectives that demand a deeper understanding (Bloom’s category: Understand), as opposed to simpler objectives (Bloom’s category: Remember). Basic topics, such as “lifestyle” and “organizational” aspects of self-management (eg, regularly attending follow-up examinations every 3 months), were more often identified and effectively transformed into patient-friendly language by GPT-4. More complex content, such as “prevention of complications” (eg, interactions between certain foods and medications), was less frequently addressed by GPT-4. This phenomenon was particularly evident in the processing and transformation of complex logical structures involving multiple dependencies.

To address this challenge effectively, strategies such as “few-shot learning,” where the AI model is guided by several concise examples, and “chain-of-thought” prompts, which break down complex, multilevel problems into intermediate steps, could be promising. These approaches may enhance GPT-4’s ability to translate complex medical information into clear, easily understandable statements and actionable directions [28,29].

In our case, “chain-of-thought” prompting would need to begin with an initial step that clarifies to the AI why the patient should adhere to the respective learning objective. For example, the first step could involve identifying the content relevant to the patient (eg, “pay attention to interactions”). The next step would be for the AI to break this content down into individual components (eg, medication, diet, kidney function). In the final step, the AI should organize these components in the correct order. Only in the subsequent step should the AI check this order for existing dependencies between the individual information units and adjust it if necessary. This could help prevent the omission of complex learning objectives that are crucial for patient safety and quality of care. It should be noted that this approach extends the AI prompting, which may affect its performance in other areas. Therefore, this potentially promising strategy should be specifically explored in future studies to enhance the complete translation of complex medical information by GPT-4 into more easily understandable information for patients.

Regarding the learning objectives that were only partially transformed by GPT-4 from the discharge letters to the patient letters, we were unable to identify a clear connection with the content. Previous studies have demonstrated GPT-4’s ability to extract specific information from medical notes with a high degree of accuracy. However, in those studies, GPT-4 focused exclusively on the task of information extraction.

By contrast, our study involved a multitasking prompt that required both the identification of learning objectives and the subsequent transformation of these medical facts into the correct context and simple language for the creation of patient-centered letters. Similar to the findings related to the complete omission of complex learning objectives, the challenge for AI in processing and reproducing complex logical structures with multiple dependencies was also evident in this case.

Particularly challenging for GPT-4 appeared to be learning objectives from which no immediate or simple logical pattern could be derived. This may be attributed to the multitasking prompts (identification, summarization, and simplification of medical information) and the overall high complexity of medical issues when translated into accessible language for patients. An alternative approach for the prompt could involve implementing “action points” to clearly structure the learning objectives into required actions, success criteria, and potential consequences. This strategy warrants further investigation in future studies.

### Quality of Medical Information After Transformation by GPT-4

In this study, it was particularly important for us to assess the medical accuracy of the information transformed by GPT-4 in the patient-centered letters, as this is crucial for patient safety and quality of care. Any inaccuracies must therefore be regarded as a potential risk in the use of LLMs in a medical context.

Our results demonstrate that the patient-centered letters generated by GPT-4 exhibited a high degree of medical accuracy. The low error rate observed in our study may be attributed to the detailed medical information contained in the discharge letters, which GPT-4 accessed during the transformation process. This extensive data set enabled GPT-4 to accurately translate the medical information into patient-friendly language. Previous studies have shown that, in addition to information omissions, factors such as the type of task (eg, text summarization) and the specific field of application (eg, medical writing, question-answer format) influence the accuracy of LLM transformations [30]. Notably, our study demonstrates a significant improvement in medical accuracy and overall result quality compared with prior work examining various scenarios for the use of LLMs in the medical context [27,31].

A critical issue in transforming medical information using LLMs is the phenomenon of “hallucinations,” where coherent and grammatically correct, yet factually incorrect or misleading, information is generated [32,33]. These hallucinations can jeopardize patient safety and the quality of care. While previous studies have reported hallucination rates as high as 40%, our study encountered this phenomenon only in isolated cases [34]. Nevertheless, despite the observed downward trend, the risk remains significant with the ongoing development of LLMs, as the following example illustrates:

Discharge letter: *(...) An ultrasound of the pancreas showed no signs of a mass. (...)*

Patient-centered letter: *(...) they also tested to see if your pancreas has any issues, which it does not (...).*

Here, GPT-4 makes a general statement about the unremarkable ultrasound of the pancreas, despite the fact that medically, only a malignant disease was excluded, and other potential pathologies were not examined at all. While these types of errors, resulting from the hallucination phenomenon, occurred only very rarely, they still pose a significant risk to patient safety and the quality of care. Therefore, despite their low frequency, this phenomenon must be considered a substantial limitation to the use of LLMs in medical contexts and should be specifically addressed in future studies.

When using LLMs such as GPT-4 with sensitive medical information, ensuring compliance with data protection and information security requirements is paramount. Before deploying LLMs in health care settings, relevant experts must be consulted for each specific application scenario to define appropriate regulations for anonymizing medical information and to establish a secure and resilient infrastructure.

### Patient Centricity

In terms of readability and patient centricity—measured by standard readability scores, word and sentence counts, and the use of specialized terminology, explanations, abbreviations, and direct patient address—the GPT-4–generated patient-centered letters significantly outperformed the conventional discharge letters.

This is consistent with previous studies that analyzed AI-generated patient letters, although using less comprehensive methodologies, which showed high readability ranging from the sixth [16] to the ninth [27] grade level. It is important to note that these studies typically used the Flesch Reading Ease, the Flesch-Kincaid Reading Level, or the PEMAT score for readability assessments [16,35].

The Flesch Reading Ease is one of the most commonly used tools for evaluating readability in medical literature, with scores ranging from 0 (unreadable) to 100 (very easy to read) [22]. However, a major limitation of this approach is that it only considers sentence and word length, failing to adequately account for the level of medical knowledge required by patients or the frequency of medical terms used.

From the authors’ perspective, these factors, along with directly addressing patients, are crucial for how discharge letters are perceived and understood. Previous studies have shown that avoiding abbreviations and explaining medical terms in layperson’s terms significantly improve the comprehensibility of discharge letters from the patient’s viewpoint [36]. Additionally, medical letters are demonstrably better received by patients when they are personally addressed in the letter [37].

For this study, we implemented additional methodological approaches to analyze, for the first time, the patient-centered transformation of medical information by GPT-4. These advanced methods not only accounted for the use of medical terms and abbreviations but also included their explanations and the occurrence of repetitions.

It is noteworthy that the patient-centered letters transformed by GPT-4 demonstrated a significantly higher level of patient centricity than conventional discharge letters, even when accounting for these advanced parameters, which were examined for the first time. The GPT-4–generated letters contained notably fewer medical terms and abbreviations, explained medical terms in simple language, and directly addressed the patients.

### Limitations

Our study has several limitations. First, GPT-4’s generation of patient-centered letters was based solely on extensive prompting techniques. In the long term—especially with the increasing availability of additional LLMs [38,39]—fine-tuning (both general and domain-specific) and the potential application of specialized LLMs should be considered for the creation of patient-centered letters. Second, our investigation focused on major widespread diseases that occur frequently but did not encompass all medical specialties. As a result, our findings may not be fully transferable to all medical specialties. Another limitation is the exclusive use of the LLM “GPT-4,” which prevents us from drawing conclusions about the performance of other LLMs in generating patient-centered letters based on discharge letters.

Furthermore, we used a new multidimensional approach to assess the patient centricity of GPT-4–based communication of medical information. This approach considered factors such as the use of medical terms, abbreviations, and repetitions. While these variables are objectively measurable and part of common readability standards, they have not been sufficiently validated in the context of LLM-based communication. Therefore, further studies are needed to explicitly examine how patients perceive and understand the patient letters created by LLMs, as well as how this innovative form of patient-centered communication impacts patient empowerment and self-management of their health. Additionally, the perspective of the treating physicians on this promising form of patient-centered communication was not explored in our study. This aspect should also be considered in future research.

Additionally, the discharge letters, prompts, and patient letters were written in German, and all content was translated into English for the preparation of the manuscript. As a limitation, it should be noted that the transferability of our study results to the use of GPT in other languages requires further investigation.

### Conclusions

In summary, our study demonstrates that GPT-4 has the potential to significantly enhance the patient-centeredness of discharge letters. While we used a detailed prompting technique and GPT-4 generally exhibits a high degree of medical accuracy when transforming discharge letters into more patient-friendly formats, it is not yet fully suitable for patient care without review by medical professionals, particularly due to the noted omissions and hallucinations. Despite the already strong readability and patient orientation, even an advanced language model like GPT-4 did not fully account for all information relevant to patient safety and quality of care in the patient-centered letters. Further advancements in prompting techniques and the targeted development of language models for the medical field could help minimize these limitations. If these challenges are addressed, GPT-4 could offer enhanced potential to support health care professionals in patient-centered communication and improve patients’ understanding of their medical conditions. This would mark a significant step toward better patient safety and improved quality of care.

## Appendix

Multimedia Appendix 1 Discharge letters, original prompting, examples of patient letters, and table with learning objectives.

Multimedia Appendix 2 Illustration of the multistep process used for the development of discharge letters. Three common diseases were selected as the basis for the discharge letters. To assess GPT-4's ability to identify critical medical information, 24 learning objectives were developed in alignment with Bloom’s taxonomy. Examples are given in the figure.

Multimedia Appendix 3 Overview of the multistep process of GPT-4 prompting. The 3 tasks involved in the generation of the patient-centered letters are illustrated in the rectangles. The direction of the arrows indicates the sequential order of the individual steps.

Multimedia Appendix 4 Qualitative analysis of missing learning objectives. This table illustrates learning objectives that were not included in the majority of patient-centered letters (≥3/5 letters). Recommendations are provided on how the prompting can be refined to address the missing information.

Multimedia Appendix 5 Analysis of incomplete learning objectives. The table presents examples of learning objectives that have been partially listed in the patient-centered letters including recommendations for further prompt engineering.

Multimedia Appendix 6 Qualitative analysis of medical errors. Medical errors made by GPT-4 were categorized and analyzed based on content area. The errors were classified into different error categories: imprecise, incorrect, incomplete, and presumptive.

## Abbreviations

AHT: arterial hypertension
AI: artificial intelligence
DKD: diabetic kidney disease
DM: diabetes mellitus
LIX: Läsbarhetsindex
LLM: large language model
PEMAT: Patient Education Materials Assessment Tool
RIX: Readability Index
WHO: World Health Organization
