# Supplementary material for: What’s Going On With Me and How Can I Better Manage My Health? The Potential of GPT-4 to Transform Discharge Letters Into Patient-Centered Letters to Enhance Patient Safety: Prospective, Exploratory Study
Source: J Med Internet Res. 2025 Jan 21;27:e67143. doi: 10.2196/67143 (PMC11795158; doi:10.2196/67143)
Supplement: Multimedia Appendix 1 [file jmir_v27i1e67143_app1.docx]

|  |  | |
| --- | --- | --- |
|  | | **University hospital**  Department of Internal Medicine IV  Diabetology  Endocrinology  Nephrology  Medical Director  Prof. Konrad Kaiser, MD  Otfried-Müller-Strasse 10  72076 Tübingen  Service telephone: 07071 29- 123456  Fax:  07071 29- 23456    Contact person:  Mrs. Erika Eckstei, MD |
| University hospital ▪ Otfried-Müller-Str. 10 ▪ 72076 Tübingen | |  |
| Mr.  Anton Arzt, MD  General Practitioner  Arztweg 2  72070 Tübingen | |  |
|  | |  |
|  | |  |

**Supplementary Material 1: Discharge letters (English version/ German version)**

**a) Arterial Hypertension**

Patient: **Ronny Racer,** date of birth: **12.07.1983** Patient case no. 1 **23456789**

Address: **Mercedesstraße 1,** **72074 Tübingen**

**Nephrology outpatient clinic:** Outpatient presentation at **04.10.2023** Please have ready in case of queries

Dear colleague,

We report on the presentation of the above-mentioned patient in our Nephrology Outpatient Clinic.

**Diagnosis:**

| 1. | **Arterial hypertension grade II, initial diagnosis 03/2023, most probably essential arterial hypertension**   - Secondary cause clarification:   - Sleep apnea syndrome: pending   - No evidence of renal artery stenosis (MRI from 16.8.2023, University Hospital Tübingen)   - Inconspicuous aldosterone-renin quotient   - Cortisol not elevated in the urine sample   - Collection urine for metanephrine: pending   - TSH in normal range, thyroid sonography: unremarkable thyroid gland   - Laboratory-chemically unremarkable kidney function - Hypertensive end organ damage (HMOD screening): Hypertensive nephropathy: albuminuria 64 mg/g creatinine - CVRF: positive family history of stroke in the father at the age of approx. 50-60 years, no other risk factors in the medical history, SCORE2 risk 3% |
| --- | --- |
| 2. | Status post reflux esophagitis |
| 5. | Status post right arthroscopy |
| 6. | Hypercholesterolemia |
| 7. | Allergy to codeine and azithromycin |

**Current therapy recommendation:**

| Ramipril/amlodipine/HCT 10/10/25 mg | 1 - 0 - 0, **NEW** |
| --- | --- |
| Ramipril 5 mg | 1 - 0 - 1, **FINISHED** |
| Amlodipine 10 mg | 1 - 0 - 0, **FINISHED** |
| Pantoprazole 20 mg | 1 - 0 - 0 |

**Further procedure:**

- Change of antihypertensive therapy as indicated above.
- We ask you to perform a 24-hour acidified urine collection to determine the metanephrines in the urine. A container for this has been provided.

**Laboratory/urinalysis:**

See table appendix

**Sonography:**

Right kidney: Easy to assess, orthotopic position, normal size, normal shape, parenchymal margin age-appropriate, regular medullary/cortical differentiation, smooth organ contour, renal pelvic caliceal system not dilated, no concrements, RIs average 0.52

Left kidney: Easy to assess, orthotopic position, normal size, connective tissue retraction at the upper pole, otherwise normal shape, parenchymal margin age-appropriate, regular medullary/cortical differentiation, smooth organ contour, renal pelvic caliceal system not dilated, no concrements, RIs average 0.54

Adrenal glands: Normal size, no focal lesions, adrenal glands free on both sides

Urinary bladder: limited assessment after micturition, orthotopic location, no pathologic echostructures intraluminally

Ureter: not dilated

Pleura: no pleural effusion on both sides

Peritoneum/ ascites: No ascites. No evidence of free fluid

Assessment of the inferior vena cava: Cava inf. with approx. 1.4 cm normal width and respiratory modulation.

Summarized assessment

Sonographic examination shows no evidence of secondary hypertension.

**Summarized assessment:**

The elective presentation of R. Racer in our nephrology outpatient clinic was for assessment and clarification of secondary causes of arterial hypertension.

R. Racer reports that in March of this year, as part of the investigation into the consequences of a car accident, repeatedly elevated blood pressure values of 170-180 mmHg systolic and 130 mmHg diastolic were noticed. As a result, treatment with ramipril and amlodipine was initiated. Since then, the blood pressure values have been around 150 mmHg systolic. In addition, the patient is in good general health. Apart from the previous illnesses listed above, no other illnesses were known. With regard to the family history, R. Racer reported that his father had suffered a stroke between the ages of 50 and 60. He sleeps 6-8 hours a day, his sleep is not very restful and he is known to snore. His daily caffeine consumption amounted to 2 cups of coffee per day. R. Racer states that he occasionally does endurance sports (running). No nicotine or other substance abuse. The daily drinking volume is about 2 liters/day. Micturition was unremarkable. The blood pressure after starting therapy is now in the range between 150-160 mmHg systolic and around 90 mmHg diastolic in the blood pressure measurements taken at home.

Clinically, R. Racer was in a good general condition and in a pre-adipose nutritional state, weight 90 kg, height 1.74 m, BMI 29.72 kg/m². Blood pressure in the right upper arm 155/95 mmHg, left 153/97 mmHg, heart rate 76/min, rhythmic. No high-grade peripheral edema. Sonographic examination shows no evidence of a secondary genesis of hypertension.

In the laboratory analysis, the creatinine is 0.8 mg/dl, which is in the normal range, resulting in an eGFR (CKD-EPI) of 88 ml/min/1.73 m². There is proteinuria of 164 mg/g creatinine with albuminuria of 63.6 mg/g creatinine. The LDL cholesterol was found to be 153 mg/dl, but primary prophylaxis with a statin was dispensed due to the lack of other risk factors.

To further clarify the cause of the hypertension, a 24-hour urine sample was taken for cortisol, the aldosterone-renin quotient was determined and the thyroid hormones were checked. These diagnostic tests were all negative. A relevant renal artery stenosis had already been ruled out in the abdominal MRI 08/2023. We request the addition of a 24h urine collection for metanephrine to clarify a pheochromocytoma. In addition, a sleep apnea syndrome should be excluded in the outpatient setting.

In summary of the findings, we currently assume essential hypertension. The blood pressure values are currently above the target range under antihypertensive therapy, so we recommend a change in therapy as stated above (CAVE: avoid simultaneous intake with CYP inhibitors such as grapefruit juice). In addition, regular blood pressure self-checks with written documentation should be carried out at home to control therapy. Hypertensive derailments (RR > 180/120 mmHg) require immediate medical consultation, as otherwise there is a risk of end organ damage. To avoid cardiovascular complications, a target blood pressure below 130/80 mmHg should be aimed for in everyday life.

In addition to the drug therapy described above, we also recommend making full use of conservative, antihypertensive measures. These include a change in diet in line with a DASH diet (plenty of fruit and vegetables, low-fat dairy products), weight reduction to a target BMI of 18.5-24.9 kg/m² and a reduced salt intake of max. 5-6 g/day.

We kindly ask you to follow the above procedure in the context of further outpatient care by a general practitioner.

If you have any questions, please do not hesitate to contact us.

With best regards

This letter was typed and therefore does not bear a signature.

| Prof. Anton Arnold, MD  Head of Section  Kidney and hypertension diseases | Tina Torpedo, MD  Senior physician in nephrology, hypertensiologist DHL | Erika Eckstein, MD  Resident |
| --- | --- | --- |

**Laboratory**

| Destination | 06.10.23 | 05.10.23 | | 04.10.23 | | 23.03.22 | | 22.03.22 | | 22.03.22 | | 21.03.23 | | Unit | Reference |
| --- | --- | --- | --- | --- | --- | --- | --- | --- | --- | --- | --- | --- | --- | --- | --- |
| Order | 11:32 | 10:48 | | 10:35 | | 07:00 | | 11:26 | | 08:24 | | 21:05 | | | |
| Order number |  | |  | |  | |  | |  | |  | |  | | |
| **(Diff) blood count: EDTA blood** | | | | | | | | | | | | | | | |
| Leukocytes |  |  | | 5990 | | 5540 | |  | |  | |  | | 1/µl | 4100 - 11800 |
| Neutrophils abs |  |  | | 3.80 | |  | |  | |  | |  | | Thousand/µl | 2.1 - 7.7 |
| Lymphocytes abs |  |  | | 1.71 | |  | |  | |  | |  | | Thousand/µl | 1.2 - 3.5 |
| Monocytes abs |  |  | | 0.37 | |  | |  | |  | |  | | Thousand/µl | 0.25 - 0.85 |
| Eosinophil abs |  |  | | 0.06 | |  | |  | |  | |  | | Thousand/µl | 0.03 - 0.47 |
| Basophilic abs |  |  | | 0.04 | |  | |  | |  | |  | | Thousand/µl | 0.02 - 0.11 |
| Immature granulocytes abs |  |  | | 0.01 | |  | |  | |  | |  | | Thousand/µl | 0 - 0.06 |
| Neutrophils |  |  | | 63.4 | |  | |  | |  | |  | | % | 40.0 - 80.0 |
| Lymphocytes |  |  | | 28.5 | |  | |  | |  | |  | | % | 20.0 - 45.0 |
| Monocytes |  |  | | 6.2 | |  | |  | |  | |  | | % | 2.0 - 13 |
| Eosinophils |  |  | | 1.0 | |  | |  | |  | |  | | % | 0.5 - 8 |
| Basophile |  |  | | 0.7 | |  | |  | |  | |  | | % | max. 2.0 |
| Immature granulocytes |  |  | | 0.2 | |  | |  | |  | |  | | % | 0 - 0.6 |
| Normoblasts abs |  |  | | 0 | | 0 | |  | |  | |  | | Thousand/µl | 0 - 0.1 |
| Normoblasts |  |  | | 0 | | 0 | |  | |  | |  | | % | 0 - 1 |
|  | | | | | | | | | | | | | | | |
| Erythrocytes |  |  | | 4.86 | | 4.51 | |  | |  | |  | | Mio/µl | 4.2 - 6.2 |
| Haematocrit |  |  | | 41.5 | | 38.5 | |  | |  | |  | | % | 37.0 - 47.0 |
| HB |  |  | | 14.5 | | 13.4 | |  | |  | |  | | g/dl | 12.0 - 16.0 |
| MCH |  |  | | 29.8 | | 29.7 | |  | |  | |  | | pg | 27.0 - 34.0 |
| MCHC |  |  | | 34.9 | | 34.8 | |  | |  | |  | | g/dl | 32.0 - 36 |
| MCV |  |  | | 85.4 | | 85.4 | |  | |  | |  | | fl | 80 - 93 |
| Ery. Distribution width (RDW) |  |  | | 12.2 | | 12.1 | |  | |  | |  | | % | max. 15 |
| Thrombocytes |  |  | | 244 | | 193 | |  | |  | |  | | Thousand/µl | 150 - 450 |
| Distribution range of the platelets |  |  | | 11.9 | | 12.4 | |  | |  | |  | | fl | 10 - 16 |
| Medium platelet volume |  |  | | 10.3 | | 11.0 | |  | |  | |  | | fl | 9 - 12 |
| **Coagulation: citrated blood** | | | | | | | | | | | | | | | |
| Quick |  |  | | 115 | |  | |  | |  | |  | | % | 70 - 120 |
| INR |  |  | | 0.9 | |  | |  | |  | |  | |  |  |
| aPTT |  |  | | 24 | |  | |  | |  | |  | | sec | max. 40 |
| **Electrolytes: Li-heparin plasma** | | | | | | | | | | | | | | | |
| Sodium |  |  | | 141 | | 139 | |  | |  | |  | | mmol/l | 136 - 148 |
| Potassium |  |  | | 4.3 | | 3.8 | |  | |  | |  | | mmol/l | 3.5 - 4.8 |
| Calcium |  |  | | 2.2 | | 2.2 | |  | |  | | 2.3 | | mmol/l | 2.1 - 2.6 |
| Inorganic phosphate |  |  | | 0.9 | |  | |  | |  | |  | | mmol/l | 0.8 - 1.5 |
| **Substrates: Li-heparin plasma** | | | | | | | | | | | | | | | |
| Creatinine |  |  | | 0.8 | | 0.7 | |  | |  | | 0.8 | | mg/dl | 0.5 - 0.8 |
| GFR - CKD-EPI |  |  | | 88 | |  | |  | |  | | 88 | | ml/min/ 1,73m2 |  |
| Urea |  |  | | 36 | |  | |  | |  | | 23 | | mg/dl | 12 - 46 |
| Uric acid |  |  | | 5.7 | |  | |  | |  | |  | | mg/dl | 2.4 - 5.7 |
| Bilirubin, total |  |  | |  | |  | |  | |  | | 0.6 | | mg/dl | max. 1.1 |
| Total egg white |  |  | | 7.3 | |  | |  | |  | |  | | g/dl | 6.5 - 8.5 |
| Cholesterol |  |  | | 218 | |  | |  | |  | |  | | mg/dl | 130 - 190 |
| HDL cholesterol |  |  | | 65 | |  | |  | |  | |  | | mg/dl | min. 45 |
| LDL cholesterol |  |  | | 153 | |  | |  | |  | |  | | mg/dl | max. 160 |
| Triglycerides |  |  | | 65 | |  | |  | |  | |  | | mg/dl | max. 200 |
| C-reactive protein (CRP) |  |  | | 0.43 | | 0.33 | |  | |  | | 0.64 | | mg/dl | max. 0.50 |
| **Enzymes: Li-heparin plasma** | | | | | | | | | | | | | | | |
| Creatine kinase (CK) |  |  | | 87 | | 49 | |  | |  | | 99 | | U/l | max. 170 |
| GOT/AST |  |  | |  | |  | |  | |  | | 9 | | U/l | max. 35 |
| GPT/ALT |  |  | | 13 | |  | |  | |  | | 15 | | U/l | max. 34 |
| Alkaline phosphatase (AP) |  |  | |  | |  | |  | |  | | 76 | | U/l | 35 - 105 |
| Lactate dehydrogenase (LDH) |  |  | | 205 | |  | |  | |  | | 212 | | U/l | max. 250 |
| Gamma-glutamyl transferase (GGT) |  |  | | 18 | |  | |  | |  | | 16 | | U/l | max. 40 |
| Lipase |  |  | |  | |  | |  | |  | | 39 | | U/l | max. 60 |
| **Hormones: Li-heparin plasma** | | | | | | | | | | | | | | | |
| TSH |  |  | |  | |  | |  | |  | | 3.01 | | mU/l | 0.5 - 4.4 |
| **Hormones: EDTA plasma** | | | | | | | | | | | | | | | |
| Renin after orthostasis |  |  | | 33 | |  | |  | |  | |  | | ng/l | 7 - 42 |
| Aldosterone after orthostasis |  |  | | 146 | |  | |  | |  | |  | | ng/l | 13 - 230 |
| **Hormones: Serum** | | | | | | | | | | | | | | | |
| TSH |  |  | | 2.06 | |  | |  | |  | |  | | mU/l | 0.5 - 4.4 |
| **Lactate, glucose: Na-F plasma** | | | | | | | | | | | | | | | |
| Lactate, photometric |  |  | |  | |  | |  | |  | | 0.8 | | mmol/l | 0.5 - 2.2 |
| Venous glucose |  |  | |  | |  | |  | |  | | 85 | | mg/dl | 70 - 99 |
| **Other: EDTA blood** | | | | | | | | | | | | | | | |
| HbA1c (IFCC) |  |  | | 33.0 | |  | |  | |  | |  | | mmol/mol | 26 - 44 |
| HbA1c (glycated hemoglobin) |  |  | | 5.2 | |  | |  | |  | |  | | % | 4.5 - 6.2 |
| **Urine status: Spontaneous urine** | | | | | | | | | | | | | | | |
| Urine status |  |  | | Urine status | |  | |  | |  | |  | |  |  |
| Color of the spontaneous urine |  |  | | Light yellow | |  | |  | |  | |  | |  |  |
| Turbidity in spontaneous urine |  |  | | clear | |  | |  | |  | |  | |  |  |
| Specific gravity in spontaneous urine |  |  | | 1.009 | |  | |  | |  | |  | | g/cmE3 |  |
| pH value in spontaneous urine |  |  | | 7.0 | |  | |  | |  | |  | |  |  |
| Protein quality in spontaneous urine |  |  | | N. | |  | |  | |  | |  | |  |  |
| Gluc.qual. in spontaneous urine |  |  | | N. | |  | |  | |  | |  | |  |  |
| U-ketone in spontaneous urine |  |  | | N. | |  | |  | |  | |  | |  |  |
| Hb qual. in spontaneous urine |  |  | | N. | |  | |  | |  | |  | |  |  |
| Urobilinogen in spontaneous urine |  |  | | N. | |  | |  | |  | |  | |  |  |
| Bilirubin in spontaneous urine |  |  | | N. | |  | |  | |  | |  | |  |  |
| Leukocytes in spontaneous urine |  |  | | N. | |  | |  | |  | |  | |  |  |
| Nitrite in spontaneous urine |  |  | | N. | |  | |  | |  | |  | |  |  |
| **Urine sediment (automated, digital microscopy)** | | | | | | | | | | | | | | | |
| Erythrocytes |  |  | | 5 | |  | |  | |  | |  | | 1/µl | 0 - 7 |
| Leukocytes |  |  | | 3 | |  | |  | |  | |  | | 1/µl | 0 - 9 |
| Bacteria |  |  | | (+) | |  | |  | |  | |  | |  | max. negative |
| Squamous epithelium |  |  | | (+) | |  | |  | |  | |  | |  | max (+) |
| Non-squamous epithelia |  |  | | (+) | |  | |  | |  | |  | |  | max (+) |
| **Other: Spontaneous urine** | | | | | | | | | | | | | | | |
| Creatinine conc. in spontaneous urine |  |  | | 55 | |  | |  | |  | |  | | mg/dl |  |
| Protein conc. in spontaneous urine |  |  | | 0.09 | |  | |  | |  | |  | | g/l | max. 0.10 |
| Quot. U-Eiw / U-Krea (spontaneous urine) |  |  | | 164 | |  | |  | |  | |  | | mg/g Crea | max. 100 |
| Quot. U-Eiw/U-Krea (spontaneous urine) |  |  | | 18.49 | |  | |  | |  | |  | | mg/mmol Crea | max. 11.3 |
| Albumin concentration |  |  | | 35 | |  | |  | |  | |  | | mg/l | max. 20 |
| Quot. U-albumin /g Crea |  |  | | 63.6 | |  | |  | |  | |  | | mg/g Crea | max. 20 |
| A1 microglobulin concentration |  |  | | <5 | |  | |  | |  | |  | | mg/l | max. 13 |
| A2 macroglobulin concentration |  |  | | <2.5 | |  | |  | |  | |  | | mg/l | 0 - <2.4 |
| IgG concentration in spontaneous urine |  |  | | 4 | |  | |  | |  | |  | | mg/l | max. 10 |
| U-IgG / albumin quotient in spontaneous urine |  |  | | 0.1 | |  | |  | |  | |  | |  | max. 0.2 |
| IgG /g Crea |  |  | | 7.3 | |  | |  | |  | |  | | mg/g Crea | max. 10 |
|  | | | | | | | | | | | | | | | |
| Total metanephrines in collected urine | 254 |  | |  | |  | |  | |  | |  | | µg/24h | < 900 |
| Free cortisol in the urine sample |  | 38 | |  | |  | |  | |  | |  | | µg/24h | 4,2 - 60 |

|  |  | |
| --- | --- | --- |
|  | | **Medizinische Klinik** Abteilung Innere Medizin IV  Diabetologie  Endokrinologie  Nephrologie  Ärztlicher Direktor  Prof. Dr. med. Konrad Kaiser  Otfried-Müller-Straße 10  72076 Tübingen  Servicetelefon: 07071 29-123456  Fax: 07071 29-23456  Ansprechpartner:  Frau Dr. Erika Einstein |
| Medizinische Klinik ▪ Otfried-Müller-Str. 10 ▪ 72076 Tübingen | |  |
| Herrn  Dr. med. Anton Arzt  FA für Allgemeinmedizin  Arztweg 2  72070 Tübingen | |  |
|  | |  |
|  | |  |

Patient: **Ronny Raser,** Geb.-Datum: **12.07.1983** Patienten-Fall-Nr. **123456789**

wohnhaft: **Mercedesstraße 1, 72074 Tübingen**

**Nephrologische Ambulanz:** Ambulante Vorstellung am **04.10.2023** bitte bei Rückfragen bereithalten

Sehr geehrter Herr Kollege,

wir berichten über die Vorstellung des oben genannten Patienten in unserer Nephrologischen Ambulanz.

**Diagnose:**

| 1. | **Arterielle Hypertonie Grad II ED 03/2023, a.e. essentielle arterielle Hypertonie**   - Sekundäre Ursachenabklärung:   - Schlafapnoesyndrom: ausstehend   - Kein Hinweis auf eine Nierenarterienstenose (MRT vom 16.8.2023 UK Tübingen)   - Unauffälliger Aldosteron-Renin-Quotient   - Cortisol im Sammelurin nicht erhöht   - Sammelurin auf Metanephrine: ausstehend   - TSH im Normbereich, sonografisch unauffällige Schilddrüse   - Laborchemisch unauffällige Nierenfunktion - Hypertensive Endorganschädigung (HMOD-Screening): V.a. hypertensive Nephropathie: Albuminurie 64 mg/g Kreatinin - CVRF: pos. Familienanamnese bei Stroke des Vaters im Alter von ca. 50-60 Jahren, anamnestisch keine weiteren Risikofaktoren, SCORE2 Risiko 3% |
| --- | --- |
| 2. | Z.n. Reflux-Ösophagitis |
| 5. | Z.n. Arthroskopie rechts |
| 6. | Hypercholesterinämie |
| 7. | Allergie gegen Codein und Azithromycin |

**Aktuelle Therapieempfehlung:**

| Ramipril/Amlodipin/HCT 10/10/25 mg | 1 – 0 – 0, **NEU** |
| --- | --- |
| Ramipril 5 mg | 1 – 0 – 1, **BEENDET** |
| Amlodipin 10 mg | 1 – 0 – 0, **BEENDET** |
| Pantoprazol 20 mg | 1 – 0 – 0 |

**Weiteres Procedere:**

- Umstellung der antihypertensiven Therapie wie oben angegeben
- Wir bitten um Durchführung eines 24h angesäuerten Sammelurins zur Bestimmung der Metanephrine im Urin. Ein Behälter hierfür wurde dem Patienten mitgegeben.

**Labor/Urinanalytik:**

Siehe Tabellenanhang

**Sonographie:**

Rechte Niere: Gut beurteilbar, orthotope Lage, normal groß, normale Form, Parenchymsaum altersentsprechend, regelrechte Mark-/Rindendifferenzierung, glatte Organkontur, Nierenbeckenkelchsystem nicht erweitert, keine Konkremente, RIs durchschnittlich 0,52

Linke Niere: Gut beurteilbar, orthotope Lage, normal groß, bindegewebige Einziehung am Oberpol sonst normale Form, Parenchymsaum altersentsprechend, regelrechte Mark-/Rindendifferenzierung, glatte Organkontur, Nierenbeckenkelchsystem nicht erweitert, keine Konkremente, RIs durchschnittlich 0,54

Nebennieren: Normal groß, keine fokalen Läsionen, Nebennierenlogen beidseits frei

Harnblase: eingeschränkt beurteilbar nach Miktion, orthotop gelegen, intraluminal keine pathologischen Echostrukturen

Ureter: Nicht erweitert darstellbar.

Pleura: Beidseits kein Pleuraerguss

Peritoneum/ Aszites: Kein Aszites. kein Nachweis freier Flüssigkeit

Vena Cava inferior Beurteilung: Cava inf. mit ca. 1,4 cm normal weit und atemmoduliert.

Zusammenfassende Beurteilung

Sonomorphologisch kein Hinweis für sekundäre Hypertonie.

**Zusammenfassende Beurteilung:**

Die elektive Vorstellung von Herrn Raser in unserer nephrologischen Ambulanz erfolgte zur Mitbeurteilung und Abklärung sekundärer Ursachen einer arteriellen Hypertonie.

Herr Raser berichtet, dass im März dieses Jahrs im Rahmen der Abklärung von Unfallfolgen eines Autounfalls wiederholt erhöhte Blutdruckwerte um 170-180 mmHg systolisch sowie um 130 mmHg diastolisch aufgefallen seien. Hieraufhin sei eine Therapie mit Ramipril und Amlodipin eingeleitet worden. Die Blutdruckwerte seien seitdem um 150 mmHg systolisch. Darüber hinaus besteht ein ordentliches Allgemeinbefinden. Es seien neben oben aufgeführten Vorerkrankungen keine weiteren Erkrankungen bekannt. Hinsichtlich der Familienanamnese berichtet Herr Raser über einen Schlaganfall des Vaters im Alter zwischen 50 und 60 Jahren. Beruflich sei er als Bankkaufmann tätig. Er schlafe täglich 6-8 Stunden, der Schlaf sei wenig erholsam, Schnarchen sei bekannt. Der tägliche Koffeinkonsum belaufe sich auf 2 Tassen Kaffee pro Tag. Herr Raser gibt an, gelegentlich Ausdauersport (Laufen) zu betreiben. Kein Nikotin- oder sonstige Substanzabusus. Die tägliche Trinkmenge betrage etwa 2 l/Tag. Die Miktion sei unauffällig. Der Blutdruck nach begonnener Therapie liege nun in den häuslich durchgeführten Blutdruckmessungen im Bereich zwischen 150-160 mmHg systolisch sowie um 90 mmHg diastolisch.

Klinisch zeigt sich der Patient in einem guten Allgemeinzustand sowie präadipösem Ernährungszustand, Gewicht 90 kg, Größe 1,74 m, BMI 29,72 kg/m². Blutdruck im Bereich des rechten Oberarmes 155/95 mmHg, links 153/97 mmHg, Herzfrequenz 76/min, rhythmisch. Keine höhergradigen peripheren Ödeme. Sonografisch ergab sich kein Hinweis für eine sekundäre Genese der Hypertonie.

Laborchemisch zeigt sich das Kreatinin mit 0,8 mg/dl im Normbereich, hieraus ergibt sich eine eGFR (CKD-EPI) von 88 ml/min/1,73 m². Es besteht eine Proteinurie von 164 mg/g Kreatinin bei einer Albuminurie von 63,6 mg/g Kreatinin. Nebenbefundlich zeigt sich das LDL-Cholesterin bei 153 mg/dl, auf eine Primärprophylaxe mit einem Statin wird allerdings aufgrund fehlender sonstiger Risikofaktoren verzichtet.

Zur weiteren Ursachenabklärung der Hypertonie erfolgte die Abnahme eines Sammelurins auf Cortisol über 24 Stunden, die Bestimmung des Aldosteron-Renin-Quotienten sowie die Kontrolle der Schilddrüsenhormone. Diese Diagnostik zeigte sich allesamt negativ. Im MR des Abdomens 08/2023 hatte bereits eine relevante Nierenarterienstenose ausgeschlossen werden können. Wir bitten um Ergänzung eines 24h Sammelurins auf Metanephrine zur Abklärung eines Phäochromozytoms. Zudem sollte im ambulanten Rahmen ein Schlafapnoesyndrom ausgeschlossen werden.

In Zusammenschau der Befunde gehen wir aktuell von einer essentiellen Hypertonie aus. Die Blutdruckwerte befinden sich unter der antihypertensiven Therapie aktuell über dem Zielbereich, sodass wir eine Therapieumstellung wie oben angegeben empfehlen (CAVE: gleichzeitige Einnahme mit CYP Inhibitoren wie Grapefruitsaft vermeiden). Zudem sollten zur Therapiesteuerung im häuslichen Rahmen regelmäßige Blutdruckselbstkontrollen mit schriftlicher Dokumentation erfolgen. Hypertensive Entgleisungen (RR > 180/120 mmHg) erfordern eine umgehende ärztliche Rücksprache, da sonst Endorganschäden drohen. Zur Vermeidung kardiovaskulärer Komplikationen ist im Alltag ein Zielblutdruck unter 130/80 mmHg anzustreben.

Zudem empfehlen wir neben der oben beschriebenen medikamentösen Therapie das Ausschöpfen der konservativen, antihypertensiven Maßnahmen. Hierzu gehört die Ernährungsumstellung im Sinne einer DASH Diät (reichlich Obst und Gemüse, fettreduzierte Milchprodukte), Gewichtsreduktion auf einen Ziel BMI von 18,5-24,9 kg/m² sowie eine reduzierte Kochsalzzufuhr von max. 5-6 g/Tag.

Wir bitten um Beachtung des obenstehenden Procederes im Rahmen der weiteren ambulanten hausärztlichen Betreuung.

Bei Rückfragen stehen wir Ihnen gerne zur Verfügung.

Mit freundlichen kollegialen Grüßen

Dieser Brief wurde maschinell erstellt und trägt daher keine Unterschrift.

| Prof. Dr. med. Anton Arnold  Leiter Sektion  Nieren- und Hochdruckkrankheiten | Dr. med. Tina Torpedo  Oberärztin Nephrologie, Hypertensiologin DHL | Dr. med. Erika Einstein  Assistenzärztin |
| --- | --- | --- |

**Labor**

| Bestimmung | 06.10.23 | 05.10.23 | | 04.10.23 | | 23.03.22 | | 22.03.22 | | 22.03.22 | | 21.03.23 | | Einheit | Referenz |
| --- | --- | --- | --- | --- | --- | --- | --- | --- | --- | --- | --- | --- | --- | --- | --- |
| Auftrag | 11:32 | 10:48 | | 10:35 | | 07:00 | | 11:26 | | 08:24 | | 21:05 | | | |
| Auftrag-Nummer | 595432 | | 595432 | | 40415 | | 40034 | | 68173 | | 72868 | | 43615 | | |
| **(Diff)-Blutbild: EDTA-Blut** | | | | | | | | | | | | | | | |
| Leukozyten |  |  | | 5990 | | 5540 | |  | |  | |  | | 1/µl | 4100 - 11800 |
| Neutrophile abs |  |  | | 3.80 | |  | |  | |  | |  | | Tausd/µl | 2.1 - 7.7 |
| Lymphozyten abs |  |  | | 1.71 | |  | |  | |  | |  | | Tausd/µl | 1.2 - 3.5 |
| Monozyten abs |  |  | | 0.37 | |  | |  | |  | |  | | Tausd/µl | 0.25 - 0.85 |
| Eosinophile abs |  |  | | 0.06 | |  | |  | |  | |  | | Tausd/µl | 0.03 - 0.47 |
| Basophile abs |  |  | | 0.04 | |  | |  | |  | |  | | Tausd/µl | 0.02 - 0.11 |
| Unreife Granulozyten abs |  |  | | 0.01 | |  | |  | |  | |  | | Tausd/µl | 0 - 0.06 |
| Neutrophile |  |  | | 63.4 | |  | |  | |  | |  | | % | 40.0 - 80.0 |
| Lymphozyten |  |  | | 28.5 | |  | |  | |  | |  | | % | 20.0 - 45.0 |
| Monozyten |  |  | | 6.2 | |  | |  | |  | |  | | % | 2.0 - 13 |
| Eosinophile |  |  | | 1.0 | |  | |  | |  | |  | | % | 0.5 - 8 |
| Basophile |  |  | | 0.7 | |  | |  | |  | |  | | % | max. 2.0 |
| Unreife Granulozyten |  |  | | 0.2 | |  | |  | |  | |  | | % | 0 - 0.6 |
| Normoblasten abs |  |  | | 0 | | 0 | |  | |  | |  | | Tausd/µl | 0 - 0.1 |
| Normoblasten |  |  | | 0 | | 0 | |  | |  | |  | | % | 0 - 1 |
|  | | | | | | | | | | | | | | | |
| Erythrozyten |  |  | | 4.86 | | 4.51 | |  | |  | |  | | Mio/µl | 4.2 - 6.2 |
| Haematokrit |  |  | | 41.5 | | 38.5 | |  | |  | |  | | % | 37.0 - 47.0 |
| HB |  |  | | 14.5 | | 13.4 | |  | |  | |  | | g/dl | 12.0 - 16.0 |
| MCH |  |  | | 29.8 | | 29.7 | |  | |  | |  | | pg | 27.0 - 34.0 |
| MCHC |  |  | | 34.9 | | 34.8 | |  | |  | |  | | g/dl | 32.0 - 36 |
| MCV |  |  | | 85.4 | | 85.4 | |  | |  | |  | | fl | 80 - 93 |
| Ery. Verteilungsbreite (RDW) |  |  | | 12.2 | | 12.1 | |  | |  | |  | | % | max. 15 |
| Thrombozyten |  |  | | 244 | | 193 | |  | |  | |  | | Tausd/µl | 150 - 450 |
| Verteilungsbreite der Thrombozyten |  |  | | 11.9 | | 12.4 | |  | |  | |  | | fl | 10 - 16 |
| Mittleres Plättchenvolumen |  |  | | 10.3 | | 11.0 | |  | |  | |  | | fl | 9 - 12 |
| **Gerinnung: Citrat-Blut** | | | | | | | | | | | | | | | |
| Quick |  |  | | 115 | |  | |  | |  | |  | | % | 70 - 120 |
| INR |  |  | | 0.9 | |  | |  | |  | |  | |  |  |
| aPTT |  |  | | 24 | |  | |  | |  | |  | | sec | max. 40 |
| **Elektrolyte: Li-Heparin-Plasma** | | | | | | | | | | | | | | | |
| Natrium |  |  | | 141 | | 139 | |  | |  | |  | | mmol/l | 136 - 148 |
| Kalium |  |  | | 4.3 | | 3.8 | |  | |  | |  | | mmol/l | 3.5 - 4.8 |
| Calcium |  |  | | 2.2 | | 2.2 | |  | |  | | 2.3 | | mmol/l | 2.1 - 2.6 |
| Anorganisches Phosphat |  |  | | 0.9 | |  | |  | |  | |  | | mmol/l | 0.8 - 1.5 |
| **Substrate: Li-Heparin-Plasma** | | | | | | | | | | | | | | | |
| Kreatinin |  |  | | 0.8 | | 0.7 | |  | |  | | 0.8 | | mg/dl | 0.5 - 0.8 |
| GFR - CKD-EPI |  |  | | 88 | |  | |  | |  | | 88 | | ml/min/ 1,73m2 |  |
| Harnstoff |  |  | | 36 | |  | |  | |  | | 23 | | mg/dl | 12 - 46 |
| Harnsäure |  |  | | 5.7 | |  | |  | |  | |  | | mg/dl | 2.4 - 5.7 |
| Bilirubin, gesamt |  |  | |  | |  | |  | |  | | 0.6 | | mg/dl | max. 1.1 |
| Gesamt-Eiweiss |  |  | | 7.3 | |  | |  | |  | |  | | g/dl | 6.5 - 8.5 |
| Cholesterin |  |  | | 218 | |  | |  | |  | |  | | mg/dl | 130 - 190 |
| HDL-Cholesterin |  |  | | 65 | |  | |  | |  | |  | | mg/dl | min. 45 |
| LDL-Cholesterin |  |  | | 153 | |  | |  | |  | |  | | mg/dl | max. 160 |
| Triglyzeride |  |  | | 65 | |  | |  | |  | |  | | mg/dl | max. 200 |
| C-reaktives Protein (CRP) |  |  | | 0.43 | | 0.33 | |  | |  | | 0.64 | | mg/dl | max. 0.50 |
| **Enzyme: Li-Heparin-Plasma** | | | | | | | | | | | | | | | |
| Creatinkinase (CK) |  |  | | 87 | | 49 | |  | |  | | 99 | | U/l | max. 170 |
| GOT/AST |  |  | |  | |  | |  | |  | | 9 | | U/l | max. 35 |
| GPT/ALT |  |  | | 13 | |  | |  | |  | | 15 | | U/l | max. 34 |
| Alkalische Phosphatase (AP) |  |  | |  | |  | |  | |  | | 76 | | U/l | 35 - 105 |
| Laktatdehydrogenase (LDH) |  |  | | 205 | |  | |  | |  | | 212 | | U/l | max. 250 |
| Gamma-Glutamyl-Transferase (GGT) |  |  | | 18 | |  | |  | |  | | 16 | | U/l | max. 40 |
| Lipase |  |  | |  | |  | |  | |  | | 39 | | U/l | max. 60 |
| **Hormone: Li-Heparin-Plasma** | | | | | | | | | | | | | | | |
| TSH |  |  | |  | |  | |  | |  | | 3.01 | | mU/l | 0.5 - 4.4 |
| **Hormone: EDTA-Plasma** | | | | | | | | | | | | | | | |
| Renin nach Orthostase |  |  | | 33 | |  | |  | |  | |  | | ng/l | 7 - 42 |
| Aldosteron nach Orthostase |  |  | | 146 | |  | |  | |  | |  | | ng/l | 13 - 230 |
| **Hormone: Serum** | | | | | | | | | | | | | | | |
| TSH |  |  | | 2.06 | |  | |  | |  | |  | | mU/l | 0.5 - 4.4 |
| **Laktat, Glukose: Na-F-Plasma** | | | | | | | | | | | | | | | |
| Laktat, photometrisch |  |  | |  | |  | |  | |  | | 0.8 | | mmol/l | 0.5 - 2.2 |
| Glukose venös |  |  | |  | |  | |  | |  | | 85 | | mg/dl | 70 - 99 |
| **Sonstiges: EDTA-Blut** | | | | | | | | | | | | | | | |
| HbA1c (IFCC) |  |  | | 33.0 | |  | |  | |  | |  | | mmol/mol | 26 - 44 |
| HbA1c (glykiertes Hämoglobin) |  |  | | 5.2 | |  | |  | |  | |  | | % | 4.5 - 6.2 |
| **Urinstatus: Spontanurin** | | | | | | | | | | | | | | | |
| Urinstatus |  |  | | Urinstatus | |  | |  | |  | |  | |  |  |
| Farbe des Spontanurin |  |  | | Hellgelb | |  | |  | |  | |  | |  |  |
| Trübung im Spontanurin |  |  | | klar | |  | |  | |  | |  | |  |  |
| Spez.Gewicht im Spontanurin |  |  | | 1.009 | |  | |  | |  | |  | | g/cmE3 |  |
| pH-Wert im Spontanurin |  |  | | 7.0 | |  | |  | |  | |  | |  |  |
| Eiw.Qual. im Spontanurin |  |  | | N. | |  | |  | |  | |  | |  |  |
| Gluk.Qual. im Spontanurin |  |  | | N. | |  | |  | |  | |  | |  |  |
| U-Keton im Spontanurin |  |  | | N. | |  | |  | |  | |  | |  |  |
| Hb Qual. im Spontanurin |  |  | | N. | |  | |  | |  | |  | |  |  |
| Urobilinogen im Spontanurin |  |  | | N. | |  | |  | |  | |  | |  |  |
| Bilirubin im Spontanurin |  |  | | N. | |  | |  | |  | |  | |  |  |
| Leukos im Spontanurin |  |  | | N. | |  | |  | |  | |  | |  |  |
| Nitrit im Spontanurin |  |  | | N. | |  | |  | |  | |  | |  |  |
| **Urinsediment (maschinell, digitale Mikroskopie)** | | | | | | | | | | | | | | | |
| Erythrozyten |  |  | | 5 | |  | |  | |  | |  | | 1/µl | 0 - 7 |
| Leukozyten |  |  | | 3 | |  | |  | |  | |  | | 1/µl | 0 - 9 |
| Bakterien |  |  | | (+) | |  | |  | |  | |  | |  | max. negativ |
| Plattenepithel |  |  | | (+) | |  | |  | |  | |  | |  | max. (+) |
| Nichtplattenepithelien |  |  | | (+) | |  | |  | |  | |  | |  | max. (+) |
| **Sonstiges: Spontanurin** | | | | | | | | | | | | | | | |
| Kreatinin Konz. im Spontanurin |  |  | | 55 | |  | |  | |  | |  | | mg/dl |  |
| Eiweiss-Konz. im Spontanurin |  |  | | 0.09 | |  | |  | |  | |  | | g/l | max. 0.10 |
| Quot. U-Eiw / U-Krea (Spontanurin) |  |  | | 164 | |  | |  | |  | |  | | mg/g Krea | max. 100 |
| Quot. U-Eiw/U-Krea (Spontanurin) |  |  | | 18.49 | |  | |  | |  | |  | | mg/mmol Crea | max. 11.3 |
| Albumin Konzentration |  |  | | 35 | |  | |  | |  | |  | | mg/l | max. 20 |
| Quot. U-Albumin /g Krea |  |  | | 63.6 | |  | |  | |  | |  | | mg/g Krea | max. 20 |
| A1-Mikroglobulin Konzentration |  |  | | <5 | |  | |  | |  | |  | | mg/l | max. 13 |
| A2-Makroglobulin Konzentration |  |  | | <2.5 | |  | |  | |  | |  | | mg/l | 0 - <2.4 |
| IgG-Konzentration im Spontanurin |  |  | | 4 | |  | |  | |  | |  | | mg/l | max. 10 |
| U-IgG / Albumin-Quot. im Spontanurin |  |  | | 0.1 | |  | |  | |  | |  | |  | max. 0.2 |
| IgG /g Krea |  |  | | 7.3 | |  | |  | |  | |  | | mg/g Krea | max. 10 |
|  | | | | | | | | | | | | | | | |
| Gesamt-Metanephrine im Sammelurin | 254 |  | |  | |  | |  | |  | |  | | µg/24h | < 900 |
| Freies Cortisol im Sammelurin |  | 38 | |  | |  | |  | |  | |  | | µg/24h | 4,2 - 60 |

**b) Diabetes mellitus**

|  |  | |
| --- | --- | --- |
|  | | **University hospital** Department of Internal Medicine IV  Diabetology  Endocrinology  Nephrology  Medical Director  Prof.Konrad Kaiser, MD  Otfried-Müller-Straße 10  72076 Tuebingen  Service telephone: 07071 29- 123456  Fax: 07071 29- 23456  Contact person:  Maximilian Möller, MD |
| University hospital ▪ Otfried-Müller-Str. 10 ▪ 72076 Tübingen | |  |
| Mr  Anton Arzt, MD  General practitioner  Arztweg 2  72070 Tübingen | |  |
|  | |  |
|  | |  |

Patient: **Sebastian Sweet,** date of birth: **06.05.1953** Patient case no. 1 **23456789**

Address: **Marsstraße 66, 72 070 Tübingen**

**Ward 75:** Admission **04.10.2023** / Discharge **08.10.2023** Please have ready in case of queries

Dear colleague,

We are reporting on the inpatient stay of the above-mentioned patient on our ward 75.

**Diagnoses:**

|  | **Hyperglycemic decompensation with initial diagnosis of type 2 diabetes 10/2023**   - Clinic: Symptomatic with weight loss and polyuria - BG max. 522 mg/dl, HbA1c currently 14.1 %, C-peptide-glucose ratio ~ 3.0,  ketone bodies 0.4 mmol/l - Autoantibodies: IA2 negative at 0.1 IU/ml, GAD negative at 0.7 IU/ml - Sonography of the pancreas: No sign of malignancies - Therapy: basal supported oral therapy with insulin glargine, metformin and empagliflozin - Complications: Polyneuropathy |
| --- | --- |
|  | Coxarthrosis left |
|  | Status post appendectomy 1972 |
|  | Status post onychomycosis on both sides |
|  | Hay fever |

**Therapy recommendation:**

Metformin 500 mg 1-0-1, NEW

Empagliflozin 10 mg 1-0-0, NEW

Insulin glargine 100 IU/ml 14 IU at 8 o'clock in the morning, NEW

Atorvastatin 20 mg 1-0-0, NEW

**Basal insulin adaptation plan (Lantus):**

- If fasting blood glucose is **greater than 150 mg/dl** on 3 consecutive days, inject **2 IU** more insulin.
- If fasting blood glucose is **less than 100 mg/dl**, inject **4 IU** less insulin.

**Procedure:**

- We recommend that you attend ophthalmological, cardiological and nephrological check-ups on an outpatient basis
- HbA1c checks every 3 months (target corridor based on age 7.5 to 8 %, without hypoglycemia)

**Medical history:**

S. Sweet was admitted to hospital following the initial diagnosis of diabetes mellitus by her GP. She had been seen by her GP a week earlier and had lost 10 kg of weight within a month and was feeling exhausted. S. Sweet also reported polyuria, especially with nocturia 5 times/night and xerostomia. The amount drunk was about 1.5-2 liters. No fever, no night sweats, no exertional dyspnea. Bowel movements unremarkable every 2 days. Appetite good, no nausea, no vomiting. Laboratory tests today showed an HbA1c of 14.0 % and a glucose value of 324 mg/dl on an empty stomach. No drug therapy for diabetes mellitus had yet been started. The patient had not had a family doctor for years. No previous chronic illnesses are known, no medication taken. About 3 months ago, he saw an orthopedic surgeon with hip pain, where he was diagnosed with coxarthrosis and received an injection in his groin; he currently has no more complaints. The patient lives alone at home after the death of his wife 3 years ago, his daughter visits about once a week. Non-smoker, regular alcohol consumption (2-3 beers/day). No allergies.

**Physical examination findings:**

Vital signs: HR: 66/min, RR: 130/80 mmHg, Temp: 37.2°C, AF: 15/min (04/10/2023 12:06)

Head/neck: Cervical lymph nodes and thyroid gland not palpable enlarged, oral mucosa dry

Lungs: ventilation equal to the side, ubiquitous vesicular breath sound, no shortening of the knocking sound

Heart: Heart sounds quiet, as far as can be assessed pure, rhythmic, no heart murmurs

Abdomen: abdomen expansive, bowel sounds lively, abdominal wall soft, no pressure/loosening pain

Extremities/vessels: postural tremor of both hands, no edema

Skin: normal turgor, long history of pre-existing hypopigmentation on the thighs

Diabetic foot examination:

Feet dry, skin intact, hyperkeratosis on toes and pads.

Vibration sensation severely restricted (tuning fork at the metacarpophalangeal joint of the thumb 6/8, knee 0/8, inner ankle 0/8), epicritical sensitivity unrestricted on examination with microfilament. History of frequent numbness in the forefoot area. Foot pulses palpable on both sides.

**Laboratory:**

Please refer to the enclosed appendix for the laboratory results in detail.

**Summarized assessment:**

S. Sweet was admitted to hospital by his GP due to hyperglycemic decompensation following the initial diagnosis of diabetes mellitus. Clinically, S. Sweet had noticed weight loss and pronounced polyuria in recent weeks. The GP's laboratory tests revealed an HbA1c of 14%, which led to the patient being admitted to our emergency department. On admission, laboratory tests revealed a blood glucose level of 522 mg/dl. At 14.1%, the HbA1c was well above the normal value. There was no evidence of ketoacidosis on admission. A rapid clinical improvement was achieved with forced volume substitution and sequential insulin therapy. A sonography of the pancreas showed no evidence of a mass. Based on the clinic, age, C-peptide-glucose ratio of 3 and negative autoantibodies, S. Sweet was diagnosed with type 2 diabetes mellitus.

S. Sweet was put on a BOT program with insulin glargine, metformin and empagliflozin. During therapy, fasting blood glucose checks once or twice a week are recommended for therapy control. Satisfactory blood glucose levels of around 130 mg/dl were achieved during antidiabetic therapy. In addition, S. Sweet received intensive training from our diabetes/nutrition counsellor on the correct implementation of insulin therapy, blood glucose measurement and how to recognize and counteract hypo- and hyperglycemia.

In particular, attention must be paid to the occurrence of symptoms of hypoglycemia such as palpitations, tremor or changes in consciousness. The current daily alcohol consumption must be urgently reduced in view of the associated risk of hypoglycemia. We recommend regular monitoring of HbA1c and regular ophthalmological, cardiological and nephrological check-ups to rule out secondary diseases. Currently, diabetic polyneuropathy was found with clearly pronounced pallhypesthesia in both feet. Regular self-inspections of the feet should be carried out for early detection of diabetic foot syndrome. Fortunately, renal function was unremarkable without microalbuminuria.

With an LDL cholesterol of 192 mg/dl (target < 100 mg/dl), statin therapy to lower lipids is currently indicated. We therefore recommend starting atorvastatin as described above. If muscle pain occurs during therapy, a doctor should be consulted to evaluate statin-induced myopathy. In addition, moderate physical activity of at least 150 minutes per week should be aimed at to reduce the cardiovascular risk.

We were able to release S. Sweet in good condition on 8 October 2023. Please follow the above procedure.

With best regards

| Prof Konrad Kaiser, MD  Medical Director | Susanne Steeg, MD  Senior Physician Endocrinology | Maximilian Möller, MD  Resident |
| --- | --- | --- |

**Laboratory**

| Destination | 08.10.23 | 06.10.23 | | 05.10.23 | | 04.10.23 | | 04.10.23 | |  | |  | | Unit | Reference |
| --- | --- | --- | --- | --- | --- | --- | --- | --- | --- | --- | --- | --- | --- | --- | --- |
| Order | 07:00 | 08:00 | | 06:00 | | 14:57 | | 10:44 | |  | |  | | | |
| Order number |  | |  | |  | |  | |  | |  | |  | | |
| **(Diff) blood count: EDTA blood** | | | | | | | | | | | | | | | |
| Leukocytes | 4680 |  | | 7590 | |  | | 8480 | |  | |  | | 1/µl | 3800 - 10300 |
| Neutrophils abs |  |  | | 3.47 | |  | |  | |  | |  | | Thousand/µl | 1.80 - 7.00 |
| Lymphocytes abs |  |  | | 3.22 | |  | |  | |  | |  | | Thousand/µl | 1.1 - 3.2 |
| Monocytes abs |  |  | | 0.59 | |  | |  | |  | |  | | Thousand/µl | 0.26 - 0.87 |
| Eosinophil abs |  |  | | 0.23 | |  | |  | |  | |  | | Thousand/µl | 0.03 - 0.47 |
| Basophilic abs |  |  | | 0.04 | |  | |  | |  | |  | | Thousand/µl | 0.02 - 0.11 |
| Immature granulocytes abs |  |  | | 0.04 | |  | |  | |  | |  | | Thousand/µl | 0 - 0.06 |
| Neutrophils |  |  | | 45.8 | |  | |  | |  | |  | | % | 40.0 - 80.0 |
| Lymphocytes |  |  | | 42.4 | |  | |  | |  | |  | | % | 20.0 - 45.0 |
| Monocytes |  |  | | 7.8 | |  | |  | |  | |  | | % | 2.0 - 13 |
| Eosinophils |  |  | | 3.0 | |  | |  | |  | |  | | % | 0.5 - 8 |
| Basophile |  |  | | 0.5 | |  | |  | |  | |  | | % | max. 2.0 |
| Immature granulocytes |  |  | | 0.5 | |  | |  | |  | |  | | % | 0 - 0.6 |
| Erythrocytes | 4.4 |  | | 4.27 | |  | | 4.64 | |  | |  | | Mio/µl | 4.20 - 6.20 |
| Haematocrit | 42.7 |  | | 38.2 | |  | | 42.6 | |  | |  | | % | 42.0 - 52.0 |
| HB | 14.3 |  | | 13.7 | |  | | 14.5 | |  | |  | | g/dl | 14.0 - 18.0 |
| MCH | 32.4 |  | | 32.1 | |  | | 31.3 | |  | |  | | pg | 27.0 - 34.0 |
| MCHC | 35.3 |  | | 35.9 | |  | | 34.0 | |  | |  | | g/dl | 32.0 - 36 |
| MCV | 91.7 |  | | 89.5 | |  | | 91.8 | |  | |  | | fl | 80 - 93 |
| Ery. Distribution width (RDW) | 13.0 |  | | 12.8 | |  | | 12.8 | |  | |  | | % | max. 15.0 |
| Thrombocytes | 166 |  | | 190 | |  | | 225 | |  | |  | | Thousand/µl | 150 - 450 |
| Distribution range of the thrombocytes | 11.2 |  | | 12.0 | |  | | 11.3 | |  | |  | | fl | 10 - 16 |
| Medium platelet volume | 10.0 |  | | 10.0 | |  | | 9.7 | |  | |  | | fl | 9 - 12 |
| **Coagulation: citrated blood** | | | | | | | | | | | | | | | |
| Quick |  |  | |  | |  | | 92 | |  | |  | | % | 70 - 120 |
| INR |  |  | |  | |  | | 1.0 | |  | |  | |  |  |
| **Electrolytes: Li-heparin plasma** | | | | | | | | | | | | | | | |
| Sodium | 136 |  | | 137 | |  | | 133 | |  | |  | | mmol/l | 136 - 148 |
| Potassium | 3.6 |  | | 4.1 | |  | | 4.3 | |  | |  | | mmol/l | 3.5 - 4.8 |
| **Substrates: Li-heparin plasma** | | | | | | | | | | | | | | | |
| Creatinine (enzymatic) | 0.9 |  | | 0.8 | |  | | 1.0 | |  | |  | | mg/dl | 0.6 - 1.1 |
| GFR - MDRD | 81 |  | | 93 | |  | | 72 | |  | |  | | ml/min/ 1,73m2 | min. > 60 |
| GFR - CKD-EPI | 81 |  | | 85 | |  | | 71 | |  | |  | | ml/min/ 1,73m2 |  |
| Urea | 23 |  | | 22 | |  | | 36 | |  | |  | | mg/dl | 12 - 46 |
| Bilirubin, total |  |  | |  | |  | | 1,1 | |  | |  | | mg/dl | max. 1.1 |
| Cholesterol |  |  | |  | |  | | 295 | |  | |  | | mg/dl | 130 - 190 |
| HDL cholesterol |  |  | |  | |  | | 35 | |  | |  | | mg/dl | min. 35 |
| LDL cholesterol |  |  | |  | |  | | 192 | |  | |  | | mg/dl | max. 160 |
| Triglycerides |  |  | |  | |  | | 306 | |  | |  | | mg/dl | max. 200 |
| C-reactive protein (CRP) | <0.05 |  | | <0.05 | |  | | <0.05 | |  | |  | | mg/dl | max. 0.50 |
| **Enzymes: Li-heparin plasma** | | | | | | | | | | | | | | | |
| GOT/AST |  |  | |  | |  | | 37 | |  | |  | | U/l | max. 50 |
| Alkaline phosphatase (AP) |  |  | |  | |  | | 97 | |  | |  | | U/l | 40 - 130 |
| Gamma-glutamyl transferase (GGT) |  |  | |  | |  | | 55 | |  | |  | | U/l | max. 60 |
| **Other: Li-heparin plasma** | | | | | | | | | | | | | | | |
| Osmolality |  |  | |  | |  | | 298 | |  | |  | | mOsm/kg | 275 - 300 |
| **Hormones: Serum** | | | | | | | | | | | | | | | |
| TSH |  |  | |  | |  | | 3.09 | |  | |  | | mU/l | 0.5 - 4.4 |
| C-peptide |  |  | |  | |  | | 1166 | |  | |  | | pmol/l | 140 - 830 |
| GAD antibodies | Neg. |  | |  | |  | |  | |  | |  | |  |  |
| IA2 - Antibodies | Neg. |  | |  | |  | |  | |  | |  | |  |  |
| **Lactate, glucose: Na-F plasma** | | | | | | | | | | | | | | | |
| Venous glucose |  |  | |  | |  | | 430 | |  | |  | | mg/dl | 70 - 99 |
| **Other: EDTA blood** | | | | | | | | | | | | | | | |
| HbA1c (HPLC) |  |  | |  | |  | | 130.4 | |  | |  | | mmol/mol | 26.0 - 44.0 |
| HbA1c (glycated haemoglobin) |  |  | |  | |  | | 14.1 | |  | |  | | % | 4.5 - 6.2 |
| **Urine status: Spontaneous urine** | | | | | | | | | | | | | | | |
| Urine status |  |  | |  | | Urine status | | Urine status | |  | |  | |  |  |
| Colour of the spontaneous urine |  |  | |  | | Light yellow | | Straw colours | |  | |  | |  |  |
| Turbidity in spontaneous urine |  |  | |  | | clear | | clear | |  | |  | |  |  |
| Specific gravity in spontaneous urine |  |  | |  | | 1.029 | | 1.030 | |  | |  | | g/ml |  |
| pH value in spontaneous urine |  |  | |  | | 5.0 | | 5.0 | |  | |  | |  |  |
| Protein quality in spontaneous urine |  |  | |  | | N. | | N. | |  | |  | |  |  |
| Gluc.qual. in spontaneous urine |  |  | |  | | ++++ | | ++++ | |  | |  | |  |  |
| U-ketone in spontaneous urine |  |  | |  | | N. | | N. | |  | |  | |  |  |
| Hb qual. in spontaneous urine |  |  | |  | | N. | | N. | |  | |  | |  |  |
| Urobilinogen in spontaneous urine |  |  | |  | | N. | | N. | |  | |  | |  |  |
| Bilirubin in spontaneous urine |  |  | |  | | N. | | N. | |  | |  | |  |  |
| Leukocytes in spontaneous urine |  |  | |  | | N. | | N. | |  | |  | |  |  |
| Nitrite in spontaneous urine |  |  | |  | | N. | | N. | |  | |  | |  |  |
| **Other: Spontaneous urine** | | | | | | | | | | | | | | | |
| Creatinine conc. in spontaneous urine |  | 41 | |  | |  | | 64 | |  | |  | | mg/dl |  |
| Protein concentration in spontaneous urine |  | <0.06 | |  | |  | | <0.06 | |  | |  | | g/l | max. 0.10 |
| Albumin concentration |  | <11 | |  | |  | | <11 | |  | |  | | mg/l | max. 20 |

|  |  | |
| --- | --- | --- |
|  | | **Medizinische Klinik** Abteilung Innere Medizin IV  Diabetologie  Endokrinologie  Nephrologie  Ärztlicher Direktor  Prof. Dr. med. Konrad Kaiser  Otfried-Müller-Straße 10  72076 Tübingen  Servicetelefon: 07071 29-123456  Fax: 07071 29-23456  Ansprechpartner:  Herr Dr. Maximilian Möller |
| Medizinische Klinik ▪ Otfried-Müller-Str. 10 ▪ 72076 Tübingen | |  |
| Herrn  Dr. med. Antonia Arzt  FA für Allgemeinmedizin  Arztweg 2  72070 Tübingen | |  |
|  | |  |
|  | |  |

Patient: **Sebastian Süß,** Geb.-Datum: **06.05.1953** Patienten-Fall-Nr. **123456789**

wohnhaft: **Marsstraße 66, 72070 Tübingen**

**Station 75:** Aufnahme **04.10.2023** / Entlassung **08.10.2023** bitte bei Rückfragen bereithalten

Sehr geehrter Herr Kollege,

wir berichten über den stationären Aufenthalt des/r oben genannten Patienten/in auf unserer Station 75.

**Diagnosen:**

|  | **Hyperglykämische Dekompensation bei Erstdiagnose Typ-2-Diabetes ED 10/2023**   - Klinik: Symptomatisch mit Gewichtsverlust und Polyurie - BZ max. 522 mg/dl, HbA1c aktuell 14,1 %, C-Peptid-Glucose-Ratio ~ 3,0, Ketonkörper 0,4 mmol/l - Autoantikörper: IA2 negativ mit 0,1 IU/ml, GAD negativ mit 0,7 IU/ml - Sonografie Pankreas: Kein Hinweis auf Raumforderung - Therapie: BOT mit Insulin glargin, Metformin und Empagliflozin - Folgeerkrankungen: Polyneuropathie |
| --- | --- |
|  | Coxarthrose links |
|  | Z.n. Appendektomie 1972 |
|  | Z.n. Onychomykose beidseits |
|  | Heuschnupfen |

**Therapieempfehlung:**

Metformin 500 mg 1-0-1, NEU

Empagliflozin 10 mg 1-0-0, NEU

Insulin glargin 100 E/ml 14 E um 8 Uhr morgens, NEU

Atorvastatin 20 mg 1-0-0, NEU

**Basalinsulin Anpassungsplan (Lantus):**

- Wenn Nüchternblutzucker an 3 Tagen hintereinander **größer als 150 mg/dl** ist, dann **2 IE** mehr Insulin spritzen.
- Wenn Nüchternblutzucker **kleiner als 100 mg/dl** ist, dann **4 IE** weniger Insulin spritzen.

**Procedere:**

- Wir empfehlen die Wahrnehmung von augenärztlichen, kardiologischen und nephrologischen Kontrollen im ambulanten Rahmen
- Alle 3 Monate Kontrollen von HbA1c (Zielkorridor aufgrund Lebensalter 7,5 bis 8 %, ohne Hypoglykämien)

**Anamnese:**

Die stationäre Aufnahme von S. Süß erfolgte nach Erstdiagnose eines Diabetes mellitus durch die Hausärztin. Eine Vorstellung bei der Hausärztin erfolgte eine Woche zuvor bei einem Gewichtsverlust von 10 kg innerhalb von einem Monat und Abgeschlagenheit. Außerdem berichtet S. Süß über Polyurie, insbesondere mit Nykturie 5x/Nacht und Xerostomie. Die Trinkmenge betrage etwa 1,5-2L. Kein Fieber, kein Nachtschweiß, keine Belastungsdyspnoe. Stuhlgang unauffällig alle 2 Tage. Appetit gut, keine Übelkeit, kein Erbrechen. Laborchemisch war heute ein HbA1c von 14,0 %, sowie ein Glukosewert von nüchtern 324 mg/dl aufgefallen. Bisher sei noch keine medikamentöse Therapie des Diabetes mellitus begonnen worden. Der Patient habe davor jahrelang keinen Hausarzt gehabt. Chronische Vorerkrankungen sind nicht bekannt, keine Medikamenteneinnahme. Vor etwa 3 Monaten Vorstellung beim Orthopäden mit Hüftschmerzen, dabei sei eine Coxarthrose diagnostiziert worden und er habe eine Injektion an der Leiste erhalten, aktuell habe er keine Beschwerden mehr. Der Patient lebt nach Tod der Ehefrau vor 3 Jahren alleine zu Hause, die Tochter komme etwa 1x/Woche zu Besuch. Nichtraucher, regelmäßiger Alkoholkonsum (2-3 Bier/Tag). Keine Allergien.

**Körperlicher Untersuchungsbefund:**

Vitalparameter: HF: 66/min, RR: 130/80 mmHg, Temp: 37,2°C, AF: 15/min (04.10.2023 12:06)

Kopf/Hals: Zervikale Lymphknoten und Schilddrüse nicht vergrößert tastbar, Mundschleimhaut trocken

Lunge: Belüftung seitengleich, ubiquitär vesikuläres Atemgeräusch, keine Klopfschallverkürzung

Herz: Herztöne leise, soweit beurteilbar rein, rhythmisch, keine Herzgeräusche

Abdomen: Abdomen ausladend, Darmgeräusche lebhaft, Bauchdecken weich, kein Druck-/Loslassschmerz

Extremitäten/Gefäße: Haltetremor beider Hände, keine Ödeme

Haut: Turgor normal, anamnestisch lange vorbestehende Hypopigmentierung an den Oberschenkeln

Diabetische Fußuntersuchung:

Füße trocken, Haut intakt, Hyperkeratosen an Zehen und Ballen.

Vibrationsempfinden stark eingeschränkt (Stimmgabel am Daumengrundgelenk 6/8, Knie 0/8, Innenknöchel 0/8), epikritische Sensibilität bei Untersuchung mit Mikrofilament uneingeschränkt. Anamnestisch häufig Taubheitsgefühle im Vorfußbereich. Fußpulse beidseits tastbar.

**Labor:**

Die Laborbefunde im Einzelnen entnehmen Sie bitte beiliegendem Anhang.

**Zusammenfassende Beurteilung:**

Die stationäre Aufnahme von S. Süß erfolge als Einweisung durch die Hausärztin aufgrund einer hyperglykämischen Dekompensation bei Erstdiagnose eines Diabetes mellitus. Klinisch hatte S. Süß in den letzten Wochen einen Gewichtsverlust sowie eine ausgeprägte Polyurie bemerkt. Bei der Hausärztin war in der Laborkontrolle ein HbA1c von 14% aufgefallen, was zur klinischen Einweisung des Patienten in unsere Notaufnahme führte. Bei Aufnahme zeigte sich laborchemisch ein Blutglukosewert von 522 mg/dl. Der HbA1c war mit 14,1 % deutlich über dem Normwert. Eine Ketoazidose war bei Aufnahme nicht nachweisbar. Unter einer forcierten Volumensubstitution und sequentieller Insulintherapie war eine rasche klinische Besserung zu erzielen. Eine Sonografie des Pankreas zeigte keinen Hinweis auf eine Raumforderung. Aufgrund der Klinik, des Alters, der C-Peptid-Glucose-Ratio von 3 und negativen Autoantikörpern ist in Zusammenschau der Befunde bei S. Süß ein Diabetes mellitus Typ II zu diagnostizieren.

Therapeutisch wurde S. Süß auf ein BOT-Schema mit Insulin glargin, Metformin und Empagliflozin eingestellt. Unter der Therapie sind ein- bis zwei Mal wöchentliche Blutzuckerkontrollen nüchtern zur Therapiesteuerung zu empfehlen. Unter der antidiabetischen Therapie zeigten sich zufriedenstellende Blutzuckerwerte um 130 mg/dl. Zudem wurde S. Süß intensiv durch unsere Diabetes- /Ernährungsberatung hinsichtlich einer korrekten Umsetzung der Insulintherapie, der Messung des Blutzuckers und bezüglich des Erkennens und Entgegensteuerns von Hypo- und Hyperglykämien geschult.

Insbesondere muss auf das Auftreten von Symptomen einer Hypoglykämie wie Palpitationen, Tremor oder Bewusstseinsveränderungen geachtet werden. Der aktuell bestehende tägliche Alkoholkonsum muss vor dem Hintergrund des damit verbundenen Risikos für Hypoglykämien dringend reduziert werden. Wir raten zu einer regelmäßigen Überwachung des HbA1c und regelmäßigen augenärztlichen, kardiologischen sowie nephrologischen Kontrolluntersuchungen zum Ausschluss von Folgeerkrankungen. Aktuell zeigte sich eine diabetische Polyneuropathie bei deutlich ausgeprägter Pallhypästhesie an beiden Füßen. Hier sollten regelmäßige Selbstinspektionen der Füße zur Früherkennung eines diabetischen Fußsyndroms erfolgen. Von renaler Seite zeigte sich erfreulicherweise eine unauffällige Nierenfunktion ohne Mikroalbuminurie.

Bei einem LDL-Cholesterin von 192 mg/dl (Ziel < 100 mg/dl) ist eine Statintherapie zur Lipidsenkung aktuell indiziert. Wir empfehlen daher einen Beginn mit Atorvastatin wie oben angegeben. Sollte es unter der Therapie zu Muskelschmerzen kommen, sollte eine ärztliche Vorstellung zur Evaluation eine Statin-induzierten Myopathie erfolgen. Zudem sollte eine moderate körperliche Aktivität über mindestens 150 min pro Woche zur Senkung des kardiovaskulären Risikos angestrebt werden.

Am 08.10.2023 konnten wir S. Süß in gutem Zustand wieder entlassen. Wir bitten um Beachtung des oben genannten Procederes.

Mit freundlichen kollegialen Grüßen

| Prof. Dr. med. Konrad Klaiber  Ärztlicher Direktor | Dr. med. Susanne Steeg  Oberärztin Endokrinologie | Dr. Maximilian Möller  Assistenzarzt |
| --- | --- | --- |

**Labor**

| Bestimmung | 08.10.23 | 06.10.23 | | 05.10.23 | | 04.10.23 | | 04.10.23 | |  | |  | | Einheit | Referenz |
| --- | --- | --- | --- | --- | --- | --- | --- | --- | --- | --- | --- | --- | --- | --- | --- |
| Auftrag | 07:00 | 08:00 | | 06:00 | | 14:57 | | 10:44 | |  | |  | | | |
| Auftrag-Nummer |  | |  | |  | |  | |  | |  | |  | | |
| **(Diff)-Blutbild: EDTA-Blut** | | | | | | | | | | | | | | | |
| Leukozyten | 4680 |  | | 7590 | |  | | 8480 | |  | |  | | 1/µl | 3800 - 10300 |
| Neutrophile abs |  |  | | 3.47 | |  | |  | |  | |  | | Tausd/µl | 1.80 - 7.00 |
| Lymphozyten abs |  |  | | 3.22 | |  | |  | |  | |  | | Tausd/µl | 1.1 - 3.2 |
| Monozyten abs |  |  | | 0.59 | |  | |  | |  | |  | | Tausd/µl | 0.26 - 0.87 |
| Eosinophile abs |  |  | | 0.23 | |  | |  | |  | |  | | Tausd/µl | 0.03 - 0.47 |
| Basophile abs |  |  | | 0.04 | |  | |  | |  | |  | | Tausd/µl | 0.02 - 0.11 |
| Unreife Granulozyten abs |  |  | | 0.04 | |  | |  | |  | |  | | Tausd/µl | 0 - 0.06 |
| Neutrophile |  |  | | 45.8 | |  | |  | |  | |  | | % | 40.0 - 80.0 |
| Lymphozyten |  |  | | 42.4 | |  | |  | |  | |  | | % | 20.0 - 45.0 |
| Monozyten |  |  | | 7.8 | |  | |  | |  | |  | | % | 2.0 - 13 |
| Eosinophile |  |  | | 3.0 | |  | |  | |  | |  | | % | 0.5 - 8 |
| Basophile |  |  | | 0.5 | |  | |  | |  | |  | | % | max. 2.0 |
| Unreife Granulozyten |  |  | | 0.5 | |  | |  | |  | |  | | % | 0 - 0.6 |
| Erythrozyten | 4.4 |  | | 4.27 | |  | | 4.64 | |  | |  | | Mio/µl | 4.20 - 6.20 |
| Haematokrit | 42.7 |  | | 38.2 | |  | | 42.6 | |  | |  | | % | 42.0 - 52.0 |
| HB | 14.3 |  | | 13.7 | |  | | 14.5 | |  | |  | | g/dl | 14.0 - 18.0 |
| MCH | 32.4 |  | | 32.1 | |  | | 31.3 | |  | |  | | pg | 27.0 - 34.0 |
| MCHC | 35.3 |  | | 35.9 | |  | | 34.0 | |  | |  | | g/dl | 32.0 - 36 |
| MCV | 91.7 |  | | 89.5 | |  | | 91.8 | |  | |  | | fl | 80 - 93 |
| Ery. Verteilungsbreite (RDW) | 13.0 |  | | 12.8 | |  | | 12.8 | |  | |  | | % | max. 15.0 |
| Thrombozyten | 166 |  | | 190 | |  | | 225 | |  | |  | | Tausd/µl | 150 - 450 |
| Verteilungsbreite der Thrombozyten | 11.2 |  | | 12.0 | |  | | 11.3 | |  | |  | | fl | 10 - 16 |
| Mittleres Plättchenvolumen | 10.0 |  | | 10.0 | |  | | 9.7 | |  | |  | | fl | 9 - 12 |
| **Gerinnung: Citrat-Blut** | | | | | | | | | | | | | | | |
| Quick |  |  | |  | |  | | 92 | |  | |  | | % | 70 - 120 |
| INR |  |  | |  | |  | | 1.0 | |  | |  | |  |  |
| **Elektrolyte: Li-Heparin-Plasma** | | | | | | | | | | | | | | | |
| Natrium | 136 |  | | 137 | |  | | 133 | |  | |  | | mmol/l | 136 - 148 |
| Kalium | 3.6 |  | | 4.1 | |  | | 4.3 | |  | |  | | mmol/l | 3.5 - 4.8 |
| **Substrate: Li-Heparin-Plasma** | | | | | | | | | | | | | | | |
| Kreatinin (enzymatisch) | 0.9 |  | | 0.8 | |  | | 1.0 | |  | |  | | mg/dl | 0.6 - 1.1 |
| GFR - MDRD | 81 |  | | 93 | |  | | 72 | |  | |  | | ml/min/ 1,73m2 | min. > 60 |
| GFR - CKD-EPI | 81 |  | | 85 | |  | | 71 | |  | |  | | ml/min/ 1,73m2 |  |
| Harnstoff | 23 |  | | 22 | |  | | 36 | |  | |  | | mg/dl | 12 - 46 |
| Bilirubin, gesamt |  |  | |  | |  | | 1,1 | |  | |  | | mg/dl | max. 1.1 |
| Cholesterin |  |  | |  | |  | | 295 | |  | |  | | mg/dl | 130 - 190 |
| HDL-Cholesterin |  |  | |  | |  | | 35 | |  | |  | | mg/dl | min. 35 |
| LDL-Cholesterin |  |  | |  | |  | | 192 | |  | |  | | mg/dl | max. 160 |
| Triglyzeride |  |  | |  | |  | | 306 | |  | |  | | mg/dl | max. 200 |
| C-reaktives Protein (CRP) | <0.05 |  | | <0.05 | |  | | <0.05 | |  | |  | | mg/dl | max. 0.50 |
| **Enzyme: Li-Heparin-Plasma** | | | | | | | | | | | | | | | |
| GOT/AST |  |  | |  | |  | | 37 | |  | |  | | U/l | max. 50 |
| Alkalische Phosphatase (AP) |  |  | |  | |  | | 97 | |  | |  | | U/l | 40 - 130 |
| Gamma-Glutamyl-Transferase (GGT) |  |  | |  | |  | | 55 | |  | |  | | U/l | max. 60 |
| **Sonstiges: Li-Heparin-Plasma** | | | | | | | | | | | | | | | |
| Osmolalität |  |  | |  | |  | | 298 | |  | |  | | mOsm/kg | 275 - 300 |
| **Hormone: Serum** | | | | | | | | | | | | | | | |
| TSH |  |  | |  | |  | | 3.09 | |  | |  | | mU/l | 0.5 - 4.4 |
| C-Peptid |  |  | |  | |  | | 1166 | |  | |  | | pmol/l | 140 - 830 |
| GAD-Antikörper | Neg. |  | |  | |  | |  | |  | |  | |  |  |
| IA2 - Antikörper | Neg. |  | |  | |  | |  | |  | |  | |  |  |
| **Laktat, Glukose: Na-F-Plasma** | | | | | | | | | | | | | | | |
| Glukose venös |  |  | |  | |  | | 430 | |  | |  | | mg/dl | 70 - 99 |
| **Sonstiges: EDTA-Blut** | | | | | | | | | | | | | | | |
| HbA1c (HPLC) |  |  | |  | |  | | 130.4 | |  | |  | | mmol/mol | 26.0 - 44.0 |
| HbA1c (glykiertes Hämoglobin) |  |  | |  | |  | | 14.1 | |  | |  | | % | 4.5 - 6.2 |
| **Urinstatus: Spontanurin** | | | | | | | | | | | | | | | |
| Urinstatus |  |  | |  | | Urinstatus | | Urinstatus | |  | |  | |  |  |
| Farbe des Spontanurin |  |  | |  | | Hellgelb | | Strohfarben | |  | |  | |  |  |
| Trübung im Spontanurin |  |  | |  | | klar | | klar | |  | |  | |  |  |
| Spez.Gewicht im Spontanurin |  |  | |  | | 1.029 | | 1.030 | |  | |  | | g/ml |  |
| pH-Wert im Spontanurin |  |  | |  | | 5.0 | | 5.0 | |  | |  | |  |  |
| Eiw.Qual. im Spontanurin |  |  | |  | | N. | | N. | |  | |  | |  |  |
| Gluk.Qual. im Spontanurin |  |  | |  | | ++++ | | ++++ | |  | |  | |  |  |
| U-Keton im Spontanurin |  |  | |  | | N. | | N. | |  | |  | |  |  |
| Hb Qual. im Spontanurin |  |  | |  | | N. | | N. | |  | |  | |  |  |
| Urobilinogen im Spontanurin |  |  | |  | | N. | | N. | |  | |  | |  |  |
| Bilirubin im Spontanurin |  |  | |  | | N. | | N. | |  | |  | |  |  |
| Leukos im Spontanurin |  |  | |  | | N. | | N. | |  | |  | |  |  |
| Nitrit im Spontanurin |  |  | |  | | N. | | N. | |  | |  | |  |  |
| **Sonstiges: Spontanurin** | | | | | | | | | | | | | | | |
| Kreatinin Konz. im Spontanurin |  | 41 | |  | |  | | 64 | |  | |  | | mg/dl |  |
| Eiweiss-Konz. im Spontanurin |  | <0.06 | |  | |  | | <0.06 | |  | |  | | g/l | max. 0.10 |
| Albumin Konzentration |  | <11 | |  | |  | | <11 | |  | |  | | mg/l | max. 20 |

**c) Diabetic nephropathy**

|  |  | |
| --- | --- | --- |
|  | | **University hospital** Department of Internal Medicine IV  Diabetology  Endocrinology  Nephrology  Medical Director  Prof. Konrad Kaiser, MD  Otfried-Müller-Straße 10  72076 Tübingen  Service telephone: 07071 29- 123456  Fax: 07071 29- 23456  Contact person:  Leon Link, MD |
| University hospital▪ Otfried-Müller-Str. 10 ▪ 72076 Tübingen | |  |
| Mr  Anton Arzt, MD  General practitioner  Arztweg 2  72070 Tübingen | |  |
|  | |  |
|  | |  |

Patient: **Marion** **Mason,** date of birth: **09.11.1969** Patient case no. 1 **23456789**

Address: **Teststraße 120, 72766 Reutlingen**

**Ward 75:** Admission **04.10.2023** / Discharge **08.10.2023** Please have ready in case of queries

Dear colleague,

We report on the inpatient stay of the above-mentioned patient on our ward 75.

**Diagnosis:**

| 1. | **Chronic kidney disease (CKD) stage G3b A3 with diabetic/hypertensive nephropathy**   - Laboratory:   10/23: creatinine 1.6 mg/dl, eGFR 32 ml/min/1.73m², increasing proteinuria to 2.5 g/g creatinine, microhematuria   - 06/23: creatinine 1.3 mg/dl, eGFR 48 ml/min/1.73m², no albuminuria, a1-microglobulinuria 11.5 mg/g creatinine - Kidney biopsy from 05/10/2023: Diabetic-hypertensive nephropathy, IFTA 40% - Renal secondary diseases: Art. Hypertension |
| --- | --- |
| 2. | Diabetes mellitus type 2, initial diagnosis 2017   - Current (10/23): HbA1c 8.2 % - 01/21: HbA1c 5.9% - Therapy: Metformin, SGLT2 inhibitor (new) |
| 3. | Obesity II°   - Current (10/23): BMI 35.62 kg/m² - Height 1.65 m, weight 97 kg |
| 4. | Arterial hypertension, initial diagnosis 2017 |
| 5. | Right knee TEP 05/2019 for joint arthrosis after skiing accident   - Regular intake of ibuprofen over 5-6 years |

**Current therapy recommendation:**

| Ramipril/amlodipine 5 mg/5 mg | 2 - 0 - 0, INCREASED, was 1 - 0 - 0 |
| --- | --- |
| HCT 12.5 mg | 1 - 0 - 0 |
| Metformin 500 mg | 1 - 0 - 1, REDUCED, was 2 - 0 - 2 |
| Forxiga 10 mg | 1 - 0 - 0, NEW |
| Torasemide 20 mg | 1 - 0 - 0, reduce gradually |
| Atorvastatin 40mg | 0 - 0 - 1 |
| Vigantoletten 1000IE | 1 - 0 - 0 |

**Further procedure:**

- We recommend an outpatient nephrology appointment to monitor the progress of CKD, initially every three months.

**Laboratory/urinalysis:**

See table appendix

**Medical history:**

M. Mason was admitted via our nephrology outpatient clinic for a kidney biopsy for chronic renal insufficiency and now increasing proteinuria and progressive deterioration in kidney function. There is a reduced general condition with reduced exercise tolerance. No AP complaints, occasional exertional dyspnea. Recently there has been increased fatigue. The blood pressure is around 150/80 mmHg in the home measurements, there are occasional hypertensive episodes with blood pressure values around 170/90 mmHg. M. Mason also reported occasional knee pain, for which he took ibuprofen. No B symptoms. Urine excretion was conspicuous with foaming, there was no hematuria or dysuria. The amount drunk was approx. 2 liters per day. The weight had recently increased to 97 kg. There is no nicotine consumption. No alcohol consumption. There is a penicillin allergy (CAVE: exanthema, shortness of breath), and the patient also reports an intolerance to GLP1 analogues (severe gastrointestinal side effects).

**Physical examination:**

Vital signs: HR: 85/min, RR: 154/96 mmHg, Temp: 36.5° C, AF: 20.0/min (04.10.2023 09:04)

Patient condition: obese DC, good GC

Vigilance awake, orientated to time, place, person, situation

Lungs: VBSs with limited examination conditions

Heart: heart sounds pure, rhythmic; no heart murmurs

Abdomen: bowel sounds lively in all 4 quadrants, abdominal wall soft, no pressure/loosening pain, no resistance

Extremities: Lower leg edema on both sides

**Kidney biopsy from 04/10/2023:**

Light microscopy: Histologically, the material processed in sections and special stains shows renal tissue of the cortical-medullary region with a total of 18 glomerulus sections, 8 of which are globally obliterated. Nodular matrix proliferation in the glomeruli, pronounced hyalinosis in the arterioles. No granulomas, no tissue eosinophilia. No inflammatory vascular changes, no fresh or older microthrombi.

Immunohistochemistry: IgA, IgG, IgM, C1q and C3c: no deposits of immune complexes

Assessment: Renal punch cylinder with thickening of the glomerular basement membrane, proliferation of the mesangial cells and matrix formation with nodule-forming glomerulosclerosis, pronounced vessel wall hyalinosis, approx. 40% tubule atrophy and interstitial fibrosis with at least moderate arterial arteriolosclerosis. Overall findings compatible with diabetic-hypertensive nephropathy.

**Summarised assessment:**

M. Mason was admitted to our nephrology ward for a kidney biopsy for chronic renal insufficiency stage CKD G3bA3 and now increasing proteinuria.

On admission, Mrs Mason was in good general health and free of symptoms. The physical examination revealed lower leg edema on both sides. Laboratory chemistry showed a progressive deterioration in renal function with a plasma creatinine of 1.6 mg/dl corresponding to an estimated GFR CKD-EPI of 32 ml/min/1.73 m² (previous value 06/2023: 1.3 mg/dl) and increasing proteinuria of 2.5 g/g creatinine. After exclusion of contraindications, with a normal coagulation situation and after informed consent, a renal biopsy of the right kidney was performed on 4 October 2023, primarily without complications. The post-interventional course was problem-free and without clinical, laboratory or sonographic evidence of post-operative hemorrhage.

Nephropathological examination of the biopsy material revealed nodular glomerulosclerosis and arteriolar hyalinosis, consistent with diabetic-hypertensive nephropathy. The interstitial tubular atrophy and fibrosis was 40%. Therapeutically, conservative measures to inhibit progression such as SGLT2 inhibition, RAAS blockade and optimization of diabetes mellitus and arterial hypertension are therefore in the foreground.

In terms of blood glucose, the HbA1c under metformin therapy and dietary restriction is not yet adequately controlled at 8.2 %. Initially, the dose of metformin was adjusted to the kidney function. With regard to further therapeutic options, there is an intolerance to GLP1 analogues. We therefore started treatment with an SGLT2 inhibitor to optimize blood glucose levels and to inhibit the progression of renal insufficiency, as described above. Should an infectious disease with fever occur, therapy with the SGLT2 inhibitor, as well as therapy with metformin, should be paused due to the increased risk of keto or lactic acidosis ("sick day rules"). Please ensure increased vigilance due to the patient's penicillin allergy when presenting with an infection to the doctor.

In terms of blood pressure, M. Mason reported recurrently elevated blood pressure values. Ramipril/amlodipine was increased as described above, and the blood pressure values on the ward improved to around 140 mmHg systolic. We recommend regular blood pressure measurements at home to further optimize the antihypertensive therapy during the course of treatment. In the case of lower leg edema and weight gain, diuretic therapy with torasemide for volume reduction was also started. After reaching an euvolemic state, the dose should be gradually reduced to avoid prerenal kidney damage. For antiproteinuric therapy, the patient is now receiving the SGLT2 inhibitor and the ACE inhibitor in increased doses.

In addition, in the case of grade II obesity, weight reduction with a target BMI of <30 kg/m^2^ should be aimed for, both for nephroprotection (reduction of hyperfiltration) and to optimize blood glucose levels. We recommend adherence to a Mediterranean diet for this purpose. The use of nephrotoxic substances, especially non-steroidal anti-inflammatory drugs, should be avoided.

We discharged M. Mason on 8 October 2023 in a stable general condition for further outpatient treatment in accordance with the above procedure. If you have any questions, please do not hesitate to contact us.

With best regards

| Prof. Anton Arnold, MD  Head of the Nephrology Section | Karina Klein, MD  Senior Physician Nephrology | Leon Link, MD  Resident |
| --- | --- | --- |

**Laboratory**

| Destination | 05.10.23 | 04.10.23 | | 25.09.2023 | 26.06.23 | 26.06.23 | |  | |  | | Unit | Reference |  |
| --- | --- | --- | --- | --- | --- | --- | --- | --- | --- | --- | --- | --- | --- | --- |
| Order | 07:00 | 07:00 | |  | 14:31 | 14:08 | |  | |  | | | |  |
| Order number |  | |  |  |  | |  | |  | |  | | | |
| **(Diff) blood count: EDTA blood** | | | | | | | | | | | | | |  |
| Leukocytes | 9610 | 10490 | |  | 8950 |  | |  | |  | | 1/µl | 3800 - 10300 |  |
| Neutrophils abs |  | 5.90 | |  | 5.02 |  | |  | |  | | Thousand/µl | 1.80 - 7.00 |  |
| Lymphocytes abs |  | 3.69 | |  | 3.15 |  | |  | |  | | Thousand/µl | 1.1 - 3.2 |  |
| Monocytes abs |  | 0.67 | |  | 0.59 |  | |  | |  | | Thousand/µl | 0.26 - 0.87 |  |
| Eosinophil abs |  | 0.15 | |  | 0.07 |  | |  | |  | | Thousand/µl | 0.03 - 0.47 |  |
| Basophilic abs |  | 0.04 | |  | 0.05 |  | |  | |  | | Thousand/µl | 0.02 - 0.11 |  |
| Immature granulocytes abs |  | 0.04 | |  | 0.07 |  | |  | |  | | Thousand/µl | 0 - 0.06 |  |
| Neutrophils |  | 56.2 | |  | 56.0 |  | |  | |  | | % | 40.0 - 80.0 |  |
| Lymphocytes |  | 35.2 | |  | 35.2 |  | |  | |  | | % | 20.0 - 45.0 |  |
| Monocytes |  | 6.4 | |  | 6.6 |  | |  | |  | | % | 2.0 - 13 |  |
| Eosinophils |  | 1.4 | |  | 0.8 |  | |  | |  | | % | 0.5 - 8 |  |
| Basophile |  | 0.4 | |  | 0.6 |  | |  | |  | | % | max. 2.0 |  |
| Immature granulocytes |  | 0.4 | |  | 0.8 |  | |  | |  | | % | 0 - 0.6 |  |
| Normoblasts abs |  | 0 | |  | 0 |  | |  | |  | | Thousand/µl | 0 - 0.1 |  |
| Normoblasts |  | 0 | |  | 0 |  | |  | |  | | % | 0 - 1 |  |
|  | | | | | | | | | | | | | |  |
| Erythrocytes |  | 5.36 | |  | 5.67 |  | |  | |  | | Mio/µl | 4.20 - 6.20 |  |
| Haematocrit |  | 45.0 | |  | 46.9 |  | |  | |  | | % | 42.0 - 52.0 |  |
| HB | 12.4 | 12.6 | |  | 12.3 |  | |  | |  | | g/dl | 14.0 - 18.0 |  |
| MCH | 29 | 29.1 | |  | 28.7 |  | |  | |  | | pg | 27.0 - 34.0 |  |
| MCHC | 33.1 | 34.7 | |  | 34.8 |  | |  | |  | | g/dl | 32.0 - 36 |  |
| MCV | 83.5 | 84.0 | |  | 82.7 |  | |  | |  | | fl | 80 - 93 |  |
| Ery. Distribution width (RDW) | 13.2 | 13.0 | |  | 12.9 |  | |  | |  | | % | max. 15.0 |  |
| Thrombocytes | 202 | 215 | |  | 224 |  | |  | |  | | Thousand/µl | 150 - 450 |  |
| Distribution range of the thrombocytes | 11.5 | 11.7 | |  | 13.0 |  | |  | |  | | fl | 10 - 16 |  |
| Medium platelet volume | 11 | 10.3 | |  | 10.8 |  | |  | |  | | fl | 9 - 12 |  |
| **Coagulation: citrated blood** | | | | | | | | | | | | | |  |
| Quick |  | >120 | |  |  |  | |  | |  | | % | 70 - 120 |  |
| INR |  | 0.9 | |  |  |  | |  | |  | |  |  |  |
| **Electrolytes: Li-heparin plasma** | | | | | | | | | | | | | |  |
| Sodium |  | 142 | |  | 136 |  | |  | |  | | mmol/l | 136 - 148 |  |
| Potassium |  | 4.8 | |  | 4.3 |  | |  | |  | | mmol/l | 3.5 - 4.8 |  |
| Calcium |  | 2.2 | |  | 2.4 |  | |  | |  | | mmol/l | 2.1 - 2.6 |  |
| Inorganic phosphate |  | 1.5 | |  |  |  | |  | |  | | mmol/l | 0.8 - 1.5 |  |
| **Substrates: Li-heparin plasma** | | | | | | | | | | | | | |  |
| Creatinine (enzymatic) |  | 1.6 | |  | 1.3 |  | |  | |  | | mg/dl | 0.6 - 1.1 |  |
| GFR - CKD-EPI |  | 32 | |  | 48 |  | |  | |  | | ml/min/ 1,73m2 |  |  |
| Urea |  | 83 | |  | 64 |  | |  | |  | | mg/dl | 12 - 46 |  |
| Uric acid |  | 6.3 | |  |  |  | |  | |  | | mg/dl | 3.4 - 7.0 |  |
| Total egg white |  | 7.7 | |  |  |  | |  | |  | | g/dl | 6.5 - 8.5 |  |
| Cholesterol |  | 183 | |  | 259 |  | |  | |  | | mg/dl | 130 - 190 |  |
| HDL cholesterol |  | 45 | |  | 47 |  | |  | |  | | mg/dl | min. 35 |  |
| LDL cholesterol |  | 67 | |  | 152 |  | |  | |  | | mg/dl | max. 160 |  |
| Triglycerides |  | 240 | |  | 372 |  | |  | |  | | mg/dl | max. 200 |  |
| C-reactive protein (CRP) |  | 0.4 | |  |  |  | |  | |  | | mg/dl | max. 0.50 |  |
| **Enzymes: Li-heparin plasma** | | | | | | | | | | | | | |  |
| Creatine kinase (CK) |  | 120 | |  | 100 |  | |  | |  | | U/l | max. 190 |  |
| GOT/AST |  | <8 | |  |  |  | |  | |  | | U/l | max. 50 |  |
| GPT/ALT |  | 22 | |  | 26 |  | |  | |  | | U/l | max. 50 |  |
| Alkaline phosphatase (AP) |  | 100 | |  |  |  | |  | |  | | U/l | 40 - 130 |  |
| Lactate dehydrogenase (LDH) |  | 148 | |  |  |  | |  | |  | | U/l | max. 250 |  |
| Gamma-glutamyl transferase (GGT) |  | 49 | |  |  |  | |  | |  | | U/l | max. 60 |  |
| Lipase |  | 41 | |  |  |  | |  | |  | | U/l | max. 60 |  |
| **Serum proteins: Serum** | | | | | | | | | | | | | |  |
| Lipoprotein (a) ( Lp(a) ) |  |  | |  | <10 |  | |  | |  | | mg/dl | max. 30 |  |
| **Hormones: Li-heparin plasma** | | | | | | | | | | | | | |  |
| TSH |  |  | |  | 2.08 |  | |  | |  | | mU/l | 0.5 - 4.4 |  |
| **Hormones: Serum** | | | | | | | | | | | | | |  |
| Free thyroxine (FT4) |  | 13 | |  |  |  | |  | |  | | pmol/l | 11 - 23 |  |
| Free triiodothyronine (FT3) |  | 5.5 | |  |  |  | |  | |  | | pmol/l | 3.5 - 6.5 |  |
| TSH |  | 2.78 | |  |  |  | |  | |  | | mU/l | 0.5 - 4.4 |  |
| **Lactate, glucose: Na-F plasma** | | | | | | | | | | | | | |  |
| Venous glucose |  | 157 | |  | 161 |  | |  | |  | | mg/dl | 70 - 99 |  |
| **Other: EDTA blood** | | | | | | | | | | | | | |  |
| HbA1c (glycated haemoglobin) |  | 8.1 | |  | 7.5 |  | |  | |  | | % | 4.5 - 6.2 |  |
| **Urine status: Spontaneous urine** | | | | | | | | | | | | | |  |
| Urine status |  | Urine status | | Urine status |  | Urine status | |  | |  | |  |  |  |
| Colour of the spontaneous urine |  | Straw colours | | Straw colours |  | Straw colours | |  | |  | |  |  |  |
| Turbidity in spontaneous urine |  | clear | | clear |  | clear | |  | |  | |  |  |  |
| Specific gravity in spontaneous urine |  | 1.012 | | 1.014 |  | 1.011 | |  | |  | | g/ml |  |  |
| pH value in spontaneous urine |  | 5.0 | | 5.2 |  | 5.5 | |  | |  | |  |  |  |
| Protein quality in spontaneous urine |  | +++ | | +++ |  | N. | |  | |  | |  |  |  |
| Gluc.qual. in spontaneous urine |  | + | | + |  | N. | |  | |  | |  |  |  |
| U-ketone in spontaneous urine |  | N. | | N. |  | N. | |  | |  | |  |  |  |
| Hb qual. in spontaneous urine |  | + | | + |  | N. | |  | |  | |  |  |  |
| Urobilinogen in spontaneous urine |  | N. | | N. |  | N. | |  | |  | |  |  |  |
| Bilirubin in spontaneous urine |  | N. | | N. |  | N. | |  | |  | |  |  |  |
| Leukocytes in spontaneous urine |  | N. | | N. |  | N. | |  | |  | |  |  |  |
| Nitrite in spontaneous urine |  | N. | | N. |  | N. | |  | |  | |  |  |  |
| **Other: Spontaneous urine** | | | | | | | | | | | | | |  |
| Creatinine conc. in spontaneous urine |  | 72 | |  |  | 67 | |  | |  | | mg/dl |  |  |
| Protein concentration in spontaneous urine |  | 2.5 | | 2.2 |  | <0.06 | |  | |  | | g/g Crea | max. 0.3 |  |
| Albumin concentration |  | 1705 | | 1530 |  | 19 | |  | |  | | mg/l | max. 20 |  |
| Quot. U-albumin /g Crea |  | 2240 | | 1810 |  | 11,5 | |  | |  | | mg/g Crea | max. 20 |  |

|  |  | |
| --- | --- | --- |
|  | | **Medizinische Klinik** Abteilung Innere Medizin IV  Diabetologie  Endokrinologie  Nephrologie  Ärztlicher Direktor  Prof. Dr. med. Konrad Kaiser  Otfried-Müller-Straße 10  72076 Tübingen  Servicetelefon: 07071 29-123456  Fax: 07071 29-23456  Ansprechpartner:  Herr Dr. Leon Link |
| Medizinische Klinik ▪ Otfried-Müller-Str. 10 ▪ 72076 Tübingen | |  |
| Herrn  Dr. med. Anton Arzt  FA für Allgemeinmedizin  Arztweg 2  72070 Tübingen | |  |
|  | |  |
|  | |  |

Patient: **Marion Maurer,** Geb.-Datum: **09.11.1969** Patienten-Fall-Nr. **123456789**

wohnhaft: **Teststraße 120, 72766** **Reutlingen**

**Station 75:** Aufnahme **04.10.2023** / Entlassung **08.10.2023** bitte bei Rückfragen bereithalten

Sehr geehrter Herr Kollege,

wir berichten über den stationären Aufenthalt des/r oben genannten Patienten/in auf unserer Station 75.

**Diagnose:**

| 1. | **Chronische Nierenkrankheit (CKD) Stadium G3b A3 bei diabetisch/hypertensiver Nephropathie**   - Labor:   10/23: Kreatinin 1,6 mg/dl, eGFR 32 ml/min/1,73m², steigende Proteinurie auf 2,5 g/g Kreatinin, Mikrohämaturie   - 06/23: Kreatinin 1,3 mg/dl, eGFR 48 ml/min/1,73m², Keine Albuminurie, a1-Mikroglobulinurie 11,5 mg/g Kreatinin - Nierenbiopsie vom 05.10.2023: Diabetisch-hypertensive Nephropathie, IFTA 40% - Renale Folgeerkrankungen: Art. Hypertonie |
| --- | --- |
| 2. | Diabetes mellitus Typ 2, ED 2017   - Aktuell (10/23): HbA1c 8,2 % - 01/21: HbA1c 5,9% - Therapie: Metformin, SGLT2 Hemmer (neu) |
| 3. | Adipositas II°   - Aktuell (10/23): BMI 35,62 kg/m² - Größe 1,65 m, Gewicht 97 kg |
| 4. | Arterielle Hypertonie, ED 2017 |
| 5. | Z.n. Knie TEP rechts 05/2019 bei Gelenksarthrose nach Skiunfall   - Regelmäßige Ibuprofen-Einnahme über 5-6 Jahre |

**Aktuelle Therapieempfehlung:**

| Ramipril/Amlodipin 5 mg/5 mg | 2 – 0 – 0, GESTEIGERT, war 1 – 0 – 0 |
| --- | --- |
| HCT 12,5 mg | 1 – 0 – 0 |
| Metformin 500 mg | 1 – 0 – 1, REDUZIERT, war 2 – 0 – 2 |
| Forxiga 10 mg | 1 – 0 – 0, NEU |
| Torasemid 20 mg | 1 – 0 – 0, im Verlauf reduzieren |
| Atorvastatin 40mg | 0 – 0 – 1 |
| Vigantoletten 1000IE | 1 – 0 – 0 |

**Weiteres Procedere:**

- Wir empfehlen die ambulante nephrologische Anbindung zur Verlaufskontrolle der CKD im Intervall von zunächst allen drei Monaten.

**Labor/Urinanalytik:**

Siehe Tabellenanhang

**Anamnese:**

Die Einweisung von M. Maurer erfolgte über unsere nephrologische Ambulanz zur Nierenbiopsie bei chronischer Niereninsuffizienz und nun steigender Proteinurie und progredienter Nierenfunktionsverschlechterung. Es besteht ein reduziertes Allgemeinbefinden mit reduzierter Belastbarkeit. Keine AP Beschwerden, gelegentliche Belastungsdyspnoe. In letzter Zeit bestehe eine vermehrte Müdigkeit. Der Blutdruck sei in den häuslichen Messungen um 150/80 mmHg, es bestehen gelegentliche hypertensive Episoden mit Blutdruckwerten um 170/90 mmHg. Zudem berichtet M. Maurer über gelegentliche Knieschmerzen, für die Ibuprofen eingenommen werde. Keine B-Symptomatik. Die Urinausscheidung sei auffällig mit Schäumen, es besteht keine Hämaturie oder Dysurie. Die Trinkmenge liege bei ca. 2 l pro Tag. Das Gewicht habe zuletzt zugenommen auf 97 kg. Es bestehe kein Nikotinkonsum. Kein Alkoholkonsum. Es besteht eine Penicillinallergie (CAVE: Exanthem, Luftnot), zudem berichtet die Patientin von einer Unverträglichkeit gegenüber GLP1 Analoga (starke gastrointestinale Nebenwirkungen).

**Körperliche Untersuchung:**

Vitalparameter: HF: 85/min, RR: 154/96 mmHg, Temp: 36,5° C, AF: 20.0/min (04.10.2023 09:04)

Patientenzustand: Adipöser EZ, guter AZ

Vigilanz wach, orientiert zu Zeit, Ort, Person, Situation

Lunge: VAGs bei eingeschränkten Untersuchungsbedingungen

Herz: Herztöne rein, rhythmisch; keine Herzgeräusche

Abdomen: Darmgeräusche in allen 4 Quadranten lebhaft, Bauchdecken weich, kein Druck-/Loslassschmerz, keine Resistenzen

Extremitäten: Unterschenkelödeme beidseits

**Nierenbiopsie vom 04.10.2023:**

Lichtmikroskopie: Histologisch zeigt das in Schnittstufen und Spezialfärbungen aufgearbeitete Material Nierengewebe der Rinden-Markregion mit insgesamt 18 Glomerulusanschnitten, von denen 8 global obliteriert sind. Noduläre Matrixvermehrung in den Glomeruli, ausgeprägte Hyalinose in den Arteriolen. Keine Granulome, keine Gewebseosinophilie. Keine entzündlichen Gefäßveränderungen, keine frischen oder älteren Mikrothromben.

Immunhistochemie: IgA, IgG, IgM, C1q und C3c: keine Ablagerungen von Immunkomplexen

Begutachtung: Nierenstanzzylinder mit einer Verdickung der glomerulären Basalmembran, Proliferation der mesangialen Zellen und Matrixbildung mit nodulbildender Glomerulosklerose, ausgeprägter Gefäßwandhyalinose, ca. 40%ige Tubulusatrophie und interstitieller Fibrose mit mindestens mittelschwerer arterieller-Arteriolosklerose. Befund insgesamt vereinbar mit einer diabetisch-hypertensiven Nephropathie.

**Zusammenfassende Beurteilung:**

Die Vorstellung von M. Maurer auf unserer nephrologischen Station erfolgte zur Nierenbiopsie bei chronischer Niereninsuffizienz Stadium CKD G3bA3 und nun zunehmender Proteinurie.

Bei Aufnahme zeigte sich M. Maurer in regelrechtem Allgemeinzustand und beschwerdefrei. In der körperlichen Untersuchung zeigten sich Unterschenkelödeme beidseits. Laborchemisch bestand eine progrediente Nierenfunktionsverschlechterung mit einem Plasma Kreatinin von 1,6 mg/dl entsprechend einer geschätzten GFR CKD-EPI von 32 ml/min/1,73 m² (Vorwert 06/2023: 1,3 mg/dl) sowie einer zunehmenden Proteinurie von 2,5 g/g Kreatinin. Nach Ausschluss von Kontraindikationen, bei normwertiger Gerinnungssituation und bei vorliegender Aufklärung und Einverständnis erfolgte am 04.10.2023 die primär komplikationslose Nierenbiopsie der rechten Niere. Der postinterventionelle Verlauf gestaltete sich problemlos und ohne klinischen, laborchemischen oder sonographischen Hinweis auf eine Nachblutung.

In der nephropathologischen Aufarbeitung des Biopsiematerials zeigte sich eine noduläre Glomerulosklerose sowie eine arterioläre Hyalinose, passend zu einer diabetisch-hypertensiven Nephropathie. Die interstitielle Tubulusatrophie und Fibrose lag bei 40%. Therapeutisch stehen somit konservative Maßnahmen der Progressionshemmung wie SGLT2-Inhibition, RAAS-Blockade und Optimierung des Diabetes mellitus sowie der arteriellen Hypertonie im Vordergrund.

Von Seiten des Blutzuckers ist der HbA1c unter Metformin Therapie und diätischer Restriktion mit 8,2 % noch nicht adäquat eingestellt. Es erfolgte zunächst eine Dosisadaption von Metformin an die Nierenfunktion. In Bezug auf weitere therapeutische Optionen besteht eine Unverträglichkeit gegenüber GLP1 Analoga. Wir begannen daher wie oben angegeben eine Therapie mit einem SGLT2 Hemmer zur Blutzuckeroptimierung sowie zur Progressionshemmung der Niereninsuffizienz. Sollte es zu einer Infektionserkrankung mit Fieber kommen, ist die Therapie mit dem SGLT 2 Hemmer, ebenso wie die Therapie mit Metformin, aufgrund des erhöhten Risikos für eine Keto- bzw. Laktatazidose zu pausieren („Sick Day Rules“). Bitte im Infekt bei ärztlicher Vorstellung erhöhte Vigilanz bei Penicillinallergie.

Von Seiten des Blutdrucks berichtet M. Maurer über rezidivierend erhöhte Blutdruckwerte. Es erfolgte die Steigerung von Ramipril/Amlodipin wie oben angegeben, darunter zeigten sich die Blutdruckwerte auf Station gebessert mit Werten um 140 mmHg systolisch. Wir empfehlen regelmäßige häusliche Blutdruckmessungen zur weiteren Optimierung der antihypertensiven Therapie im Verlauf. Bei Unterschenkelödemen sowie Gewichtszunahme erfolgte zudem der Beginn einer diuretischen Therapie mit Torasemid zur Volumenrekompensation. Hier sollte nach Erreichen der Euvolämie eine schrittweise Dosisreduktion zur Vermeidung einer prärenalen Nierenschädigung erfolgen. Zur antiproteinurischen Therapie erhält die Patientin nun den SGLT2 Hemmer sowie den ACE Hemmer in gesteigerter Dosierung.

Zudem ist bei Adipositas Grad II sowohl zur Nephroprotektion (Reduktion der Hyperfiltration) als auch zur Blutzuckeroptimierung eine Gewichtsreduktion mit Erreichen eines Ziel-BMI von <30 kg/m^2^ anzustreben. Wir empfehlen hierzu die Einhaltung einer mediterranen Diät. Der Gebrauch von nephrotoxischen Substanzen, insbesondere von nicht steroidalen Antirheumatika, sollte vermieden werden.

Wir entlassen M. Maurer am 08.10.2023 unter Beachtung des obenstehenden Procederes in stabilem Allgemeinzustand in Ihre ambulante Weiterbehandlung. Bei Rückfragen stehen wir Ihnen gerne zur Verfügung.

Mit freundlichen kollegialen Grüßen

| Prof. Dr. med. Anton Arnold Leiter Sektion Nephrologie | Dr. med. Karina Klein  Oberärztin Nephrologie | Dr. Leon Link  Assistenzarzt |
| --- | --- | --- |

**Labor**

| Bestimmung | 05.10.23 | 04.10.23 | | 25.09.2023 | 26.06.23 | 26.06.23 | |  | |  | | Einheit | Referenz |  |
| --- | --- | --- | --- | --- | --- | --- | --- | --- | --- | --- | --- | --- | --- | --- |
| Auftrag | 07:00 | 07:00 | |  | 14:31 | 14:08 | |  | |  | | | |  |
| Auftrag-Nummer |  | |  |  |  | |  | |  | |  | | | |
| **(Diff)-Blutbild: EDTA-Blut** | | | | | | | | | | | | | |  |
| Leukozyten | 9610 | 10490 | |  | 8950 |  | |  | |  | | 1/µl | 3800 - 10300 |  |
| Neutrophile abs |  | 5.90 | |  | 5.02 |  | |  | |  | | Tausd/µl | 1.80 - 7.00 |  |
| Lymphozyten abs |  | 3.69 | |  | 3.15 |  | |  | |  | | Tausd/µl | 1.1 - 3.2 |  |
| Monozyten abs |  | 0.67 | |  | 0.59 |  | |  | |  | | Tausd/µl | 0.26 - 0.87 |  |
| Eosinophile abs |  | 0.15 | |  | 0.07 |  | |  | |  | | Tausd/µl | 0.03 - 0.47 |  |
| Basophile abs |  | 0.04 | |  | 0.05 |  | |  | |  | | Tausd/µl | 0.02 - 0.11 |  |
| Unreife Granulozyten abs |  | 0.04 | |  | 0.07 |  | |  | |  | | Tausd/µl | 0 - 0.06 |  |
| Neutrophile |  | 56.2 | |  | 56.0 |  | |  | |  | | % | 40.0 - 80.0 |  |
| Lymphozyten |  | 35.2 | |  | 35.2 |  | |  | |  | | % | 20.0 - 45.0 |  |
| Monozyten |  | 6.4 | |  | 6.6 |  | |  | |  | | % | 2.0 - 13 |  |
| Eosinophile |  | 1.4 | |  | 0.8 |  | |  | |  | | % | 0.5 - 8 |  |
| Basophile |  | 0.4 | |  | 0.6 |  | |  | |  | | % | max. 2.0 |  |
| Unreife Granulozyten |  | 0.4 | |  | 0.8 |  | |  | |  | | % | 0 - 0.6 |  |
| Normoblasten abs |  | 0 | |  | 0 |  | |  | |  | | Tausd/µl | 0 - 0.1 |  |
| Normoblasten |  | 0 | |  | 0 |  | |  | |  | | % | 0 - 1 |  |
|  | | | | | | | | | | | | | |  |
| Erythrozyten |  | 5.36 | |  | 5.67 |  | |  | |  | | Mio/µl | 4.20 - 6.20 |  |
| Haematokrit |  | 45.0 | |  | 46.9 |  | |  | |  | | % | 42.0 - 52.0 |  |
| HB | 12.4 | 12.6 | |  | 12.3 |  | |  | |  | | g/dl | 14.0 - 18.0 |  |
| MCH | 29 | 29.1 | |  | 28.7 |  | |  | |  | | pg | 27.0 - 34.0 |  |
| MCHC | 33.1 | 34.7 | |  | 34.8 |  | |  | |  | | g/dl | 32.0 - 36 |  |
| MCV | 83.5 | 84.0 | |  | 82.7 |  | |  | |  | | fl | 80 - 93 |  |
| Ery. Verteilungsbreite (RDW) | 13.2 | 13.0 | |  | 12.9 |  | |  | |  | | % | max. 15.0 |  |
| Thrombozyten | 202 | 215 | |  | 224 |  | |  | |  | | Tausd/µl | 150 - 450 |  |
| Verteilungsbreite der Thrombozyten | 11.5 | 11.7 | |  | 13.0 |  | |  | |  | | fl | 10 - 16 |  |
| Mittleres Plättchenvolumen | 11 | 10.3 | |  | 10.8 |  | |  | |  | | fl | 9 - 12 |  |
| **Gerinnung: Citrat-Blut** | | | | | | | | | | | | | |  |
| Quick |  | >120 | |  |  |  | |  | |  | | % | 70 - 120 |  |
| INR |  | 0.9 | |  |  |  | |  | |  | |  |  |  |
| **Elektrolyte: Li-Heparin-Plasma** | | | | | | | | | | | | | |  |
| Natrium |  | 142 | |  | 136 |  | |  | |  | | mmol/l | 136 - 148 |  |
| Kalium |  | 4.8 | |  | 4.3 |  | |  | |  | | mmol/l | 3.5 - 4.8 |  |
| Calcium |  | 2.2 | |  | 2.4 |  | |  | |  | | mmol/l | 2.1 - 2.6 |  |
| Anorganisches Phosphat |  | 1.5 | |  |  |  | |  | |  | | mmol/l | 0.8 - 1.5 |  |
| **Substrate: Li-Heparin-Plasma** | | | | | | | | | | | | | |  |
| Kreatinin (enzymatisch) |  | 1.6 | |  | 1.3 |  | |  | |  | | mg/dl | 0.6 - 1.1 |  |
| GFR - CKD-EPI |  | 32 | |  | 48 |  | |  | |  | | ml/min/ 1,73m2 |  |  |
| Harnstoff |  | 83 | |  | 64 |  | |  | |  | | mg/dl | 12 - 46 |  |
| Harnsäure |  | 6.3 | |  |  |  | |  | |  | | mg/dl | 3.4 - 7.0 |  |
| Gesamt-Eiweiss |  | 7.7 | |  |  |  | |  | |  | | g/dl | 6.5 - 8.5 |  |
| Cholesterin |  | 183 | |  | 259 |  | |  | |  | | mg/dl | 130 - 190 |  |
| HDL-Cholesterin |  | 45 | |  | 47 |  | |  | |  | | mg/dl | min. 35 |  |
| LDL-Cholesterin |  | 67 | |  | 152 |  | |  | |  | | mg/dl | max. 160 |  |
| Triglyzeride |  | 240 | |  | 372 |  | |  | |  | | mg/dl | max. 200 |  |
| C-reaktives Protein (CRP) |  | 0.4 | |  |  |  | |  | |  | | mg/dl | max. 0.50 |  |
| **Enzyme: Li-Heparin-Plasma** | | | | | | | | | | | | | |  |
| Creatinkinase (CK) |  | 120 | |  | 100 |  | |  | |  | | U/l | max. 190 |  |
| GOT/AST |  | <8 | |  |  |  | |  | |  | | U/l | max. 50 |  |
| GPT/ALT |  | 22 | |  | 26 |  | |  | |  | | U/l | max. 50 |  |
| Alkalische Phosphatase (AP) |  | 100 | |  |  |  | |  | |  | | U/l | 40 - 130 |  |
| Laktatdehydrogenase (LDH) |  | 148 | |  |  |  | |  | |  | | U/l | max. 250 |  |
| Gamma-Glutamyl-Transferase (GGT) |  | 49 | |  |  |  | |  | |  | | U/l | max. 60 |  |
| Lipase |  | 41 | |  |  |  | |  | |  | | U/l | max. 60 |  |
| **Serumproteine: Serum** | | | | | | | | | | | | | |  |
| Lipoprotein (a) ( Lp(a) ) |  |  | |  | <10 |  | |  | |  | | mg/dl | max. 30 |  |
| **Hormone: Li-Heparin-Plasma** | | | | | | | | | | | | | |  |
| TSH |  |  | |  | 2.08 |  | |  | |  | | mU/l | 0.5 - 4.4 |  |
| **Hormone: Serum** | | | | | | | | | | | | | |  |
| Freies Thyroxin (FT4) |  | 13 | |  |  |  | |  | |  | | pmol/l | 11 - 23 |  |
| Freies Trijodthyronin (FT3) |  | 5.5 | |  |  |  | |  | |  | | pmol/l | 3.5 - 6.5 |  |
| TSH |  | 2.78 | |  |  |  | |  | |  | | mU/l | 0.5 - 4.4 |  |
| **Laktat, Glukose: Na-F-Plasma** | | | | | | | | | | | | | |  |
| Glukose venös |  | 157 | |  | 161 |  | |  | |  | | mg/dl | 70 - 99 |  |
| **Sonstiges: EDTA-Blut** | | | | | | | | | | | | | |  |
| HbA1c (glykiertes Hämoglobin) |  | 8.1 | |  | 7.5 |  | |  | |  | | % | 4.5 - 6.2 |  |
| **Urinstatus: Spontanurin** | | | | | | | | | | | | | |  |
| Urinstatus |  | Urinstatus | | Urinstatus |  | Urinstatus | |  | |  | |  |  |  |
| Farbe des Spontanurin |  | Strohfarben | | Strohfarben |  | Strohfarben | |  | |  | |  |  |  |
| Trübung im Spontanurin |  | klar | | klar |  | klar | |  | |  | |  |  |  |
| Spez.Gewicht im Spontanurin |  | 1.012 | | 1.014 |  | 1.011 | |  | |  | | g/ml |  |  |
| pH-Wert im Spontanurin |  | 5.0 | | 5.2 |  | 5.5 | |  | |  | |  |  |  |
| Eiw.Qual. im Spontanurin |  | +++ | | +++ |  | N. | |  | |  | |  |  |  |
| Gluk.Qual. im Spontanurin |  | + | | + |  | N. | |  | |  | |  |  |  |
| U-Keton im Spontanurin |  | N. | | N. |  | N. | |  | |  | |  |  |  |
| Hb Qual. im Spontanurin |  | + | | + |  | N. | |  | |  | |  |  |  |
| Urobilinogen im Spontanurin |  | N. | | N. |  | N. | |  | |  | |  |  |  |
| Bilirubin im Spontanurin |  | N. | | N. |  | N. | |  | |  | |  |  |  |
| Leukos im Spontanurin |  | N. | | N. |  | N. | |  | |  | |  |  |  |
| Nitrit im Spontanurin |  | N. | | N. |  | N. | |  | |  | |  |  |  |
| **Sonstiges: Spontanurin** | | | | | | | | | | | | | |  |
| Kreatinin Konz. im Spontanurin |  | 72 | |  |  | 67 | |  | |  | | mg/dl |  |  |
| Eiweiss-Konz. im Spontanurin |  | 2.5 | | 2.2 |  | <0.06 | |  | |  | | g/g Krea | max. 0.3 |  |
| Albumin Konzentration |  | 1705 | | 1530 |  | 19 | |  | |  | | mg/l | max. 20 |  |
| Quot. U-Albumin /g Krea |  | 2240 | | 1810 |  | 11,5 | |  | |  | | mg/g Krea | max. 20 |  |

**Supplementary Material 2: Original Prompting (German version)**

Task 1: Structured Info

Im Folgenden gebe ich Ihnen meinen Arztbrief. Ich möchte, dass Sie den Arztbrief für mich in verständlicher Sprache auflisten. Mein Verständnis ist ungefähr auf der Stufe der achten Klasse. Bitte erklären Sie medizinische Abstufungen. Ich möchte, dass Sie Ihre Antwort im JSON Format als dict geben, und zwar mit folgender Struktur:\n ["Wann war die ärztliche Vorstellung? Wo war sie (Praxis, Klinik)? Wie war sie (ambulant oder stationär)?", "Hauptdiagnose","Nebendiagnosen"]\n Das war die Struktur, in der Sie bitte Ihre Antwort geben, hier ist jetzt der Arztbrief:

[ARZTBRIEF]

Das war mein Arztbrief. Bitte geben Sie nun eine einfache Zusammenfassung, die ich auch ohne Fachbegriffe verstehe und erklären Sie medizinische Abstufungen. Benutzen Sie bitte die Struktur, die ich Ihnen oben gegeben habe und Siezen Sie mich.

Task 2: General Summary

Im Folgenden gebe ich Ihnen einen Arztbrief. Ich möchte, dass Sie den Arztbrief für mich in verständlicher Sprache zusammenfassen, weil ich keine medizinischen Fachworte kenne und vieles nicht verstanden habe. Ich kenne die Diagnosen und Medikation schon. Ich möchte, dass Sie mir erklären, was während meines Aufenthaltes im Krankenhaus passiert ist. Also welche Untersuchungen gemacht wurden, was herausgefunden wurde usw. Seien Sie möglichst genau in Bezug auf die Sicherheit mit der Aussagen getroffen werden. Falls ich selbst Dinge machen muss, fügen Sie bitte ein, wie. Hier ist jetzt der Arztbrief:

[ARZTBRIEF]

Das war der Arztbrief. Bitte geben Sie nun eine einfache Zusammenfassung, die ich auch ohne Fachbegriffe verstehe. Bestätigen Sie NICHT, dass Sie das machen können. Was ist während meines Aufenthaltes im Krankenhaus passiert, bzw. was wurde gemacht?

Task 3: Action Points

Im Folgenden gebe ich Ihnen einen Ausschnitt aus einem Arztbrief. Im Ausschnitt kommen vielleicht Informationen oder Anweisungen vor, anhand derer ich mein Verhalten ändern muss. Wichtig ist, ob sich für mich etwas Wichtiges ändert oder ich etwas unbedingt beachten muss. Es könnte ja sein, dass meine Gesundheit davon abhängt. Weisen Sie mich aber auf mögliche Folgen hin, wenn ich Dinge nicht einhalte! Falls sich etwas an meiner Medikation ändert, geben Sie mir das am besten als kleine Tabelle aus. Bitte geben Sie auch Erklärungen für die Änderungen wieder, falls sie im Ausschnitt stehen. Hier ist der Ausschnitt:

[AUSSCHNITT AUS ARZTBRIEF]

Das war der Ausschnitt. Bitte geben Sie mir die wichtigen Punkte, anhand derer ich mein Verhalten ändern muss. Oder was ich unbedingt beachten sollte, um meine Gesundheit nicht zu gefährden. Vermeiden Sie bitte Fachwörter (Medikamentennamen sind aber ok). Geben Sie Ihre Antwort in verständlicher Sprache als Liste. Wenn im Ausschnitt dazu nichts steht, ist das auch okay. Bitte geben Sie auch Erklärungen für die Änderungen wieder, falls sie im Ausschnitt stehen. Es geht nur um Dinge, die sich für mich ändern oder die ich ändern muss. Weisen Sie mich aber auf mögliche Folgen hin, wenn ich Dinge nicht einhalte! Wenn keine solchen Dinge in diesem Ausschnitt stehen, antworten Sie bitte nur mit 'keine relevanten Änderungen'

3.2 Compress:

Im Folgenden gebe ich Ihnen eine Reihe von Anweisungen, in denen sich viel wiederholt. Ich möchte nicht alles selbst lesen, sondern nur die wichtigsten Punkte. Bitte wiederhole mir die wichtigsten Punkte, anhand derer ich mein Verhalten ändern muss. Bitte wiederholen Sie mir die genaue Dosierung meiner Medikamente sowie Folgetermine die ich machen sollte. Wiederholen Sie mir außerdem, was ich unbedingt beachten sollte, um meine Gesundheit nicht zu gefährden. Ich möchte, dass Sie Ihre Antwort im JSON Format als dict geben, und zwar mit folgender Struktur:\n ["Medikamente","Ernährung / Bewegung", "weitere Arztkontakte / Untersuchungen"]\n Das war die Struktur, in der Sie bitte Ihre Antwort geben, hier sind die Anweisungen:

[ALLE ACTIONPOINT SUMMARIES AUS 3.1]

Das waren die Anweisungen. Bitte wiederholen Sie die wichtigsten Punkte, die ich beachten muss oder anhand derer ich mein Verhalten ändern muss. Bitte wiederholen Sie die genaue Dosierung meiner Medikamente sowie Folgetermine, die ich machen sollte. Außerdem alles, was ich unbedingt beachten sollte, um meine Gesundheit nicht zu gefährden. Seien Sie aber bitte neutral oder ermutigend, setzen Sie mich nicht unter Druck! Weisen Sie mich aber auf mögliche Folgen hin, wenn ich Dinge nicht einhalte! Lassen Sie bitte nichts weg, meine Gesundheit hängt davon ab! Benutzen Sie bitte die Struktur, die ich Ihnen oben gegeben habe.

3.3 Adversarial Challenge

Ich möchte, dass Sie sich in meine Rolle versetzen. Ich kenne keine Fachwörter und habe keine medizinischen Kenntnisse. Vor allem kenne ich keine medizinischen Abkürzungen oder Abstufungen. Ich kenne aber die Namen der Medikamente und auch gängige Namen von Krankheiten. Wie ein BMI berechnet wird, weiß ich aber zum Beispiel nicht. Generell bin ich vom Verständnis her ungefähr auf der Stufe der achten Klasse. Im Folgenden gebe ich Ihnen einen Text. Ich möchte, dass Sie Punkte heraussuchen, die für mich schwer zu verstehen wären, falls es welche gibt. Also Punkte, die Spezialworte enthalten, oder fachliche Abkürzungen.

[COMPRESSED SUMMARY AUS 3.2]

Das war der Text. Für mich schwer zu verstehen wären die folgenden Punkte:

3.4 Rework Summary

Folgende Punkte waren in Ihrer Antwort oben schwer zu verstehen. Bitte wiederholen Sie Ihre Antwort, passen aber die schwierigen Punkte an.

[ERGEBNIS AUS 3.3]

Das waren die Punkte, die schwer zu verstehen waren, bitte wiederholen Sie Ihre Antwort, passen aber die schwierigen Punkte an. Bestätigen Sie NICHT, dass Sie das machen können, halten Sie sich bitte an die Struktur "Medikamente","Ernährung / Bewegung", "weitere Arztkontakte / Untersuchungen". Die angepasste, wiederholte Antwort ist:

**Original Prompting (English version)**

Task 1: Structured Info

In the following I will give you my discharge letter. I would like you to list the contents of the discharge letter for me in understandable language. My comprehension is is approximately that of an eighth-grade student. Please explain medical terms. I would like you to give your answer in JSON format as dict, with the following structure:\n ["When was the doctor's appointment? Where was it (practice, clinic)? How was it (outpatient or inpatient)?”, ‘Main diagnosis’, ‘Secondary diagnoses’]\n That was the structure in which you please give your answer, here is the discharge letter:

[DISCHARGE LETTER]

That was my discharge letter. Please give a simple summary that I can understand without using technical terms and explain the medical terms. Please use the structure I gave you above and address me formally.

Task 2: General Summary

In the following I will give you a discharge letter. I would like you to summarize the discharge letter for me in understandable language because I do not know any medical terminology and do not understand much of it. I already know the diagnoses and medication. I would like you to explain to me what happened during my stay in hospital. In other words, what tests were carried out, what was found out, etc. Be as precise as possible with regard to the certainty with which statements are made. If I have to do things myself, please include that. Here is the discharge letter:

[DISCHARGE LETTER]

That was the discharge letter. Now please give a simple summary that I can understand without using technical terms. Do NOT confirm that you can do this. What happened during my stay in hospital and what was done?

Task 3: Action Points

In the following I will give you an excerpt from a discharge letter. The excerpt may contain information or instructions I need to follow to change my behavior. The important thing is whether something important changes for me or whether I need to pay attention to something. It could be that my health depends on it. But make me aware of the possible consequences if I don't follow the instructions! If something changes in my medication, it is best to provide a small table. Please also include explanations for the changes if they are in the excerpt. Here is the excerpt:

[EXCERPT FROM DISCHARGE LETTER]

That was the excerpt. Please provide a comprehensive overview of the key points that I need to follow to change my behavior. Or what I should pay attention to in order not to endanger my health. Please avoid using technical terms (but drug names are ok). Give your answer in understandable language as a list. If there is nothing in the excerpt, that is also okay. Please also give explanations for the changes if they are in the excerpt. It is only about things that have changed for me or that I need to change. However, please point out possible consequences if I do not comply! If there are no such things in this excerpt, please answer only with 'no relevant changes'

3.2 Compress:

Below I give you a set of instructions that contain a lot of repetition. I don't want to read everything myself, just the most important points. Please repeat the most important points that I need to use to change my behavior. Please repeat to me the exact dosage of my medication and the follow-up appointments I should make. Please also tell me what I should pay attention to in order not to endanger my health. I would like you to give your answer in JSON format as dict, with the following structure:\n [“medication”, “diet / exercise”, “further doctor contacts / examinations”]\n That was the structure in which you please give your answer, here are the instructions:

[ALL ACTIONPOINT SUMMARIES FROM 3.1]

These were the instructions. Please repeat the most important points that I need to consider or that I need to use to change my behavior. Please repeat the exact dosage of my medication and any follow-up appointments I should make. Also, anything that I should be aware of in order not to endanger my health. But please be neutral or encouraging, don't put me under pressure! But point out possible consequences if I don't follow the instructions! Please use the structure I have given you above.

3.3 Adversarial Challenge

I would like you to to see things from my perspective. I don't know any technical terms and have no medical knowledge. Above all, I don't know any medical abbreviations or gradations. However, I do know the names of medications and common names of diseases. I don't know how a BMI is calculated, for example. In general, my level of understanding is around that of an eighth-grade student. I will give you a text below. I would like you to pick out points that would be difficult for me to understand, if there are any. In other words, points that contain special words or technical abbreviations.

[COMPRESSED SUMMARY FROM 3.2]

That was the text. The following points would be difficult for me to understand:

3.4 Rework Summary

The following points were difficult to understand in your answer above. Please repeat your answer, but adjust the difficult points.

[RESULT FROM 3.3]

These were the points that were difficult to understand, please repeat your answer but adjust the difficult points. Do NOT confirm that you can do this, please stick to the structure “medication”, “diet/exercise”, “further doctor contacts/examinations”. The adapted, repeated answer is:

**Supplementary Material 3: Exemplary patient letters generated by GPT-4**

**a) Arterial Hypertension**

Dear Ronny Racer,

## Time and place of treatment

Mr Racer went to an outpatient clinic for kidney disease for a check-up. This visit was without an overnight stay, i.e. on an outpatient basis.

## Main diagnosis

Mr Racer has high blood pressure (hypertension), which is not easy to get rid of and is quite severe (grade II, which means moderate).

## Secondary diagnoses

There was no evidence yet for other causes of high blood pressure. A special investigation into this is still awaited.

The high blood pressure could damage the kidneys, there is a bit of protein in the urine (albuminuria).

There used to be problems with the esophagus after the entrance to the stomach due to acid (reflux esophagitis).

A knee was once examined with a special camera (arthroscopy).

Mr Racer also has too much cholesterol in his blood.

He is allergic to certain medications (codeine and azithromycin).

## What happened at the hospital?

During your hospitalization, the doctors tried to find out why your blood pressure is so high. This is called hypertension. To rule out other diseases that can lead to high blood pressure, they have carried out various tests:

1. You had an MRI (a special type of X-ray where you can see the body very clearly) to see if the blood vessels leading to your kidneys were narrow. But this was not the case.

2. They collected your urine and analyzed it for various substances, such as cortisol, which is a hormone that the body produces in stressful situations. But that was also all normal.

3. You have had blood taken to measure whether your kidneys are functioning properly and whether your thyroid levels are OK. This was also all normal.

4. They also analyzed your urine to see if it contained too much protein, which could be a sign that your kidneys are already a little damaged by the high blood pressure. Here they found that there was indeed a little too much protein in your urine.

Ultrasound examinations of the kidneys and adrenal glands (a technique that uses sound waves to look inside the body) revealed no abnormalities that would explain the high blood pressure.

You have told us that you have often had higher blood pressure after a car accident and that your father has had a stroke, which means that the risk of such problems may be a little higher in your family.

The doctors also talked to you about how much you drink, how much coffee you drink, your sleeping behavior and whether you smoke or drink alcohol, all of which you denied. However, they said your sleep is not restful and you snore, which could indicate that you may have a problem with breathing while sleeping. You also said that your blood pressure is still a bit high at home, even after taking medication for it.

The doctors now want you to have another urine test: you must have your urine collected and analyzed within 24 hours to make sure that you do not have a rare adrenal disease.

You have also been given a new combination of medication and should take this regularly and make sure that you avoid certain foods such as grapefruit juice, as this can affect the effect of the medication.

The doctors recommend that you continue to check your blood pressure regularly and write it down to check whether the medication is helping. If the pressure rises very high, you should go straight to the doctor. You should also try to reduce your weight, eat more healthily (lots of fruit and vegetables, less fatty dairy products) and use less salt. If you follow this advice, it can also help to lower your blood pressure.

## What do you need to consider now?

Medication
- Take a daily combination of antihypertensive medication consisting of ramipril, amlodipine and an additional medication to flush out excess water (hydrochlorothiazide). The dose is 10 mg ramipril, 10 mg amlodipine and 25 mg hydrochlorothiazide (10/10/25 mg) once in the morning.

- In addition, take one tablet of pantoprazole (20 mg) each morning to protect your stomach.

Ramipril/Amlodipine/HCT 10/10/25 mg
1 - 0 - 0, NEW
Ramipril 5 mg
1 - 0 - 1, FINISHED

Amlodipine 10 mg
1 - 0 - 0, FINISHED
Pantoprazole 20 mg
1 - 0 – 0

Information for medication:

- Check your blood pressure regularly and write down the values.

- Ideally, your blood pressure should be below 130/80.

- If your blood pressure is very high (180/120 or higher), you should see a doctor immediately.
 - Avoid foods or drinks that could affect the effect of your blood pressure medication, such as grapefruit juice.

Diet / exercise

- Eat healthily according to the principles of the DASH diet, which includes lots of fruit and vegetables and is good for your blood pressure.

- Reduce the amount of salt in your diet to a maximum of 5-6 grams a day.

- Try to keep your weight in a healthy range, which corresponds to a body mass index (BMI) of 18.5 to 24.9.

Further medical contacts / investigations

- Make an appointment for a check-up to see if you are breathing normally during sleep and there are no interruptions (sleep apnea).

- Also do a special urine test where urine collected over 24 hours is tested for certain stress hormones (metanephrines)

- this will help to find out if you may have a rare tumor on the adrenal glands (pheochromocytoma).

Advice for investigations:

- It is important that you have further investigations carried out to ensure that any potential health problems can be recognized and treated.

Please be sure to discuss your new medication plan with your family doctor.

**b) Diabetes mellitus**

Dear Sebastian Sweet,

## Time and place of treatment

Sebastian Sweet was admitted to hospital in October 2023 due to an initial diagnosis of type 2 diabetes. The treatment took place on an inpatient basis, meaning that Mr Sweet had to stay in hospital.

## Main diagnosis

Mr Sweet found out for the first time that he has type 2 diabetes. This means that the sugar level in his blood is permanently too high. He was diagnosed with extremely high blood sugar levels. He also had symptoms such as weight loss and frequent urination.

## Secondary diagnoses

Coxarthrosis on the left: Mr Sweet has joint wear and tear in his left hip (coxarthrosis), i.e. the hip joint is worn out.

Status post appendectomy 1972: Mr Sweet underwent pancreatic surgery as a young man.

Status post onychomycosis on both sides: Mr Sweet had fungal nail disease on both feet.

Hay fever: Mr Sweet is allergic to pollen, this is called hay fever.

## What happened at the hospital?

During your hospitalization, you were treated because your blood sugar was very high and you were therefore diagnosed with type 2 diabetes. This is a disease in which the sugar in your blood cannot enter the cells properly, which leads to high sugar levels in the blood.

The doctors have noticed that you have lost a lot of weight recently and that you have to urinate very often, both of which can be signs of diabetes. Your tests have shown that your HbA1c value, which indicates how high your blood sugar has been on average in recent weeks, was far too high (14.1%). A healthy value is below 6%. They did blood tests that looked for specific proteins (antibodies) to rule out the possibility that it was a different type of diabetes. These tests were negative. An ultrasound scan of the pancreas area was also performed to rule out other possible conditions there. There was no indication of any unusual changes.

You have been prescribed insulin, which is a hormone that helps to bring sugar into the cells and thus lower blood sugar. In addition, you are taking tablets called metformin and empagliflozin, which also help to regulate your blood sugar.

The doctors have also discovered that you have nerve damage in your feet, which is common in diabetics. It is important that you regularly examine your feet yourself to avoid further problems.

As your blood lipid levels were also too high, you were prescribed a drug to lower them and protect your heart. The medication is called atorvastatin.

The doctors have also advised you to visit certain specialists regularly to ensure that your eyes, heart and kidneys are not damaged by the diabetes.

You should check your blood glucose levels at home and pay attention to how much insulin you need to inject. If your values in the morning before breakfast are above 150 mg/dl for three days in a row, you should take a little more insulin. If they are below 100 mg/dl, you should take less.

It is important that you reduce your alcohol consumption, as alcohol can affect blood sugar levels and increase the risk of other health problems.

Doctors have also recommended that you exercise regularly to stay healthy and reduce the risk of heart disease.

You were doing well when you were discharged, but you should follow the doctors' recommendations to ensure that you continue to do well and to avoid complications from the diabetes.

## What do you need to consider now?

Medication:
- Metformin: Take one tablet (500 mg) in the morning and again in the evening, this helps to lower blood sugar.

- Empagliflozin: Take one (10 mg) tablet every morning - it helps your kidneys to get rid of excess sugar from the blood via the urine.

- Insulin glargine: Inject 14 units every morning at 8am, the insulin helps your body to utilise and store blood sugar.

- Atorvastatin: One (20 mg) tablet in the morning can help keep your cholesterol low and protect your heart.

Metformin 500 mg 1-0-1, NEW
Empagliflozin 10 mg 1-0-0, NEW
Insulin glargine 100 E/ml 14 IU at 8 a.m., NEW
Atorvastatin 20 mg 1-0-0, NEW

Insulin dose adjustment:

- If your blood sugar is above 150 mg/dl in the morning, before breakfast, for three days in a row, increase your insulin dose by 2 units.

- If your blood sugar is below 100 mg/dl in the morning, reduce your insulin dose by 4 units.

Diet / exercise:

- Try to drink less alcohol - it's better for your blood sugar.

- Eat foods that are good for diabetics: fewer sweet things and more whole meal products instead, such as whole meal bread or pasta, and plenty of vegetables, which keep you fuller for longer and are good for your blood sugar.

- Stay active by going for a walk or other moderate exercise for about 30 minutes most days of the week, this will help your body deal with the sugar.

Further doctor contacts / examinations:

- Your long-term blood sugar level, the HbA1c, should be checked every three months - it should be between 7.5 % and 8 % without you often having too low blood sugar levels (hypoglycemia).

- It's important to have your eyes, heart and kidneys checked regularly by a specialist to make sure everything is in order.

- Look at your feet daily to notice blisters, cuts or changes early so they don't get worse.

- Pay attention to these things so you can prevent serious problems such as heart disease or diabetic foot syndrome, which are serious foot problems caused by diabetes.

Please be sure to discuss your new medication plan with your family doctor.

**c) Diabetic nephropathy**

Dear Marion Mason,

## Time and place of treatment

The examinations and kidney biopsy took place in hospital. These treatments were not outpatient, which means that you had to stay in hospital for at least one night.

## Main diagnosis

You have an advanced stage of chronic kidney disease caused by diabetes and high blood pressure. Your kidney function is no longer so good and there is too much protein in your urine.

## Secondary diagnoses

Diabetes mellitus type 2 since 2017 - This is the type of diabetes that usually occurs in adults and in which your body has problems regulating blood sugar.

Very overweight (obesity II°) - This means that your body weight is much higher than would be good for your health.

High blood pressure since 2017 - This means that the pressure in your blood vessels is permanently too high.

Artificial right knee joint since May 2019 (Status post knee TEP) - You have had a new knee joint because the old one was severely damaged by a skiing accident and wear and tear (osteoarthritis).

## What happened at the hospital?

At the hospital, the doctors carried out a thorough examination of your kidneys because they were no longer functioning properly and because more protein than normal was found in your urine. To do this, they took a kidney biopsy, in which they removed a small piece of tissue from your kidney to look at it under the microscope.

Doctors have discovered that the poor function of your kidneys is due to long-term damage caused by diabetes and high blood pressure. The damage affects the small filtering units in the kidneys (the so-called glomeruli), as well as the blood vessel walls and the supporting tissue of the kidneys. The biopsy result showed that about 40% of your kidney tissue is permanently altered.

You have also been diagnosed with a high blood glucose level, which shows that your diabetes is not optimally controlled. The doctors have therefore adjusted the dose of metformin, your diabetes medication, and added a new medication (an SGLT2 inhibitor).

You also have high blood pressure; therefore, the doctors have adjusted the dosage of your blood pressure medication. In addition to your normal blood pressure medication, you have also been given a diuretic (water tablet) to reduce fluid retention in your legs.

The doctors have said that you should regularly measure your blood pressure at home to ensure that the new medication is working and that your blood pressure remains stable. It is also important that you lose weight because excess weight puts additional strain on the kidneys. A Mediterranean diet has been recommended to help you lose weight.

Finally, you were advised to avoid anti-inflammatory drugs such as ibuprofen because they can worsen the condition of your kidneys. It was also pointed out that you should be especially careful with infections and fever and pause certain medications to avoid serious side effects.

Examinations and treatment in hospital have focused on assessing the condition of your kidneys and adjusting your medication to keep your health stable. Now that you have been discharged, you should continue to see a kidney specialist who will regularly review your case and the progress of your condition.

## What do you need to consider now?

Medication:
- Ramipril/Amlodipine: These are medications that help to lower blood pressure. You take one tablet in the morning and one in the evening.

- HCT: This medication helps to remove excess water from the body and lower blood pressure. You take one tablet in the morning.

- Metformin: This is a medication for people with diabetes (type 2 diabetes). It helps to control the sugar level in the blood. One tablet in the morning and one in the evening.

- Forxiga: This medication also helps with type 2 diabetes and ensures that excess sugar is excreted via the urine. You take one tablet in the morning.

- Torasemide: This also helps to remove water from the body and lowers blood pressure. You take one tablet in the morning.

- Atorvastatin: You use this to lower the cholesterol level in your blood, which is important for the health of your heart. One tablet comes in the evening.

- Vigantoletten: These are tablets containing vitamin D, which is important for your bones and immune system. One tablet in the morning.

Ramipril/amlodipine 5 mg/5 mg
2 - 0 - 0, INCREASED, was 1 - 0 - 0
HCT 12.5 mg
1 - 0 - 0
Metformin 500 mg
1 - 0 - 1, REDUCED, was 2 - 0 - 2

Forxiga 10 mg
1 - 0 - 0, NEW
Torasemide 20 mg
1 - 0 - 0, reduce in the course
Atorvastatin 40mg
0 - 0 - 1
Vigantoletten 1000IE
1 - 0 - 0

Diet / exercise:

- Fluid intake: Drink about 2 liters of water a day unless your doctor says otherwise.

- Weight management: Try to reduce your body weight and stick to a healthy diet with lots of fruit, vegetables and little fat - this is called a "Mediterranean diet".

- Dietary measures: Eat healthy to control your weight and prevent swelling caused by too much fluid in the body.

- Alcohol/nicotine consumption: You should avoid alcohol and cigarettes to protect your kidneys and the rest of your body.

Other doctor contacts / examinations:

- Nephrological check-up: This means that you go to the kidney doctor regularly, every 3 months, to make sure that your kidneys are working well.

- Blood pressure monitoring: Measure your blood pressure regularly at home to ensure that it remains within the normal range.

- Allergy advice: Let any doctor know that you are allergic to penicillin so that you don't accidentally get this medication because it could make you unwell.

- Observation: Pay attention to changes in urination, for example whether the urine foams. If this happens, it is important that you tell your doctor.

Please be sure to discuss your new medication plan with your family doctor.

**Supplementary Material 4: Learning objectives**

**Learning objectives AHT**

| **Thematic category** | **Learning content** | **Learning goal** | **Bloom category** |
| --- | --- | --- | --- |
| Organizational | Conducting a 24-hour urine collection to exclude hormonal causes of hypertension. | 1.1 The patient knows that he/she has to conduct a 24-hour urine.  1.2 The patient understands that the 24-hour urine is needed to exclude hormonal causes of hypertension. | Remember  Understand |
|  | Undergoing ambulatory sleep apnea diagnostics to investigate its potential association with hypertension. | 2.1. The patient knows that he/she has to undergo ambulatory sleep apnea diagnostics.  2.2 The patient understands that sleep apnea might be associated with hypertension. | Remember  Understand |
| Medication | Discontinuing Ramipril and Amlodipine, as they are included in the new combination tablet. | 3.1 The patient knows that he/she has to discontinue Ramipril and Amlodipine.  3.2 The patient understands that Ramipril and Amlodipine are included in the new combination tablet. | Remember  Understand |
|  | Taking a fixed-dose combination (Ramipril/Amlodipine/HCT) for optimizing blood pressure control | 4.1 The patient knows that he/she has to take the fixed-dose combination.  4.2 The patient understands that the fixed-dose combination is used to optimize blood pressure control. | Remember  Understand |
| Prevention of complications | Avoiding simultaneous intake of the combination tablet with grapefruit juice in order to prevent interactions. | 5.1 The patient knows that he/she has to avoid simultaneous intake of the combination tablet with grapefruit juice.  5.2 The patient understands that the combination tablet interacts with grapefruit juice. | Remember  Understand |
|  | Being vigilant regarding allergies to Codeine and Azithromycin to prevent inadvertent prescription and potential allergic reactions | 6.1 The patient knows that he/she has to inform doctors about his/her allergy to Codeine and Azithromycin in future visits.  6.2 The patient understands that this information helps prevent accidental prescription of these substances. | Remember  Understand |
|  | Achieving a target blood pressure below 130/80 mmHg to prevent cardiovascular damage. | 7.1 The patient knows that he/she has to achieve a target blood pressure below 130/80 mmHg.  7.2 The patient understands that this measure helps to prevent cardiovascular damage. | Remember  Understand |
|  | Seeking immediate medical attention when blood pressure readings exceed 180/120 mmHg to prevent acute organ damage. | 8.1 The patient knows that he/she has to seek immediate medical attention when blood pressure readings exceed 180/120 mmHg.  8.2 The patient understands that this measure helps to prevent acute organ damage. | Remember  Understand |
| Disease Management/ Lifestyle changes | Monitoring blood pressure at home regularly and documenting the readings in order to help doctors to adjust blood pressure therapy. | 9.1 The patient knows that he/she has to regularly monitor blood pressure at home and document readings.  9.2 The patient understands that the readings help doctors to adjust blood pressure therapy. | Remember  Understand |
|  | Changing dietary habits consistent with a DASH diet (abundant fruits and vegetables, reduced-fat dairy) to optimize blood pressure values. | 10.1 The patient knows that he/she has to change dietary habits consistent with a DASH diet.    10.2 The patient understands that a DASH diet helps to optimize blood pressure values. | Remember  Understand |
|  | Striving for weight reduction to achieve a target BMI of 18.5-24.9 kg/m² for the optimization of blood pressure. | 11.1 The patient knows that he/she has to strive for weight reduction to achieve a target BMI of 18.5-24.9 kg/m².  11.2 The patient understands that a weight reduction helps to optimize blood pressure values. | Remember  Understand |
|  | Reduction of salt intake to a maximum of 5-6 g/day to optimize blood pressure values. | 12.1 The patient knows that he/she has to reduce salt intake to a maximum of 5-6 g/day.  12.2 The patient understands that a reduction of salt helps to optimize blood pressure values. | Remember  Understand |

**Learning objectives DKD**

| **Thematic category** | **Learning content** | **Learning goal** | **Bloom category** |
| --- | --- | --- | --- |
| Organizational | Have regular follow-up checks every 3 months with an outpatient nephrologist to monitor the progression of the kidney disease. | 1.1The patient knows that he/she has follow-up checks every 3 months  1.2 The patient understands the necessity of regular appointments for monitor the progression of kidney disease. | Remember  Understand |
| Medication | Increase the dose of Ramipril/Amlodipine to lower blood pressure. | 2.1 The patient knows that he/she has to increase his/her dose of medication Ramipril/Amlodipine.  2.2 He understands that it leads to a lower blood pressure. | Remember  Understand |
|  | Start taking Forxiga to optimize blood sugar levels and protect the kidneys. | 3.1 The patient knows that he/she has to start taking Forxiga.  3.2 The patient understands that Forxiga is used to optimize blood sugar levels and protect the kidneys. | Remember  Understand |
|  | Start taking Torasemide for diuresis in case of lower leg edema. | 5.1 The patient knows that has started taking Torasemide.  5.2 The patient understands that Torasemide is used for diuresis to treat lower leg edema. | Remember  Understand |
| Complication Prevention | Reduce Metformin, as it needs to be adjusted according to kidney function. | 4.1 The patient knows that the dose of Metformin has been reduced.  4.2 The patient understands that the reduction is necessary to adjust the medication according to the kidney function. | Remember  Understand |
|  | NSAIDs like Ibuprofen should be avoided (used previously for knee pain) to prevent kidney damage caused by them. | 6.1 The patient knows to avoid NSAIDs such as Ibuprofen, especially those previously used for knee pain.  6.2 The patient understands that avoiding these NSAIDs is important to prevent kidney damage. | Remember  Understand |
|  | Pause SGLT2 inhibitors and Metformin during infections ("Sick Day Rules"), as otherwise undesirable side effects might occur. | 7.1 The patient knows that he/she has to pause SGLT2 inhibitors and Metformin during infections as per the 'Sick Day Rules'.  7.2 The patient understands the that this pause is necessary to prevent undesirable side effects during infections. | Remember  Understand |
|  | Consider the intolerance of GLP1 analogs as well as an allergy to penicillin in future doctor visits, so they are not accidentally prescribed. | 8.1 The patient knows to inform doctors about his intolerance to GLP1 analogs and allergy to penicillin.  8.2 The patient understands that this information helps prevent accidental prescription of these substances. | Remember  Understand |
|  | Gradually reduce Torasemide in the course when a balanced volume status is achieved, to avoid kidney damage due to volume deficit. | 9.1 The patient understands the importance of gradually reducing Torasemide when a balanced volume status is achieved.  9.2 The patient knows, that volume deficit can lead to kidney damage. | Remember  Understand |
| Disease Management/ Lifestyle changes | Regular home blood pressure monitoring with written documentation, as this can help the MDs assess whether blood pressure control has been effective. | 10.1 The patient understands that he should regularly monitor his blood pressure at home.  10.2 The patient knows that this practice helps MDs to adjust blood pressure therapy. | Remember  Understand |
|  | Weight reduction aiming for a BMI < 30 kg/m² (target weight around 82 kg), as this is protective for blood sugar levels and kidney function. | 11.1 The patient knows, he has to achieve weight reduction with the goal of attaining a BMI < 30 kg/m², with a target weight of approximately 82 kg.  11.2 The patient is aware that this approach is protective for blood sugar levels and kidney function. | Remember  Understand |
|  | Adherence to a Mediterranean diet to achieve the desired weight loss. | 12.1 The patient knows about the principles of the Mediterranean diet.  12.2 The patient understands the significance of adhering to a Mediterranean diet as a means to attain the desired weight loss. | Remember  Understand |

**Learning objectives DM**

| **Thematic category** | **Learning content** | **Learning goal** | **Bloom category** |
| --- | --- | --- | --- |
| Organizational | Scheduling appointments with an ophthalmologist, nephrologist and cardiologist to undergo examinations for potential diabetes-related complications. | 1.1 The patient knows that he/she has to schedule appointments with an ophthalmologist, nephrologist and cardiologist.  1.2 The patient understands that these appointments are necessary to undergo examinations for potential diabetes-related complications. | Remember  Understand |
|  | Arranging a follow-up appointment with the primary care physician in three months for the purpose of monitoring average blood sugar levels. | 2.1 The patient knows that he/she has to arrange a follow-up appointment with the primary care physician in three months.  2.2 The patient understands that this appointment has the purpose to monitor long-term blood sugar levels. | Remember  Understand |
| Medication | Taking two new tablets (Metformin, Forxiga) to effectively control blood sugar levels. | 3.1 The patient knows that he/she has to take two new tablets (Metformin, Forxiga).  3.2 The patient understands that these tablets are used to control blood sugar levels. | Remember  Understand |
|  | Conducting daily insulin injections for optimal blood sugar control. | 4.1 The patient knows that he/she has to conduct daily insulin injections.  4.2 The patient understands that insulin is used to control blood sugar levels. | Remember  Understand |
|  | Following the prescribed dosage increase of Atorvastatin to improve lipid control. | 5.1 The patient knows that he/she has to follow the prescribed dosage increase of Atorvastatin.  5.2 The patient understands that this dosage increase helps to control blood lipid levels. | Remember  Understand |
| Complication Prevention | Adjusting the dosage of Insulin glargine based on measured blood sugar levels to prevent hyper- or hypoglycemic episodes. | 6.1 The patient knows that he/she has to adjust the dosage of Insulin glargine based on measured blood sugar levels.  6.2 The patient understands that this adjustment helps to prevent hyper- or hypoglycemic episodes. | Remember  Understand |
|  | Being vigilant of symptoms such as palpitations, trembling or changes in consciousness to identify hypoglycemic events. | 7.1 The patient knows that he/she has to vigilant of symptoms such as palpitations, trembling or changes in consciousness.  7.2 The patient understands that these symptoms might be signs of hypoglycemia. | Remember  Understand |
|  | Seeking medical attention if experiencing muscle pain after the dosage increase of Atorvastatin, as this could be a potential side effect. | 8.1 The patient knows that he/she has to seek medical attention if experiencing muscle pain after the dosage increase of Atorvastatin.  8.2 The patient understands that muscle pain might be a potential side effect of Atorvastatin. | Remember  Understand |
| Disease Management/ Lifestyle changes | Establishing a routine of regular blood sugar checks (1-2 times weekly) to guide insulin therapy. | 9.1 The patient knows that he/she has to establish a routine of regular blood sugar checks (1-2 times weekly).  9.2 The patient understands that blood sugar checks help to guide insulin therapy. | Remember  Understand |
|  | Conducting regular foot examinations to detect early signs of diabetes-related foot damage. | 10.1 The patient knows that he/she has to conduct regular foot examinations.  10.2 The patient understands that regular foot examinations are needed to detect early signs | Remember  Understand |
|  | Engaging in moderate physical activity for at least 150 minutes per week to lower cardiovascular risk. | 11.1 The patient knows that he/she has to engage in moderate physical activity for at least 150 minutes per week.  11.2 The patient understands that moderate physical activity lowers cardiovascular risk. | Remember  Understand |
|  | Reducing daily alcohol consumption to mitigate the risk of life-threatening hypoglycemic events. | 12.1 The patient knows that he/she has to reduce daily alcohol consumption.    12.2 The patient understands that a reduction of daily alcohol consumption helps to mitigate the risk of life-threatening hypoglycemic events. | Remember  Understand |
