# Supplementary figures and images for: What’s Going On With Me and How Can I Better Manage My Health? The Potential of GPT-4 to Transform Discharge Letters Into Patient-Centered Letters to Enhance Patient Safety: Prospective, Exploratory Study
Source: J Med Internet Res. 2025 Jan 21;27:e67143. doi: 10.2196/67143 (PMC11795158; doi:10.2196/67143)

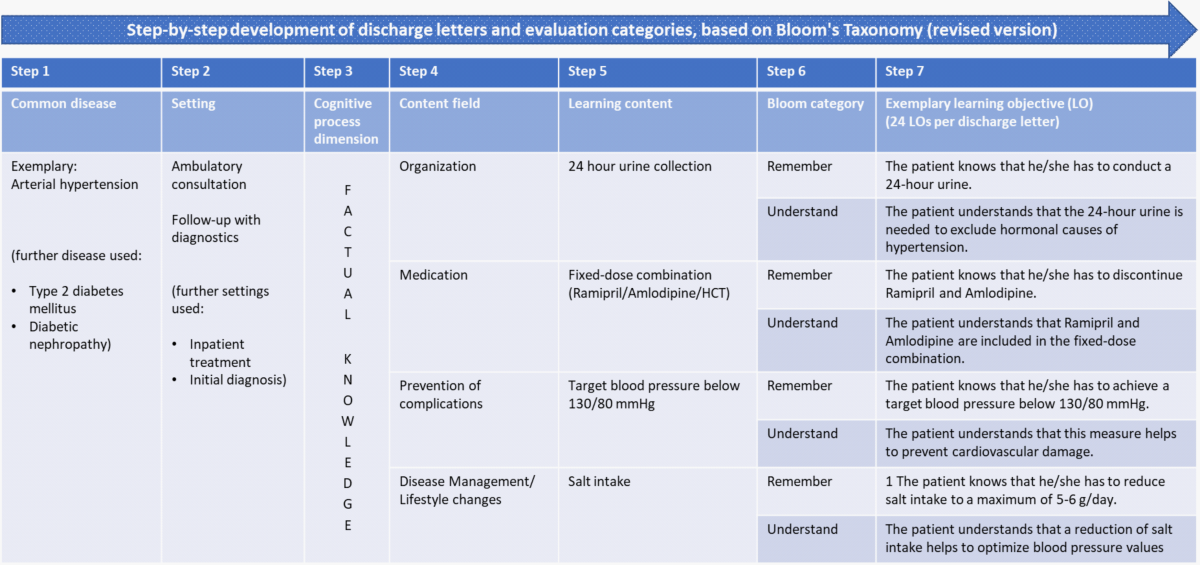

Supplement: Multimedia Appendix 2 [file jmir_v27i1e67143_app2.png]

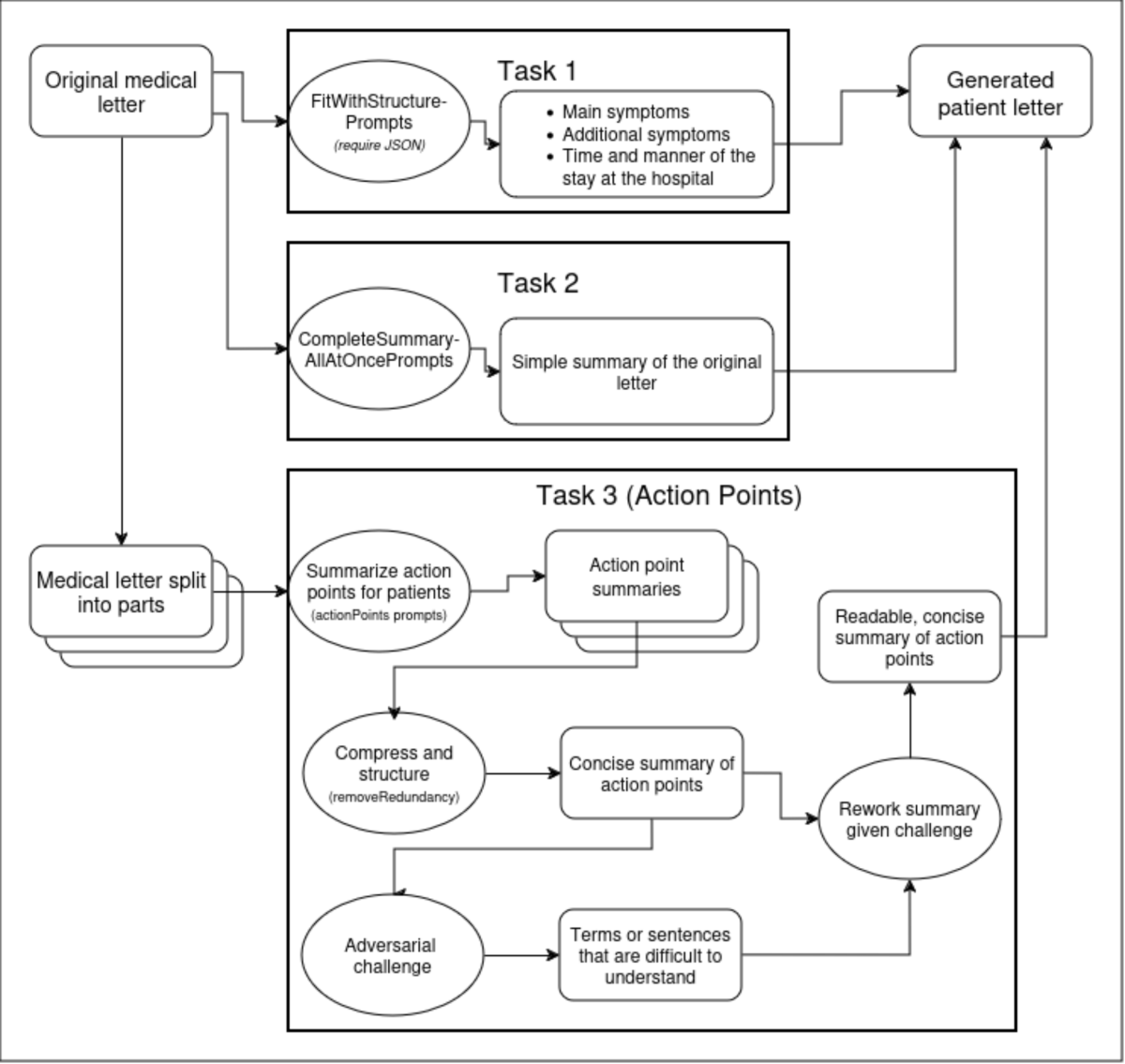

Supplement: Multimedia Appendix 3 [file jmir_v27i1e67143_app3.png]
