# Supplementary material for: What’s Going On With Me and How Can I Better Manage My Health? The Potential of GPT-4 to Transform Discharge Letters Into Patient-Centered Letters to Enhance Patient Safety: Prospective, Exploratory Study
Source: J Med Internet Res. 2025 Jan 21;27:e67143. doi: 10.2196/67143 (PMC11795158; doi:10.2196/67143)
Supplement: Multimedia Appendix 4 [file jmir_v27i1e67143_app4.docx]

| **Bloom category** | **Common disease** | **Learning objective (information added for better understanding)** | **No of patient letters in which learning objective is missing (x/5)** | **Content field** | **Recommendations for Prompt Engineering** |
| --- | --- | --- | --- | --- | --- |
| Remember | Type 2 diabetes mellitus | being vigilant of palpitations, trembling or changes in consciousness | 4 | **Prevention of complications** |  |
|  |  | arrange follow up with primary care physician every 3 months | 3 | Organizational |  |
| **Understand** | Arterial hypertension | (target blood pressure below 130/80 mmHg) prevents cardiovascular damage | 5 | **Prevention of complications** | The idea about providing expected structure for an action point should also help here to some extent. This structure would need to include a subcategory that explains the “why”.  An example of such a structure in the prompt which includes information from the Understand category may help further. |
|  |  | (discontinue ramipril and amlodipine) included in the new fix-dose combination | 4 | Medication |  |
|  |  | (inform doctors about his/her allergy to codeine and Azithromycin) prevent accidental prescription of these substances | 3 | **Prevention of complications** |  |
|  |  | (ambulatory sleep apnea diagnostics sleep apnea) might be associated with hypertension | 3 | Organizational |  |
|  | Type 2 diabetes mellitus | (regular blood sugar checks) help guide insulin therapy | 4 | Disease Management/ Lifestyle changes |  |
|  |  | (being vigilant of palpitations, trembling or changes in consciousness) these are symptoms of hypoglycemia | 4 | **Prevention of complications** |  |
|  |  | (dosage of insulin based on blood sugar levels) prevents hyper- or hypoglycemic episodes | 3 | **Prevention of complications** |  |
|  | Diabetic nephropathy | (reducing Torasemide when a balanced volume status is achieved) knows that volume deficit can lead to kidney injury | 5 | **Prevention of complications** |  |
|  |  | (Reduce Metformin) needs to be dose-adjusted according to kidney function | 4 | **Prevention of complications** |  |
