# Supplementary material for: What’s Going On With Me and How Can I Better Manage My Health? The Potential of GPT-4 to Transform Discharge Letters Into Patient-Centered Letters to Enhance Patient Safety: Prospective, Exploratory Study
Source: J Med Internet Res. 2025 Jan 21;27:e67143. doi: 10.2196/67143 (PMC11795158; doi:10.2196/67143)
Supplement: Multimedia Appendix 5 [file jmir_v27i1e67143_app5.docx]

| Content field | Category | Examples | Recommendations for Prompt Engineering |
| --- | --- | --- | --- |
| Organizational | Lack of connection to the underlying disease | "Additionally, you should perform a special urine collection test at home, where your urine will be collected over a 24-hour period to search for additional hormones that may indicate other diseases." | (See idea about providing structure below) |
|  | Information is missing that would enable specific actions: | | |
|  | - Responsibility | "Every three months, one should have a blood test called HbA1c, which shows the average blood sugar level over the past few months." | Clearer definition of the context in which the patients operate (here, the German health care system). This makes the prompt longer, potentially hurting performance elsewhere. |
|  | - Frequency | "Eye doctor visits: Have your eyes checked regularly to ensure that your vision is not affected by diabetes." |  |
| Medication | Information missing: | | |
|  | - Dosage | "If your levels are above 150 mg/dl before breakfast on three consecutive days, you should take a bit more insulin." | Provide a clear expected structure for an action point:  1. required action  2. how to judge / measure success  3. what to do in case of bad  measurements  4. why this is important  5. what happens if unchecked  Provide one example of this expected structure |
|  | - Medication | "It has also been found that your cholesterol level is too high, and you have been started on a medication that can additionally reduce the risk of heart problems." |  |
| Prevention of complications | Information missing: |  |  |
|  | - Threshold | "If the pressure rises very high, you should go to the doctor immediately." |  |
|  | - recommended action for side effects | "You should watch for muscle pain, as this can be a side effect of the medication." |  |
| Lifestyle/Disease management | Information is missing that would support specific actions | |  |
|  | - target value | "You should also adjust your diet, eat less salt, and make sure you do not weigh too much." |  |
|  | - Frequency | "Additionally, you have been advised to exercise regularly to improve your overall health.” |  |

^a^Examples of learning objectives that have been partially listed in the patient-centered letters including recommendations for further prompt engineering.
