# Supplementary material for: What’s Going On With Me and How Can I Better Manage My Health? The Potential of GPT-4 to Transform Discharge Letters Into Patient-Centered Letters to Enhance Patient Safety: Prospective, Exploratory Study
Source: J Med Internet Res. 2025 Jan 21;27:e67143. doi: 10.2196/67143 (PMC11795158; doi:10.2196/67143)
Supplement: Multimedia Appendix 6 [file jmir_v27i1e67143_app6.docx]

| **Content field (counts)** | **Content** | **Unit of information, patient letter** | **Source (discharge letter)** | **Error category** | **Possible causes** |
| --- | --- | --- | --- | --- | --- |
| **Disease management/**  **Lifestyle (6)** | Therapy success monitoring/  Type of examination | "Your blood sugar should be checked every three months to ensure that the medications are working properly." | "HbA1c checks every three months." | imprecise | Mixing of different therapy monitoring examinations |
|  | Therapy success monitoring/  Frequency of examination | "Monitor your blood sugar levels closely to ensure they are not too high or too low." | "During the therapy, it is recommended to perform fasting blood sugar checks once or twice a week to guide treatment management." | imprecise |  |
|  | Dietary measures | "Diet: Be sure to reduce your intake of salt, protein, and potassium to ease the strain on your kidneys." | "We recommend following a Mediterranean diet for this purpose." | incorrect (for this stage of the disease) | Recommendation not appropriate for this stage of the disease |
| **Medical knowledge (8)** | Explanation/  Definition of a disease | "The HbA1c value, which indicates the average blood sugar level over the past few weeks, is at 14.1%, which is also very high (normal is under 6%)." | No information available | incorrect |  |
|  | Explanation/  Definition of a disease | "Previously, there were issues with the esophagus just after the stomach due to acid (reflux esophagitis)." | "History of reflux esophagitis" | incorrect |  |
|  | Deduction of the most likely risk factor based on the disease | "The family also has a history of blood pressure issues, and his father had a stroke: This could indicate that Mr. Raser is at an increased risk for cardiovascular diseases." | "CVRF: Positive family history of a stroke in the father at the age of approximately 50-60 years." | presumptive | Independent inference of the most likely risk factor |
|  | Interpretation of a test result | "They also tested to see if your pancreas has any issues, which it does not." | "An ultrasound of the pancreas showed no evidence of a mass." | presumptive | Improper generalization (due to intended simplification?) |
| **medication (7)** | Route of administration | "Insulin glargine: Take 14 'pumps' of this special diabetes medication every morning to keep your blood sugar under control." | "Insulin glargine 100 U/mL: 14 units at 8 a.m., NEW." | incorrect | Incomplete medication plan (missing method of administration) in the letter and improper simplification (possibly due to intended simplification?) |
|  | Time of administration | "Medications: Ramipril/Amlodipine: Now take these blood pressure tablets twice daily." | "Ramipril/Amlodipine 5 mg/5 mg: 2 – 0 – 0, INCREASED, previously 1 – 0 – 0" | incorrect |  |
|  | Medication adjustment | "You take this tablet every morning, and it replaces your previous medications, namely Ramipril and Amlodipine at a lower dosage." | "Ramipril/Amlodipine/HCT 10/10/25 mg: 1 – 0 – 0, NEW Ramipril 5 mg: 1 – 0 – 1, DISCONTINUED Amlodipine 10 mg: 1 – 0 – 0, DISCONTINUED" "...thus, we recommend a therapy adjustment as indicated above..." | incomplete |  |
| **Prevention of complications (3)** | Sick day rules | "If you develop a fever or an infection, you should temporarily discontinue a specific diabetes medication to avoid severe side effects.” | "In the event of an infectious disease with fever, the therapy with the SGLT2 inhibitor, as well as the therapy with metformin, should be paused due to the increased risk of ketoacidosis or lactic acidosis." | incomplete | Multidimensional problem which requires adherence to a strict process chain. |
|  | Allergy | "It is important that you see a doctor immediately at the first sign of an infection, especially since you have an allergy to penicillin." | "Please exercise increased vigilance for penicillin allergy during a doctor's visit in case of infection." | incorrect |  |
| **Organizational (3)** | Incorrect assignment of time | "Mr. Süss was treated as an inpatient, staying overnight in a hospital on October 8, 2023, after visiting a general practitioner." | "Admission: 04/10/2023 /  Discharge: 08/10/2023" | incorrect |  |
